# Supplementary material for: Therapeutic potential of targeting microRNA‐10b in established intracranial glioblastoma: first steps toward the clinic
Source: EMBO Mol Med. 2016 Feb 10;8(3):268–87. doi: 10.15252/emmm.201505495 (PMC4772951; doi:10.15252/emmm.201505495)
Supplement: Supplementary file 1 — Appendix [file EMMM-8-268-s001.pdf]

Table of Contents:

Appendix Figure S1……………………………………………Page 2
Appendix Figure S2……………………………………………Page 3
Appendix Figure S3……………………………………………Page 4
Appendix Figure S4……………………………………………Page 5
Appendix Table S1………………………………………………Page 6
Appendix Table S2………………………………………………Page 7
Appendix Table S3………………………………………………Page 9
Appendix Table S4………………………………………………Page 10

Appendix Figure S1

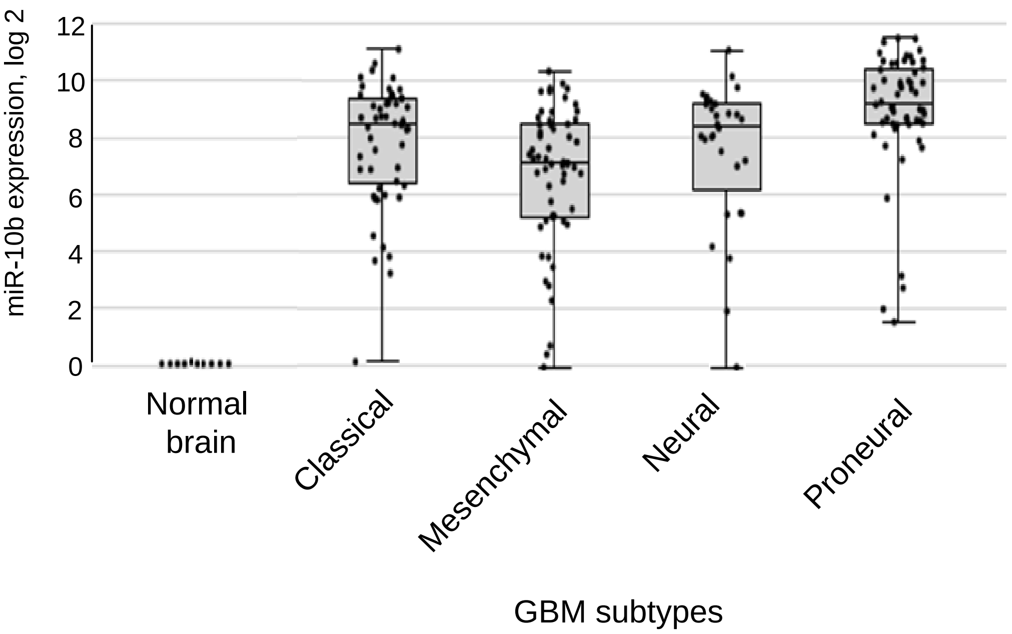

MiR-10b expression in GBM of different subtypes. Expression data and subtype annotation were downloaded from The Cancer Genome Atlas (TCGA) portal. Error bars indicate standard deviation (SD), and grey boxes – standard error of mean (SE).

Appendix Figure S2

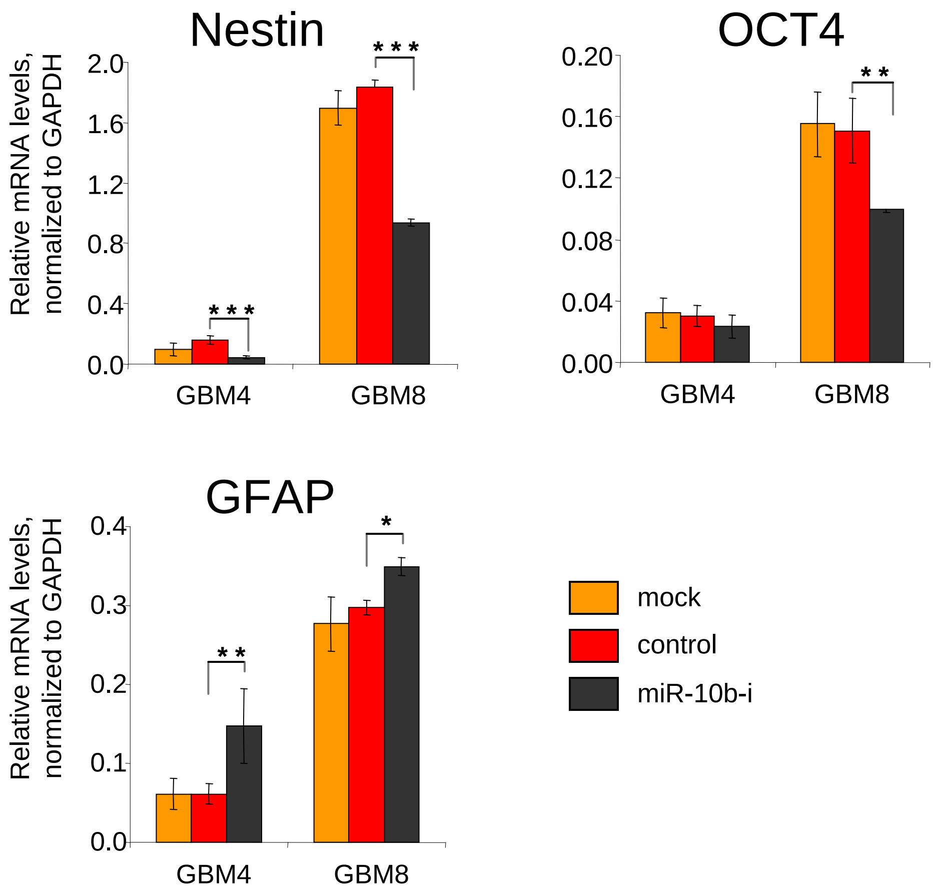

The effect of miR-10b inhibition on stemness and differentiation of GSCs. GSC GBM4 and GBM8 cultures were plated in differentiation conditions as described and transfected with miR-10b inhibitor (miR-10b-i) or scramble control oligonucleotide. The expression of stemness markers (Nestin, OCT4) and astrocytic marker (GFAP) were determined by qRT-PCR analysis. Statistical significance of the differences was determined by Student's t test, with p-values < 0.05 indicated by asterisks, p < 0.01 by two asterisks, and p< 0.001 by three asterisks.

Appendix Figure S3

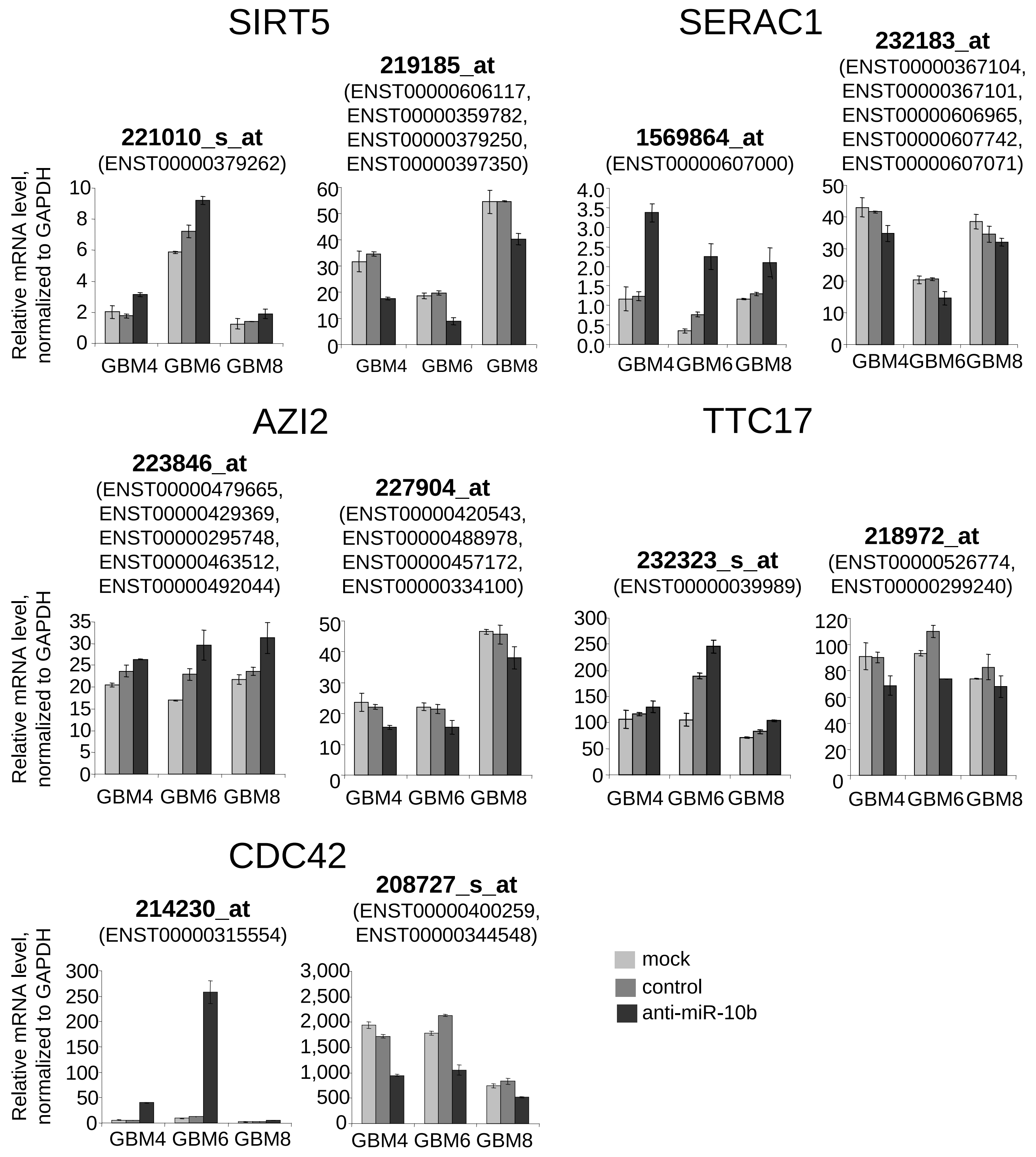

MiR-10b inhibition regulates alternative splicing in GSC. The reciprocal regulation of alternative splice variants was validated for a subset of genes by qRT-PCR analysis. The qRT-PCR primers were designed that each pair of primers amplifies only one of two alternative splice isoforms (each may correspond to one or more transcriptional variants in ENSEMBL). Affymetrix prob set IDs are indicated in bold, and ENSEMBL transcript IDs are included in parenthesis.

Appendix Figure S4

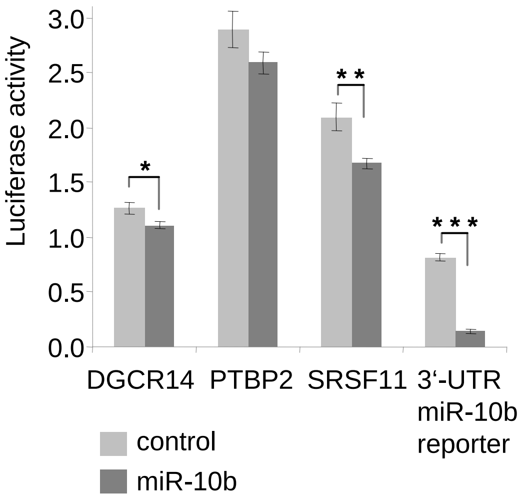

miR-10b regulates expression of several splicing factors through their 3’ UTRs . The effect of miR-10b mimic on candidate splicing factor 3’ UTRs was detected by luciferase reporters’ activity. Statistical significance of the differences was determined by Student's t test, with p-values < 0.05 indicated by asterisks, p < 0.01 by two asterisks, and p< 0.001 by three asterisks.

Appendix Table S1. Correlation between miR-10b and selected splicing factors expression in TCGA GBM dataset.

GENE	R	p-value
**MBNL1**	**-0.16783**	0.005885
**MBNL2**	**-0.20992**	0.000542
**MBNL3**	-0.00052	0.993298
**SART3**	0.374114	2.50E-10
**RSRC1**	0.166311	0.006354
**SRSF11**	0.287724	0.000001
**PTBP2**	0.202025.	0.00088
**DGCR14**	**-0.17725**	0.003599

Appendix Table S2. Primers used in qRT-PCR reaction, 5’→ 3’.

**MBNL1**	F	GCGGGCATCTTGGAATCATG
	R	GCAAGCAAGGCAAAAGCAGA
**MBNL2**	F	TCATGATGCGGTGGAGAAGC
	R	TCCCAAGCTGCAGTTTTCCA
**MBNL3**	F	GAAAATGGTCGTGTGGTGGC
	R	TCCGCCCATTAATCTCCAGC
**SART3**	F	TGAGAGACTGGAGGAGCAGT
	R	TCACCTTGGTAAGCTCCCCT
**RSRC1**	F	CTCCCAGGTTCACGCCATTT
	R	GGTGCCTGTAGTCCCAGCT
**SRSF11**	F	TGCGGACTCTCTTCGGTTTC
	R	CAGATGCTGTGCCACAACTG
**BTBP2**	F	CAGTTGGCGTGAAGAGAGGA
	R	AGTACACGAGAAGGAGCACC
**DGCR14**	F	AGCCGAGGAGAATGGAGACT
	R	TTCTCCTCCTCCTCTCCAGC
**GAPDH**	F	ATGTTCGTCATGGGTGTGAA
	R	TGTGGTCATGAGTCCTTCCA
**CDC42** ENST00000315554	F	GCCTATCACTCCAGAGACTGC
	R	GCTCGAGGGCAGCTAGGATA
**CDC42** ENST00000400259, ENST00000344548	F	GCCTATCACTCCAGAGACTGC
	R	GCTCCAGGGCAGCCAATATT
**SIRT5** ENST00000379262	F	TTTAACACGGAGACCACCCC
	R	ACATGAAGACACAGCCACCA
**SIRT5** ENST00000606117, ENST00000359782, ENST00000379250, ENST00000397350	F	TGTACCCAGCAGCCATGTTT
	R	GGAAGAGTCGTTCCACAGGG
**SERAC1** ENST00000607000	F	ACTGCGGAATCCATTTGCTG
	R	AGCAATCAAGAGCCAGCTGA

Appendix Table S2. Primers used in qRT-PCR reaction, 5'→ 3'.

**SERAC1** ENST00000367104, ENST00000367101, ENST00000606965, ENST00000607742, ENST00000607071	F	ACCGACTTCACAAGGACTGC
	R	CAGCGTGTGAGGACTCCATA
**AZI2** ENST00000479665, ENST00000429369, ENST00000295748, ENST00000463512, ENST00000492044	F	ACCGGAAACCACTGATTCCA
	R	TGGGTAGGCTGCGAATGTAC
**AZI2** ENST00000420543, ENST00000488978, ENST00000457172ENST00000334100	F	ACAAGAGCTGGAACTGATGA GG
	R	ATCCCATGATTGCGCCACT
**TTC17** ENST00000039989	F	AGCCTGAAGTTGATCCGCTG
	R	CACCGTACCACTGCAAACAG
**TTC17** ENST00000526774, ENST00000299240	F	CAGTCACTGGATGCTGCTGA
	R	ATTGCTCTCCTCAACCACCG

Appendix Table S3. Primers used for cloning 5'-UTR and 3'-UTR reporters, 5'→ 3'.

MBNL1	F	CATAGATCTTGCCTTTCCTGACGTCTCTG
	R	CATCCATGGTCTCCCACTAGAAATTCACAGCA
MBNL2	F	CATAGATCTCAGACGGCTTTCAGAGTACA
	R	CATCCATGGCTTGTTTAGCTCTGCCTGTCC
MBNL3	F	CATAGATCTAGTCCACAGTAATATTGTCCT
	R	CATCCATGGGCAAAATTAAAATCCAATGTACC
SART3	F	CATAGATCTGGAACGCATGCCATTATAAT
	R	CATCCATGGCTTGCGCTTCTAATGACTCT
RSRC1	F	CATAGATCTTGCACTCGATATCTCGGCTG
	R	CATCCATGGTTCTGGGTCCAGACCTTTGG
SRSF11	F	CTATCTCGAGCCTGCATCAGTGTCATTCCT
	R	CATGCGGCCGCATCCAACCCCATTCCCACAC
BTBP2	F	ATGTCTCGAGTGGGAAGATGAAGATTGGGG
	R	TAAGCGGCCGCACTGCAGCAAGAGAGCATGG
DGCR14	F	ATGTCTCGAGCAGCTACACACCATCCCCAG
	R	TAAGCGGCCGCAGCACAGGACAGATGCTCAG

Appendix Table S4: Number of replicates (n) and P-values for Figures 1-9.

Figure	Panel	Data	n, miR-10b-i or miR-10b OV	n, control	P-value
1	A	GBM4	7	7	0.0000129750500711494
1	A	GBM6	7	7	0.0000176021691488482
1	A	GBM8	7	7	0.000111720263628652
1	B	# of colonies per well	7	7	0.0000023104510132783
1	B	Average diameter	7	7	0.0000107390282011561
1	B	Total diameter	7	7	0.0000000713501881450
4	B	MBNL1, GBM4	4	4	0.0007995071296076130
4	B	MBNL1, GBM6	4	4	0.0002379337301875310
4	B	MBNL1, GBM8	4	4	0.0118740070966773000
4	B	MBNL1, BT74	4	4	0.0038204754850762100
4	B	MBNL1, LN215	4	4	0.0092288957641396900
4	B	MBNL1, U251	4	4	0.0194076738512395000
4	B	MBNL2, GBM4	4	4	0.0003346400370221660
4	B	MBNL2, GBM6	4	4	0.0000584658408152867
4	B	MBNL2, GBM8	4	4	0.0004020170822921720
4	B	MBNL2, BT74	4	4	0.0005110293850547590
4	B	MBNL2, LN215	4	4	0.0000005424661282705
4	B	MBNL2, U251	4	4	0.0030148802717931100
4	B	MBNL3, GBM4	4	4	0.0078631199956037300
4	B	MBNL3, GBM6	4	4	0.0000330092051912746
4	B	MBNL3, GBM8	4	4	0.0025370119659806200
4	B	MBNL3, BT74	4	4	0.0007468202281636000
4	B	MBNL3, LN215	4	4	0.0000598870568697445
4	B	MBNL3, U251	4	4	0.0001729523412431360
4	B	SART3, GBM4	4	4	0.0005164367845712140
4	B	SART3, GBM6	4	4	0.0006467601152727780
4	B	SART3, GBM8	4	4	0.0009426750255806480
4	B	SART3, BT74	4	4	0.0002957383826863870

Appendix Table S4: Number of replicates (n) and P-values for Figures 1-9 (continued).

4	B	SART3, LN215	4	4	0.0000694230389902142
4	B	SART3, U251	4	4	0.0053989922059124900
4	B	RSRC1, GBM4	4	4	0.0090996428081293600
4	B	RSRC1, GBM6	4	4	0.0018446766935936000
4	B	RSRC1, GBM8	4	4	0.0003252524501075080
4	B	RSRC1, BT74	4	4	0.0030602646776663200
4	B	RSRC1, LN215	4	4	0.0027249212490895700
4	B	RSRC1, U251	4	4	0.2335944191861290000
4	B	SRSF11, GBM4	4	4	0.0262393917315574000
4	B	SRSF11, GBM6	4	4	0.0000124525994868730
4	B	SRSF11, GBM8	4	4	0.0001284958894357900
4	B	SRSF11, BT74	4	4	0.0006542181658640980
4	B	SRSF11, LN215	4	4	0.0000157168556866047
4	B	SRSF11, U251	4	4	0.0007307563453193610
4	B	PTBP2, GBM4	4	4	0.0130523161250069000
4	B	PTBP2, GBM6	4	4	0.0003770302570259140
4	B	PTBP2, GBM8	4	4	0.0002437842581125920
4	B	PTBP2, BT74	4	4	0.2400155496207370000
4	B	PTBP2, LN215	4	4	0.0010324874457962700
4	B	PTBP2, U251	4	4	0.0115053061236360000
4	B	DGCR14, GBM4	4	4	0.0160212407165258000
4	B	DGCR14, GBM6	4	4	0.0006386739232655790
4	B	DGCR14, GBM8	4	4	0.0001475708067200030
4	B	DGCR14, BT74	4	4	0.0004350960236675040
4	B	DGCR14, LN215	4	4	0.0000037035250142865
4	B	DGCR14, U251	4	4	0.0013554059163899700
4	D	miR-10b reporter	4	4	0.0000286555067001374
4	E	MBNL2 WT	5	5	0.0110965099319385000
4	E	MBNL3 WT	5	5	0.0078906149358723900
4	E	SART3 WT	5	5	0.0059394300992582200

Appendix Table S4: Number of replicates (n) and P-values for Figures 1-9 (continued).

4	E	SART3 MT	5	5	0.0019045648747851300
5	B	miR-10b level	10	8	0.0000001246759668869
5	C	MBNL1	10	8	0.0000040940894834540
5	C	MBNL2	10	8	0.0000000006206988817
5	C	MBNL3	10	8	0.1302253058925780000
5	C	SART3	10	8	0.0000000029978234278
5	C	RSRC1	10	8	0.0000005969445856523
5	C	PTBP2	10	8	0.0036553437829379800
5	C	DGCR14	10	8	0.0000672575133379702
5	C	SRSF11	10	8	0.0000000049424632469
5	E	Imaging signal ratio	7	7	0.0216789103576584000
5	F	Signal ratio d 30/ d5	7	6	0.0232766962780480000
6	E	miR-10b level	21	28	0.0042978143372822100
6	F	MBNL1	21	28	0.0000239311182410363
6	F	MBNL2	21	28	0.0000000234977398992
6	F	MBNL3	21	28	0.0000828817669839393
6	F	SART3	21	28	0.0664377568631427000
6	F	RSRC1	21	28	0.0072398478876506700
6	F	PTBP2	21	28	0.0404010772610865000
6	F	DGCR14	21	28	0.0904140416175864000
6	F	SRSF11	21	28	0.0017053676747766300
8	F	PCNA	4	4	0.0036740683384316300
8	F	KI67	4	4	0.0968294260890747000
8	F	Cleaved Caspase 3	4	4	0.0479069227622826000
8	F	Migrating cells	4	4	0.2226696625331730000
9	D	Signal ratio	4	4	0.0474568790158070000
9	E	miR-10b level	10	10	0.0000145064489387411
9	F	P21 level	4	4	0.0000266250883946358
