# Supplementary material for: Therapeutic potential of targeting microRNA‐10b in established intracranial glioblastoma: first steps toward the clinic
Source: EMBO Mol Med. 2016 Feb 10;8(3):268–87. doi: 10.15252/emmm.201505495 (PMC4772951; doi:10.15252/emmm.201505495)
Supplement: Supplementary file 2 — Expanded View Figures PDF [file EMMM-8-268-s002.pdf]

Expanded View Figures

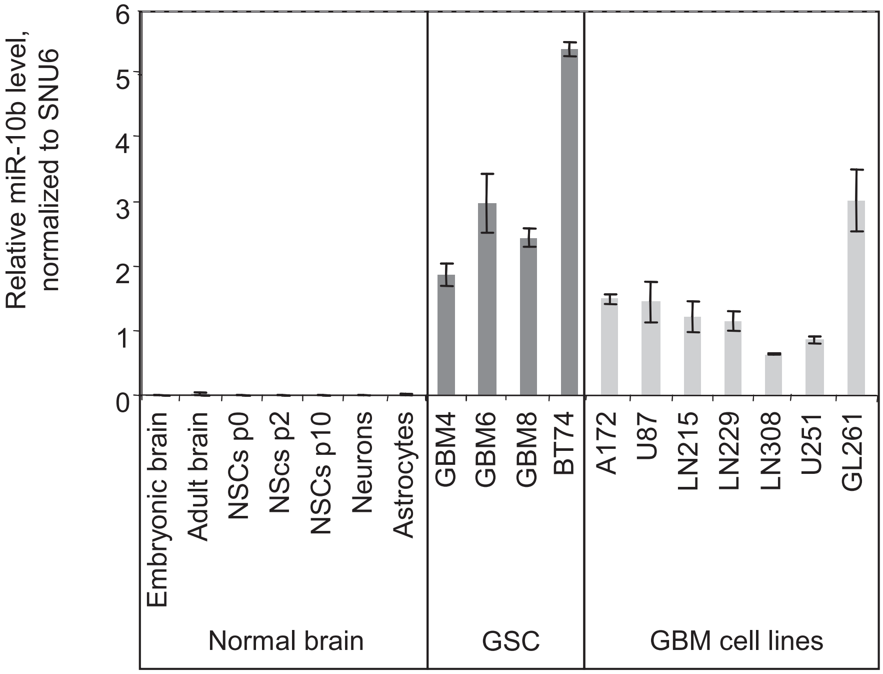

Figure EV1. miR-10b expression in GBM stem-like cells (GSC), relative to human GBM cell lines, normal human brain tissues, and brain cells.
The relative expression levels of miR-10b have been assessed by qRT–PCR analysis and normalized to levels of snRNA U6. NSCs—primary normal embryonic neural stem cells of the indicated passages.

Source data are available online for this figure.

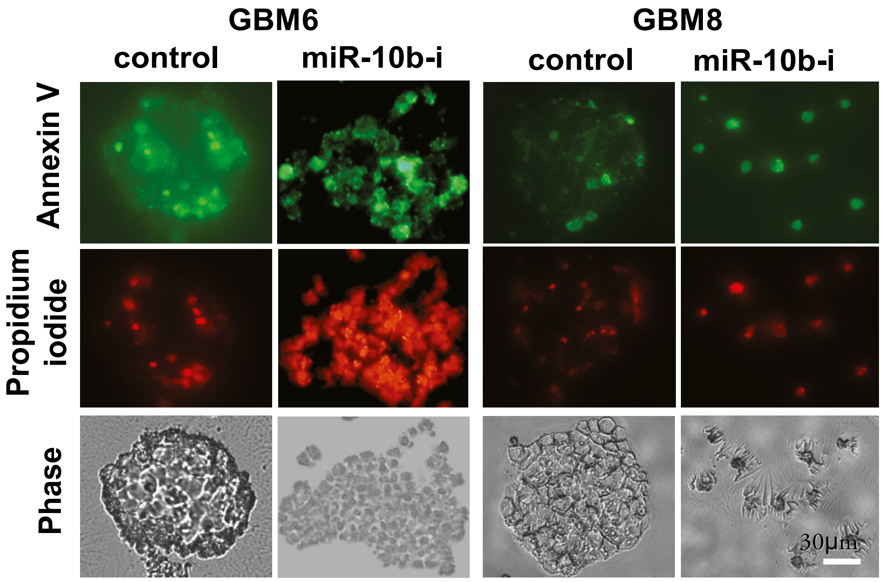

Figure EV2. Annexin V and propidium iodide staining of GSC at day 5 after miR-10b inhibition.
GSC neurospheres were dissociated to single cell suspension and transfected with either miR-10b inhibitor (labeled "miR-10b-i") or non-targeting control oligonucleotide. Cells were stained with Annexin V and propidium iodide at day 5 after transfections. Cells were collected on microscopic slides by Cytospin, and fluorescence images were taken immediately after staining.

Source data are available online for this figure.

© 2016 The Authors

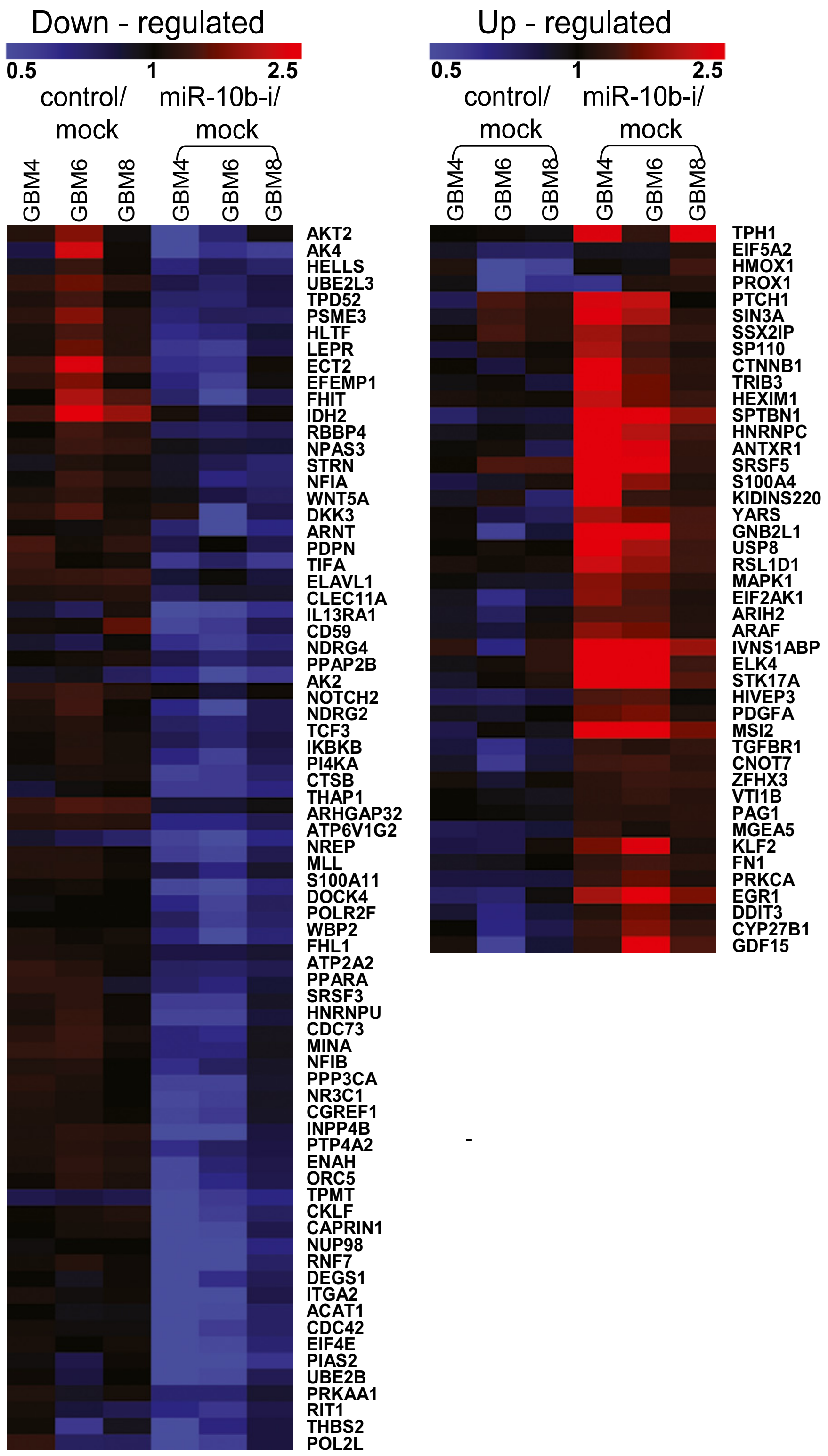

Figure EV3. Regulation of cell cycle-related genes by miR-10b in GSC.

Change in the expression of genes related to "cell cycle" bioterm upon miR-10b inhibition in GSC, as determined by the whole-genome expression profiling. The cell cycle-associated genes were selected using the Ingenuity Pathway Analysis. "miR-10b-i" indicates the treatment with miR-10b inhibitor.

© 2016 The Authors

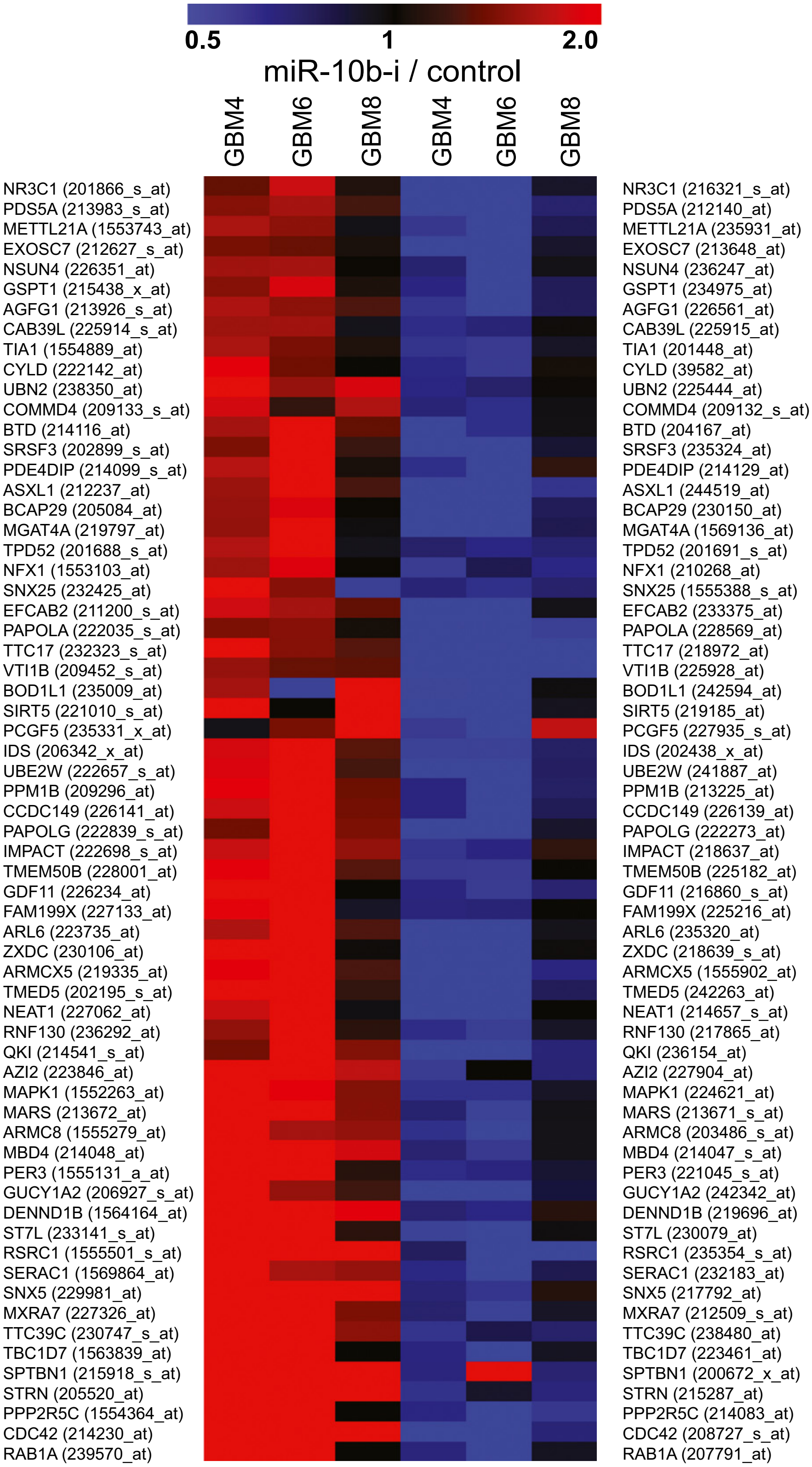

Figure EV4. Regulation of splice isoforms by miR-10b inhibition in GSC, as determined by the whole-genome expression profiling.

Each row of the heatmap demonstrates a pair of probe sets corresponding to different splice isoforms of the same gene, and regulated inversely (with fold change > 1.2 on a left side, and < 0.8 on a right side, and $P < 0.05$ in at least two out of three GSC cultures). Affymetrix prob set IDs are indicated in parenthesis.

© 2016 The Authors

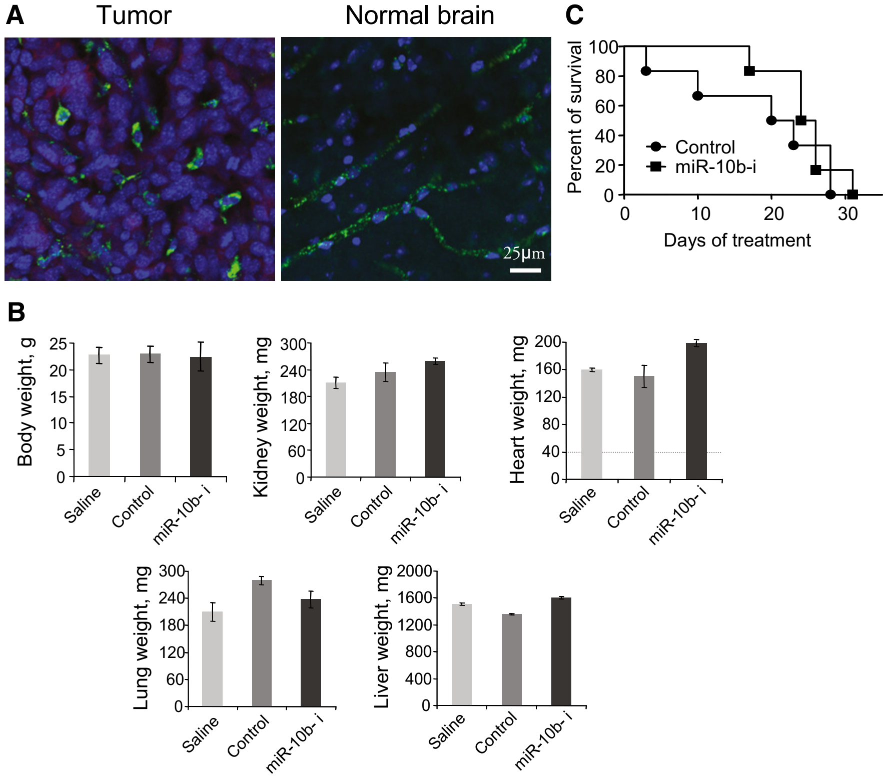

Figure EV5. Systemic treatment of orthotopic GL261 tumors with miR-10b inhibitor.

A Systemic delivery of miR-10b ASO to GL261 allograft tumors. About 100 mg/kg of uncomplexed 2′-O-MOE-PS miR-10b inhibitor was injected subcutaneously daily to BLACK6 mice bearing orthotopic GL261 tumors. Tumors and normal brain sections are shown. The staining for ASO is in green, DAPI nuclear staining in blue, and MCherry fluorescence in red.

B Treatment with miR-10b inhibitor does not affect body and organ weight of the mice. Mice were treated daily with miR-10b ASO (miR-10b-i) or non-specific control oligonucleotide at 100 mg/kg, or saline, for 14 days, by subcutaneous injections. No difference in animal weight or weight of the organs was observed between the treatment and control groups. The error bars represent Standard Deviation within each group of mice, $N = 7$ mice per group.

C The effect of systemic treatment on viability of GL261-bearing mice. miR-10b ASO or non-targeting control of the same chemistry was injected subcutaneously to the mice bearing orthotopic GL261 tumors at 100 mg/kg daily for 30 days. The mice were sacrificed upon developing lethargy or losing more than 30% of body weight, and Kaplan–Meier survival plots were built accordingly.

Source data are available online for this figure.

 © 2016 The Authors
