# Supplementary material for: Therapeutic potential of targeting microRNA‐10b in established intracranial glioblastoma: first steps toward the clinic
Source: EMBO Mol Med. 2016 Feb 10;8(3):268–87. doi: 10.15252/emmm.201505495 (PMC4772951; doi:10.15252/emmm.201505495)
Supplement: Supplementary file 5 — Source Data for Expanded View and Appendix [file EMMM-8-268-s014.zip › emmm201505495-sup-0016-SDataFigEV5.pdf]

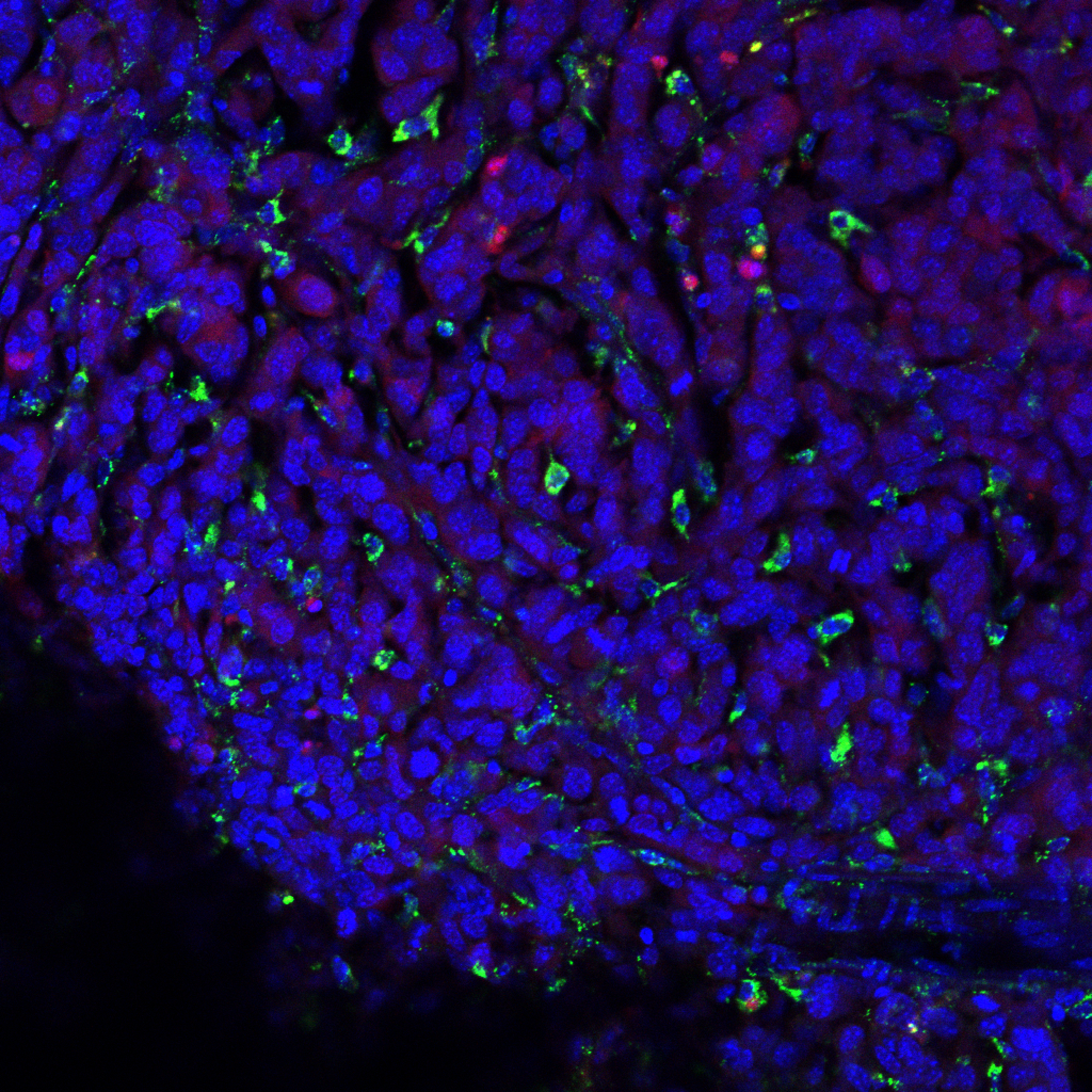

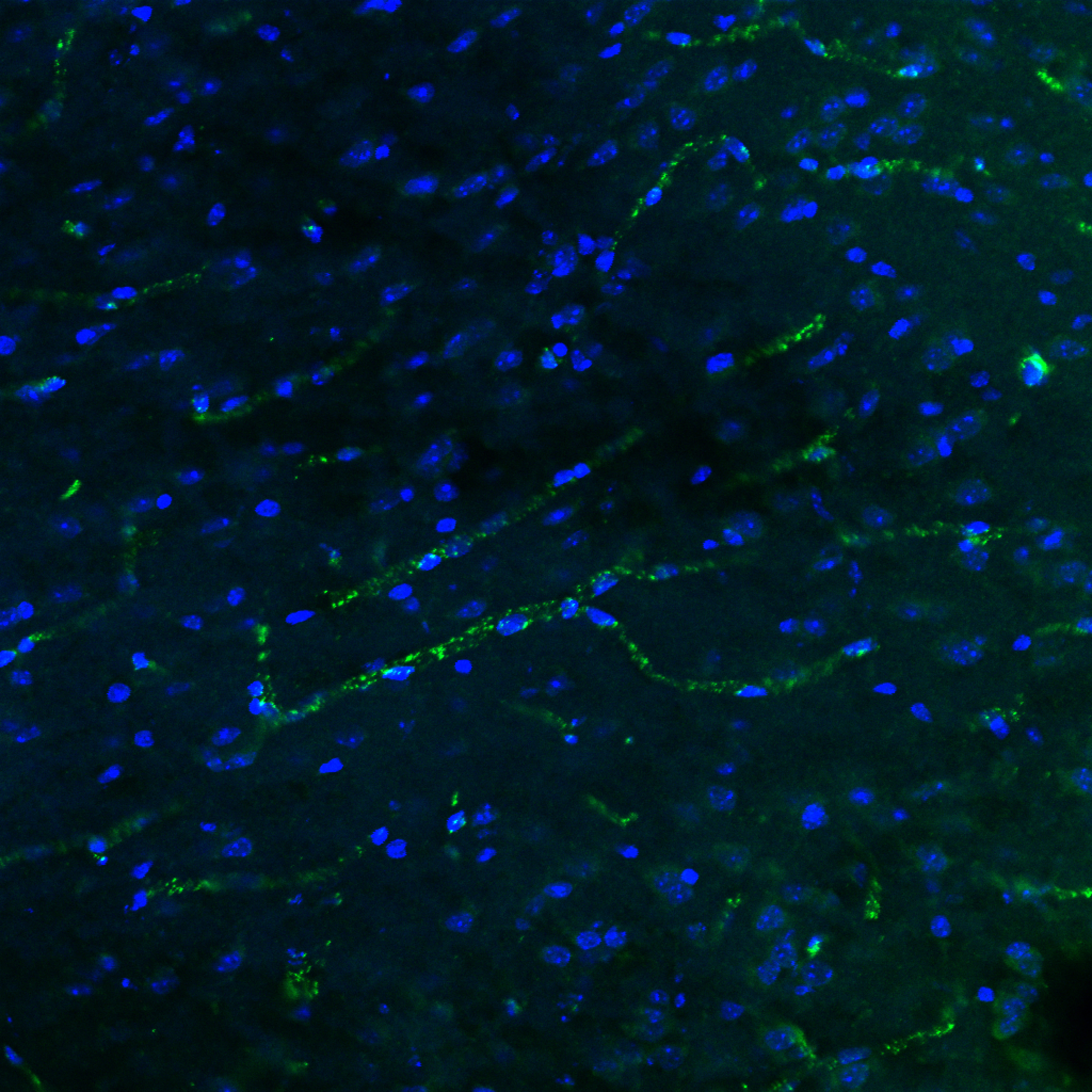

Treatment	23.1	
	24.9	
	21.8	
	22.7	
	23.4	
	16.9	
	24.5	
	22.5	2.671
Control	23.1	
	23.4	
	21.4	
	21.5	
	23.2	
	22.6	
	25	
	22.9	1.231
Saline	22.8	
	21.2	
	23.1	
	25.1	
	21.1	
	22.9	
	22.7	1.468

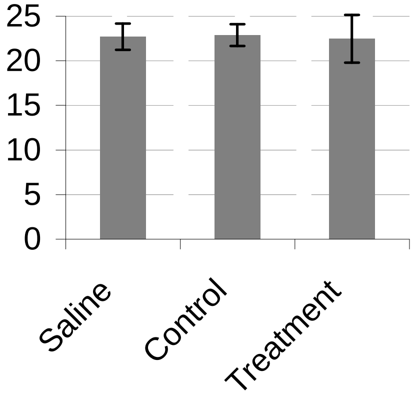

	kidney	heart	lung	liver
53 NS	210	160	210	1508
55 C	235	150	280	1360
54 T	259	199	237	1600

Kidney

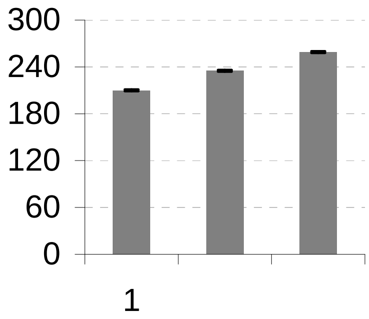

Heart

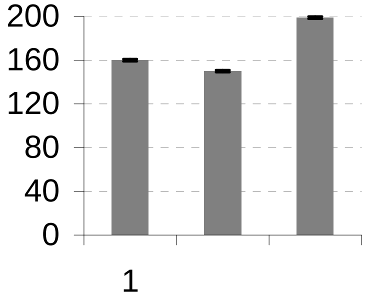

Lung

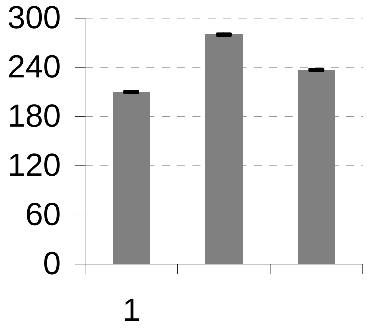

Liver

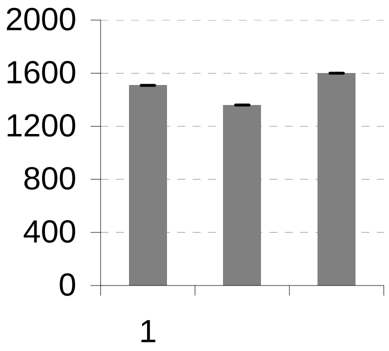

Day	Control	miR-10b-i
3	1	
10	1	
17		1
20	1	
23	1	
24		1
24		1
26		1
26		1
28	1	
28	1	
31		1
