# Supplementary material for: Therapeutic potential of targeting microRNA‐10b in established intracranial glioblastoma: first steps toward the clinic
Source: EMBO Mol Med. 2016 Feb 10;8(3):268–87. doi: 10.15252/emmm.201505495 (PMC4772951; doi:10.15252/emmm.201505495)

	10b		U6		normalized to U6			
EB	Undetermined	0	18.651	2.42935E-06	0		0.000	0
EB	Undetermined	0	18.087	3.59146E-06	0	SE		0
AB	Undetermined	0	23.989	6.00608E-08	0		0.000	0
AB	Undetermined	0	23.911	6.33975E-08	0	SE		0
NSC p0	Undetermined	0	19.884	1.03382E-06	0		0.000	0
NSC p0	Undetermined	0	19.812	1.08641E-06	0	SE		0
NSC p1	Undetermined	0	20.794	5.50025E-07	0		0.000	0
NSC p1	Undetermined	0	20.781	5.55003E-07	0	SE		0
NSC p2	Undetermined	0	21.186	4.19158E-07	0		0.000	0
NSC p2	Undetermined	0	20.780	5.55388E-07	0	SE		0
neurones	Undetermined	0	17.124	7.00103E-06	0		0.000	0
neurones	Undetermined	0	17.008	7.58721E-06	0	SE		0
astrocytes	Undetermined	0	21.384	3.65405E-07	0		0.000	0
astrocytes	Undetermined	0	20.961	4.89903E-07	0	SE		0
GBM4	25.506	2.0986E-08	19.887	1.03138E-06	2.034755		1.862	0.244743436
GBM4	25.689	1.84859E-08	19.801	1.09472E-06	1.688635	SE		0.173059743
GBM6	25.108	2.76528E-08	20.236	8.09761E-07	3.414931		2.959	0.644589457
GBM6	25.169	2.65079E-08	19.849	1.0589E-06	2.503344	SE		0.455793576
GBM8	24.938	3.1111E-08	19.529	1.32186E-06	2.353579		2.468	0.161980969
GBM8	25.120	2.74237E-08	19.845	1.06184E-06	2.582655	SE		0.114537842
BT74	24.123	5.47336E-08	19.869	1.04432E-06	5.241052		5.350	0.154700502
BT74	23.696	7.35857E-08	19.501	1.34777E-06	5.459832	SE		0.109389774
A172	26.967	7.62297E-09	20.961	4.89903E-07	1.556017		1.485	0.101062818
A172	26.930	7.821E-09	20.785	5.53467E-07	1.413092	SE		0.071462204
U87	27.439	5.49589E-09	21.600	3.14595E-07	1.746973		1.441	0.43319526
U87	27.668	4.68924E-09	21.206	4.13388E-07	1.134342	SE		0.306315306
LN215	27.028	7.30737E-09	20.917	5.05075E-07	1.446791		1.210	0.335333096
LN215	27.694	4.60548E-09	21.010	4.73543E-07	0.972558	SE		0.237116306
LN229	28.920	1.96885E-09	22.656	1.51309E-07	1.301212		1.154	0.208635274
LN229	28.947	1.93234E-09	22.312	1.92052E-07	1.006157	SE		0.147527417
LN308	32.052	2.24588E-10	21.633	3.07481E-07	0.073041		0.063	0.013655589
LN308	32.299	1.89248E-10	21.437	3.52225E-07	0.053729	SE		0.00965596
U251	29.789	1.078E-09	22.457	1.73688E-07	0.620652		0.569	0.072628902
U251	30.018	9.19775E-10	22.425	1.77583E-07	0.51794	SE		0.051356389
GL261	25.789	1.7248E-08	20.538	6.56819E-07	2.625982		2.963	0.477233258
GL261	25.839	1.66604E-08	20.918	5.04725E-07	3.300892	SE		0.337454873

	normalized to U6	
Embr. Brai	0	0
Adult brain	0	0
NSCs p0	0	0
NScs p1	0	0
NSCs p2	0	0
Neurones	0	0
Astrocytes	0	0
GBM4	1.861694845	0.173059743
GBM6	2.959137473	0.455793576
GBM8	2.468116671	0.114537842
BT74	5.35044198	0.109389774
A172	1.484554533	0.071462204
U87	1.440657796	0.306315306
LN215	1.209674293	0.237116306
LN229	1.15368478	0.147527417
LN308	0.633854021	0.00965596
U251	0.869295991	0.051356389
GL261 (mo	2.963437043	0.337454873

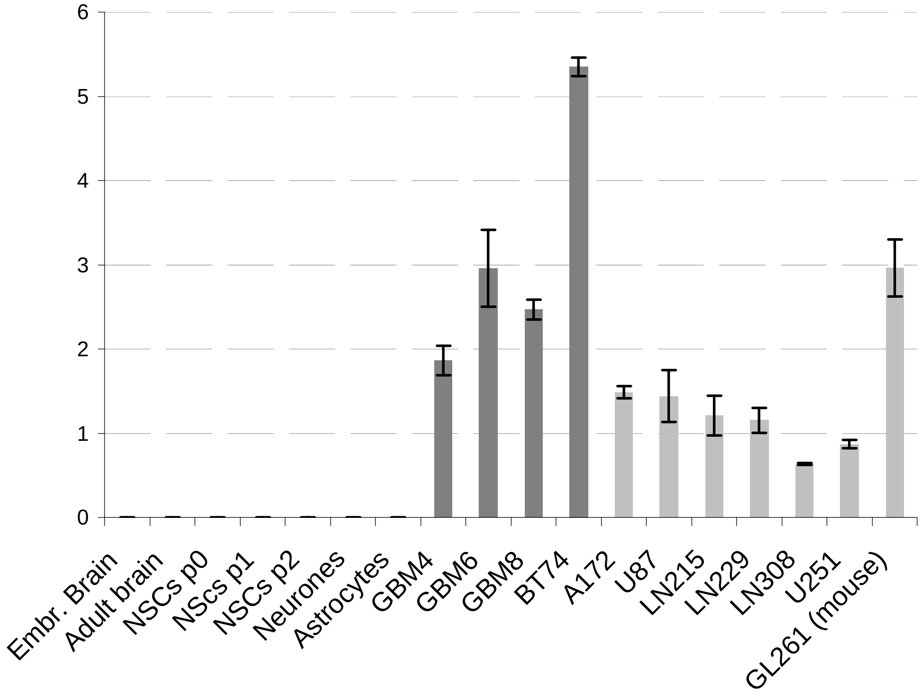

Supplement: Supplementary file 5 — Source Data for Expanded View and Appendix [file EMMM-8-268-s014.zip › emmm201505495-sup-0014-SDataFigEV1.pdf]
