# Supplementary figures and images for: Therapeutic potential of targeting microRNA‐10b in established intracranial glioblastoma: first steps toward the clinic
Source: EMBO Mol Med. 2016 Feb 10;8(3):268–87. doi: 10.15252/emmm.201505495 (PMC4772951; doi:10.15252/emmm.201505495)

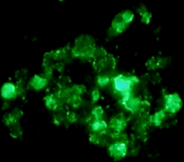



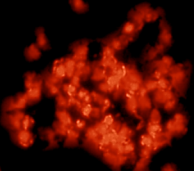









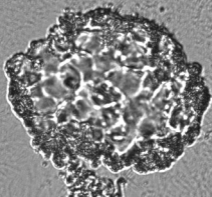

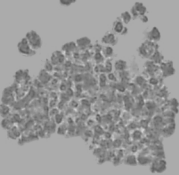

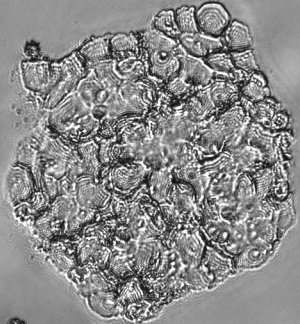

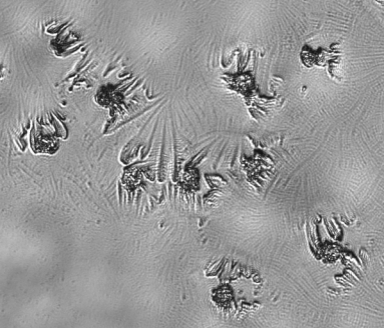

Supplement: Supplementary file 5 — Source Data for Expanded View and Appendix [file EMMM-8-268-s014.zip › emmm201505495-sup-0015-SDataFigEV2.pdf]
