# Supplementary material for: Therapeutic potential of targeting microRNA‐10b in established intracranial glioblastoma: first steps toward the clinic
Source: EMBO Mol Med. 2016 Feb 10;8(3):268–87. doi: 10.15252/emmm.201505495 (PMC4772951; doi:10.15252/emmm.201505495)
Supplement: Supplementary file 7 — Source Data for Figure 1 [file EMMM-8-268-s005.pdf]

Figure 1 Panel A Source Data

GBM4

Mock	Control	miR-10b-i
182631	133510	48954
130973	174978	45087
164790	108457	38146
110941	105456	40778
113664	89247	37548
106261	91937	36570
112984	108847	41799

GBM6

Mock	Control	miR-10b-i
795221	579599	436034
690743	538976	232400
559198	604573	316055
519287	658120	290409
613637	643332	267192
602802	628658	434665

GBM8

Mock	control	miR-10b-i
609999	422196	148034
569997	248144	163826
693854	248448	119682
503555	311496	103708
340981	356045	112791
543677	251869	150860

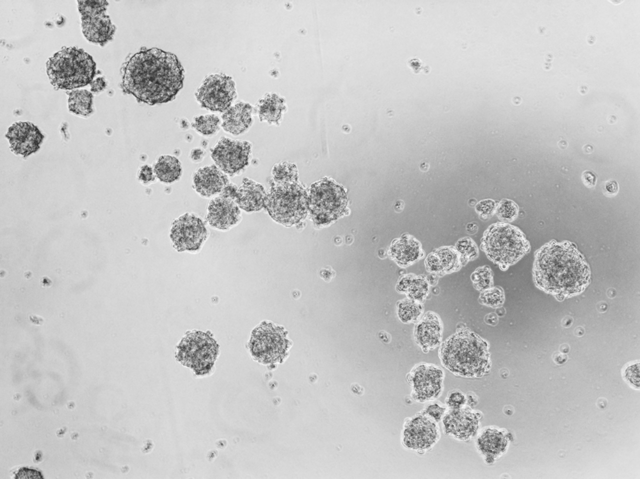

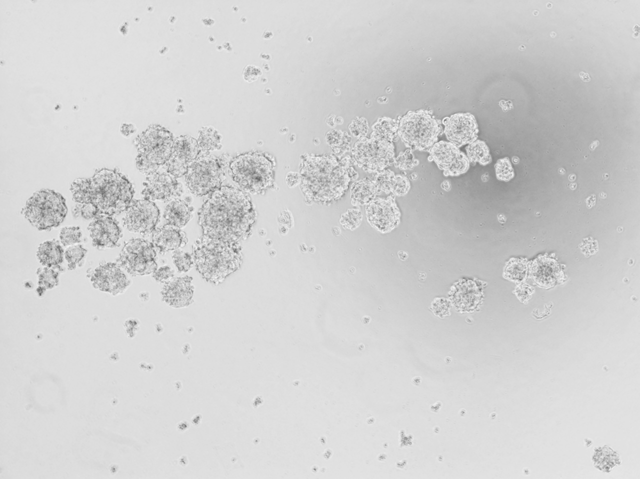

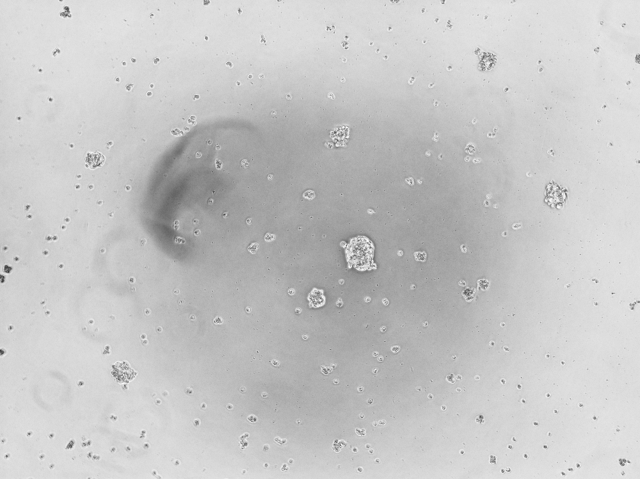

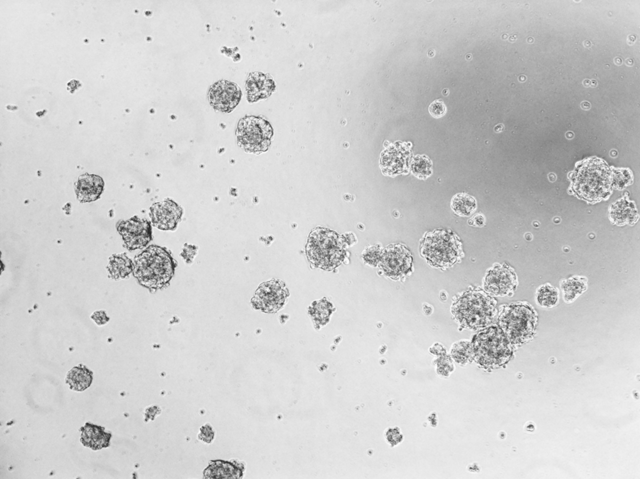

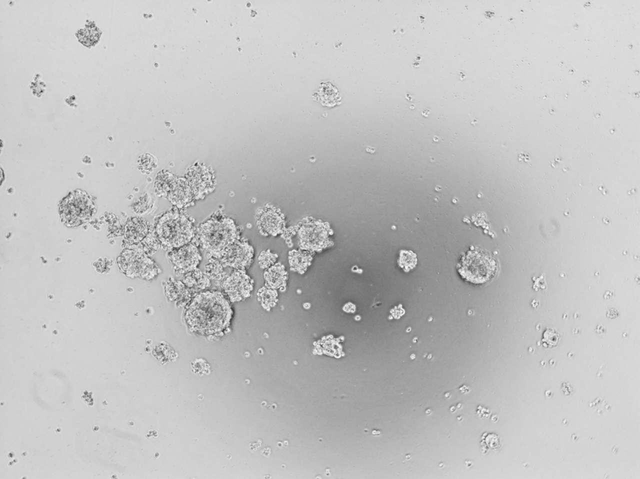

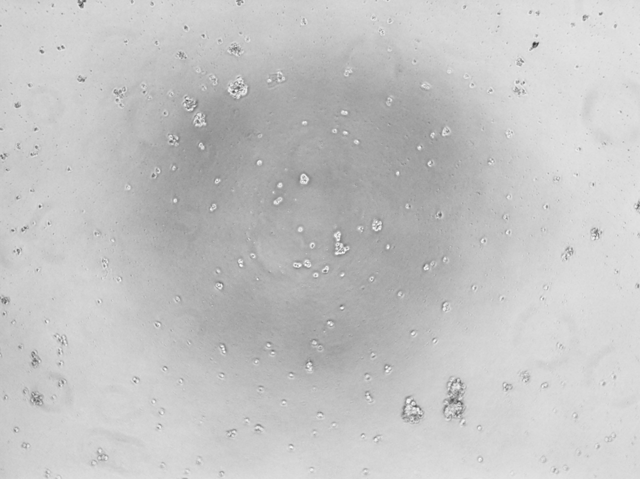

Fugure 1B Source Data 7

Distance	pixel					
control 1	control 2	control 3	control 4	control 5	control 6	control 7
361.9861	136.1516	62.89632	157.3096	110.9748	188.7655	153.6886
153.0615	107.1461	125.7926	208.9838	141.4083	124.0591	137.6312
238.5712	170.9011	174.9516	145.1865	83.67983	155.0913	68.81559
113.3118	237.1067	159.1346	213.63	152.8408	148.9659	235.7043
101.0015	148.8294	159.1346	201.9571	166.5814	91.98113	133.4773
135.2479	106.8305	210.1893	136.0486	167.3597	70.67861	224.9541
122.2709	115.3413	193.9339	161.5657	246.2784	90.63711	226.0106
105.5986	198.7603	114.8743	201.5277	167.3597	159.6329	146.769
115.1594	69.97143	157.08	208.2918	258.8266	183.9623	134.7691
177.9303	88.69818	243.9517	148.3832	211.1488	107.539	195.4172
237.2404	152.5269	161.4316	296.7665	202.3499	93.8458	249.9733
147.3233	120.9151	165.5381	142.6282	110.9748	199.4755	230.0313
147.3233	140.5448	124.4657	137.6836	200.234	151.3218	289.3769
113.931	295.2098	307.5884	183.3727	143.0325	205.9567	227.7606
104.9303	145.2703	166.8413	205.9567	103.3632	174.0414	130.8556
88.96516	168.5135	118.9496	61.85472	80.78285	185.5253	146.1776
197.5125	116.9403	208.9838	73.77693	128.9663	240.3145	124.2335
212.36	166.1933	201.9571	96.87386	145.1865	106.4596	137.6836
257.5042	232.2144	275.9434	54.4035	178.0599	47.61017	190.4407
151.908	185.3093	153.171	83.33405	166.5814	231.3144	156.6659
168.591	85.79559	229.5601	94.38273	204.2673	185.8751	194.12
211.1973	182.3706	159.8136	209.8456	230.2822	116	219.6282
184.2436	182.4632	239.8936	116.3107	204.0905	150.7004	135.3037
271.6636	166.1933	145.0871	227.7606	159.6329	71.6927	158.0422
169.6311	166.1933	81.58314	126.3081	142.6282	70.67861	367.0602
127.3442	226.9194	326.863	188.3826	142.4256	73.77693	294.5691
237.0179	241.8303	74.94191	207.7365	209.8112	349.5936	329.8967
138.4608	179.0069	70.67861	201.9571	305.0432	151.9882	296.1334
156.5832	244.3997	134.9297	102.6624	253.1015	153.171	264.6189
253.8583	266.0945		99.44781	129.0782	143.0325	176.308
253.8583	151.5274		269.1361	247.7979	88.70502	169.6729
139.5994	161.4527		132.9897	114.8743	181.6323	230.0313
156.6955	153.4098		139.5066	221.9496	288.8776	104.3363
230.7003	220.8873		203.4174	139.8168	135.9424	118.9496
289.5889	177.8716		262.6199	163.4315	183.4121	231.408
139.5994	179.0069		224.9541	269.8594	182.4254	178.6266
217.7578	211.2771		226.7759	198.1684	207.8755	177.125
223.8122	141.86		234.0137	126.3081	161.2526	197.1824
204.8561	132.6342		234.0137	163.564	161.4316	127.446
211.9455	102.8037		141.0505	118.9496		193.9339
202.8718			250.4925	62.3198		105.5744
166.1742			176.7986			261.7112
98.71169			105.3691			209.26
198.2236			86.81308			209.26
			185.2917			304.2138
			301.1611			181.95
			188.9948			151.703

			129.9699			205.9567
						125.85
						127.7289
						124.4657
						149.691
						275.9172
7936.123	6677.372	4950.16	8187.697	6973.39	6015.241	10138.11
7268.299						
1678.65						
N of spheres						
44	42	29	48	41	39	53
42.28571						
7.521398						
Average diameter						
180.3664	166.9343	170.6952	170.577	170.0827	154.237	191.2851
172.0254						
11.47823						

Fugure 1B Source Data 7

a-10b 1	a-10b 2	a-10b 3	a-10b 4	a-10b 5	a-10b 6	a-10b 7
144.4388	113.9912	120.9357	131.8996	67.33095	69.85673	73.77693
91.50903	100.3151	180.0355	112.8455	67.9712	56.48668	68.50016
63.2397	110.1915	118.3412	39.12036	173.6262	65.3726	137.053
92.76263	124.6396	91.35111	87.47579	125.7926	240.3145	123.3003
86.64662	98.05891	72.98996	72.98996	120.7565	149.691	131.8996
93.8458	122.5368	67.22365	56.48668	163.2105	104.8195	72.98996
66.4677	86.64662	237.4132	45.75451	67.9712	108.0079	72.59327
147.5539	110.4532	69.75332	43.32331	181.2742	101.175	115.5635
155.0447	72.59327	108.6742	137.053	119.3132	225.755	59.3533
83.67983	110.4532	126.4794	193.7477	102.3102	86.72989	77.96344
182.7022	125.4478	139.1439	176.0211	97.69012	79.06675	91.50903
91.35111	136.2076	51.11981	78.24072	256.3886	44.3118	88.70502
81.31725	83.33405	68.50016	75.99411	84.964	51.11981	80.78285
145.4846	86.64662	78.33293	61.26841	131.6805	66.359	84.36715
86.14528	73.77693	61.85472	47.61017	62.89632	57.87539	73.77693
118.3412	106.4596	64.59499	59.3533	59.10954	49.39617	42.98876
97.09716	80.78285	80.15487	51.68158	67.22365	48.65997	41.1001
86.14528	164.4443	41.1001	57.87539	40.39142	51.11981	72.59327
70.67861	164.9702		36.04717	188.6507	42.482	59.10954
83.67983	83.42063			219.2664	41.1001	56.99558
75.99411	91.50903					33.9856
70.67861	72.98996					
101.175	78.33293					
153.3594	76.93817					
94.99264	65.3726					
156.4814						
114.0545						

2834.867	2540.513	1777.999	1564.788	2397.818	1739.7	1658.907
2073.513						
505.368						
27	25	18	19	20	20	21
21.42857						
3.309438						
104.9951	101.6205	98.77771	82.35728	119.8909	86.98498	78.99559
96.23172						
14.41735						

Fugure 1B Source Data 7

Mock1	Mock2	Mock3	Mock4	Mock5	Mock6
164.9702	151.3218	296.1334	143.083	418.6062	294.5691
164.9702	141.0505	166.5381	202.3499	127.446	174
226.266	152.8408	114.8743	314.0911	136.2076	140.1777
134.4473	315.1008	187.999	110.4532	136.2076	299.3097
233.8903	181.2742	253.5574	134.7691	314.8945	341.3185
211.2855	129.6919	408.1457	150.844	166.6681	250.2619
207.6323	167.3597	164.4004	282.4334	158.0422	131.8996
183.9623	268.7335	83.33405	268.7335	153.171	182.7812
105.6427	249.8866	65.3726	225.691	189.7191	220.5794
135.3037	230.2822	287.6003	293.4642	207.8755	297.3012
94.99264	262.2898	139.8168	158.7258	239.4116	221.3634
99.0842	159.6329	162.0119	141.4083	243.4482	293.1689
184.4717	88.6236	248.1181	89.67627	221.3308	344.078
129.9699	153.3594	196.3018	85.97752	312.0851	358.1613
215.2123	201.5277	140.1262	159.0893	193.7477	182.7022
212.274	170.7755	223.4085	285.7874	158.3616	148.3832
126.5365	121.2338	259.3003	146.6706	197.475	223.0204
202.3856	126.5365	139.8168	208.6727	209.8456	241.8118
103.7814	88.70502	228.52	255.3447	280.7157	150.5566
118.7066	159.0893	225.755	165.8866	278.3138	255.9377
333.2712	142.2735	117.0531	110.4532	125.85	132.3367
222.6316	120.1572	131.8996	176.0211	322.975	139.5066
340.4078	205.641	107.4719	91.82403	318.6773	253.472
294.054	164.9702	83.67983	150.844	169.6729	390.7051
199.1133	176.0211	79.06675	282.4334	208.2918	179.3525
175.6516	275.1837	118.0358	194.6399	232.0622	120.1572
305.6107	221.3308	175.1578	274.106	245.6914	170.1403
227.9823	302.6436	147.9935	211.2855	170.1403	295.1077
300.4652	245.6914	215.2123	104.3363	203.4174	150.844
183.8052	170.9867	212.41	206.7962	239.8936	300.9453
124.6396	252.3016	297.9318	151.703	220.3174	278.2879
267.6298	176.308	187.7301	117.0531	204.6204	131.7353
229.5601	123.5927	300.9693	82.02437	152.9352	314.0911
288.0267	54.00396	339.2182	268.6797	160.3548	223.5377
190.3269	68.50016	98.57288	196.1546	374.7095	307.7527
259.7176	64.48314		238.0509	108.8733	322.6171
276.6227			189.9853	180.1958	202.9555
329.2396			178.6266	78.33293	214.3047
193.4867			199.9815	293.2427	258.4358
180.6359			116	299.2132	120.1572
105.3691			78.33293	227.6338	215.5475
156.4814					119.2527
105.5744					276.6227
105.5744					105.6427
120.1572					268.2764
					107.539
					455.0296

					267.0898
					113.9912
					110.7795
					230.8145
					255.4578
					194.9734
8771.82	6283.404	6603.533	7442.483	8880.674	11978.84
8326.793					
2086.593					
45	36	35	41	41	57
42.5					
7.993748					
194.9293	174.539	188.6724	181.524	216.6018	226.0159
197.0471					
20.21771					

Fugure 1B Source Data 7

	Mock	anti-miR-10b	Control
Total Diameter	8326.793	2073.513098	7268.299
	2086.593	505.3679809	1678.65
N of spheres			
	42.5	21.42857143	42.28571
	7.993748	3.309438163	7.521398
Average diameter			
	197.0471	96.23171988	172.0254
	20.21771	14.41735466	11.47823

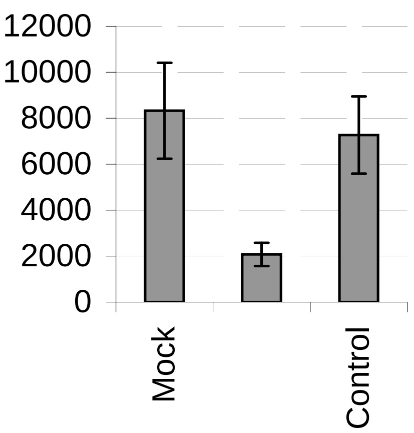

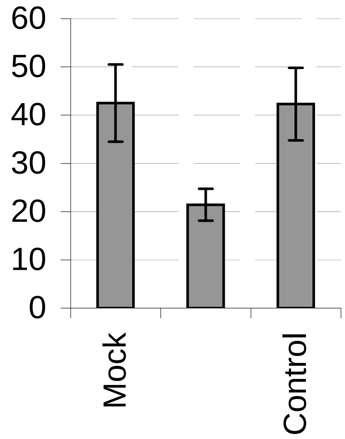

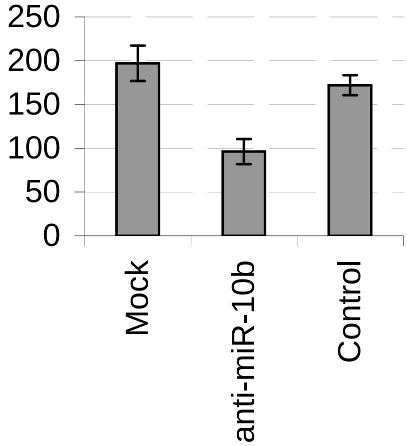

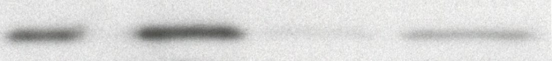

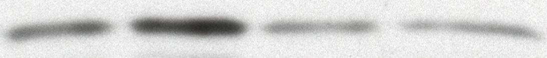

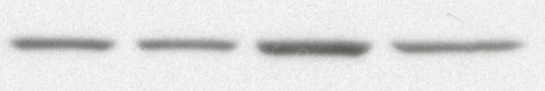

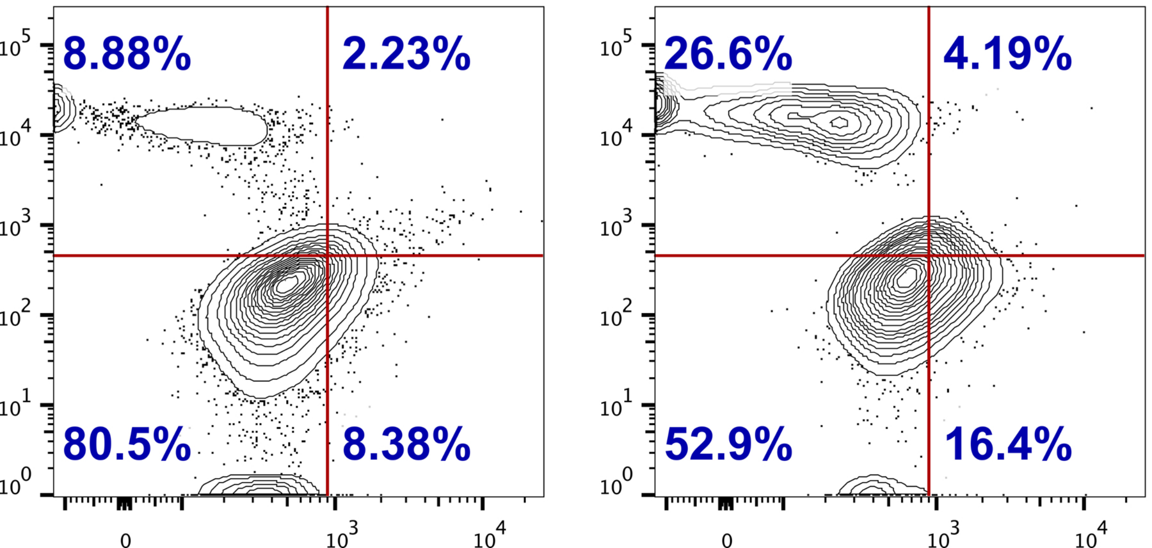
8.88%
2.23%
80.5%
8.38%
26.6%
4.19%
52.9%
16.4%
10^5
10^4
10^3
10^2
10^1
10^0
0
10^3
10^4
