# Supplementary material for: Therapeutic potential of targeting microRNA‐10b in established intracranial glioblastoma: first steps toward the clinic
Source: EMBO Mol Med. 2016 Feb 10;8(3):268–87. doi: 10.15252/emmm.201505495 (PMC4772951; doi:10.15252/emmm.201505495)

Figure 2 Panel C Source Data

| | 6seed | 7seed | 8seed | 9seed |
|---|---|---|---|---|
| 5UTR | 87 | 97 | 99 | 97 |
| CDS | 0 | 39 | 82 | 79 |
| 3UTR | 0 | 4 | 5 | 6 |

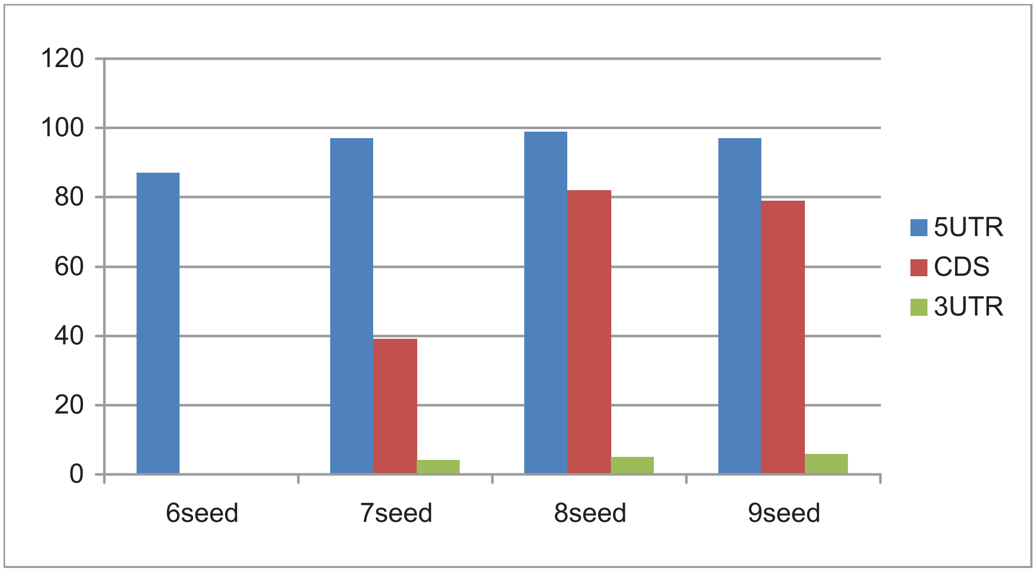

Figure 2 Panel D Source Data

| | | UP/Unchang | Down/Unchanged |
|---|---|---|---|
| ACAGGGTA | 1_8 | 0.8599113 | 0.3810806 |
| TACAGGGT | 2_9 | 1.6476623 | 0.5354671 |
| CTACAGGG | 3_10 | 1.4392623 | 0.3714406 |
| TCTACAGG | 4_11 | 1.3210332 | 0.7359724 |
| TTCTACAG | 5_12 | 1.4370984 | 0.9340736 |
| GTTCTACA | 6_13 | 1.2051954 | 0.9613757 |
| GGTTCTAC | 7_14 | 0.6490791 | 1.5820619 |
| CGGTTCTA | 8_15 | 0.4327194 | 0.4218832 |
| TCGGTTCT | 9_16 | 1.3387256 | 1.0040008 |
| TTCGGTTC | 10_17 | 1.6283914 | 0.6106204 |
| ATTCGGTT | 11_18 | 1.7849675 | 1.2430486 |
| AATTCGGT | 12_19 | 3.2953246 | 1.3769154 |
| AAATTCGG | 13_20 | 3.3659387 | 0.2983317 |
| CAAATTCG | 14_21 | 1.5521456 | 0.7566383 |

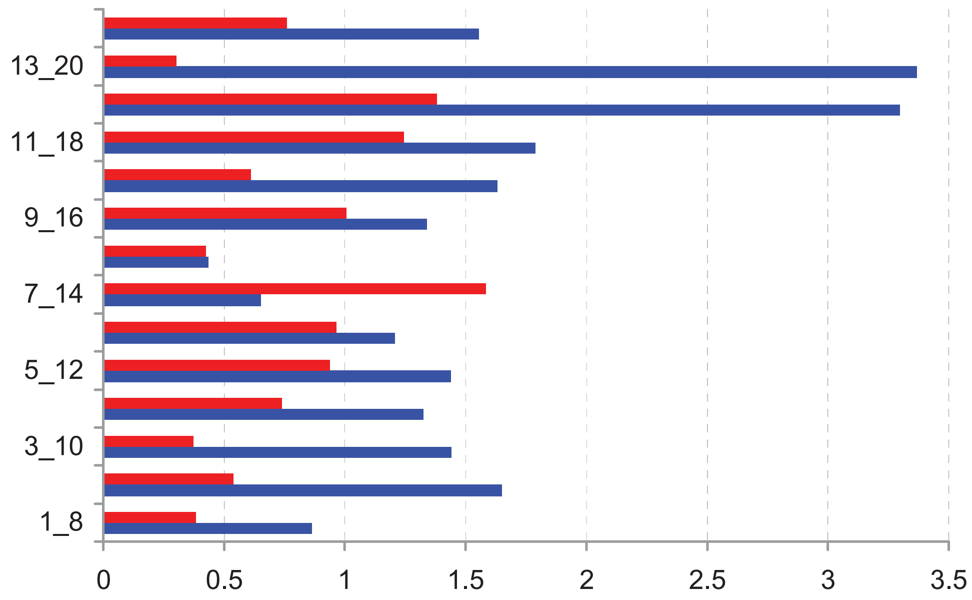

Supplement: Supplementary file 8 — Source Data for Figure 2 [file EMMM-8-268-s006.pdf]
