# Supplementary material for: Therapeutic potential of targeting microRNA‐10b in established intracranial glioblastoma: first steps toward the clinic
Source: EMBO Mol Med. 2016 Feb 10;8(3):268–87. doi: 10.15252/emmm.201505495 (PMC4772951; doi:10.15252/emmm.201505495)
Supplement: Supplementary file 10 — Source Data for Figure 4 [file EMMM-8-268-s008.pdf]

Figure 4 Panel B Source Data

Sample Name	MBNL1			Average	SD	SE	**p-val**
GBM4 control 1 day1	22.185	0.000	2.577	2.329	0.179089	0.089544	**0.00079951**
GBM4 control 1 day1	22.323	0.000	2.210				*******
GBM4 control 2 day1	22.120	0.000	2.341				
GBM4 control 2 day1	22.143	0.000	2.187				
GBM4 miR-10b-i 1day1	21.879	0.000	2.977	3.046	0.193531	0.096765	
GBM4 miR-10b-i 1 day1	21.966	0.000	2.898	1.308125			
GBM4 miR-10b-i 2 day1	21.886	0.000	2.979				
GBM4 miR-10b-i 2 day1	21.717	0.000	3.331				
GBM6 control 1 day1	21.664	0.000	2.063	2.670	0.45855	0.229275	**0.00023793**
GBM6 control 1 day1	21.533	0.000	3.119				*******
GBM6 control 2 day1	21.864	0.000	2.593				
GBM6 control 2 day1	21.673	0.000	2.906				
GBM6 miR-10b-i 1 day1	21.540	0.000	4.158	4.607	0.330632	0.165316	
GBM6 miR-10b-i 1 day1	21.369	0.000	4.646	1.725213			
GBM6 miR-10b-i 2 day1	21.280	0.000	4.955				
GBM6 miR-10b-i 2 day1	21.386	0.000	4.668				
GBM8 control 1 day1	22.393	0.000	2.975	3.035	0.141084	0.070542	**0.01187401**
GBM8 control 1 day1	22.277	0.000	2.958				*
GBM8 control 2 day1	22.246	0.000	2.961				
GBM8 control 2 day1	22.154	0.000	3.246				
GBM8 miR-10b-i 1 day1	22.900	0.000	3.823	3.890	0.55029	0.275145	
GBM8 miR-10b-i 1 day1	22.837	0.000	3.668	1.28155			
GBM8 miR-10b-i 2 day1	22.996	0.000	4.671				
GBM8 miR-10b-i 2 day1	23.225	0.000	3.396				
BT74 control 1 day 1	21.975	0.000	5.901	5.808	0.286474	0.143237	**0.00382048**
BT74 control 1 day 1	21.965	0.000	5.441				******
BT74 control 2 day 1	22.316	0.000	6.126				
BT74 control 2 day 1	22.33	0.000	5.763				
BT74 miR_10b-i 1 day 1	22.285	0.000	7.012	6.997	0.531519	0.265759	
BT74 miR-10b-i 1 day1	22.15	0.000	6.620	1.204721			
BT74 miR-10b-i 2 day 1	22.375	0.000	6.611				
BT74 miR-10b-i 2 day 1	22.189	0.000	7.743				
LN215 control 1 day1	20.784	0.000	7.310	6.986	0.321767	0.160883	**0.0092289**
LN215 control 1 day 1	20.84	0.000	6.547				******
LN215 control 2 day 1	20.364	0.000	7.100				
LN215 control 2 day 1	20.339	0.000	6.988				
LN215 miR-10b-i 1 day1	20.47	0.000	8.116	8.811	1.091994	0.545997	
LN215 miR-10b-i 1 day1	20.482	0.000	8.906	1.261216			
LN215 miR-10b-i 2 day1	20.747	0.000	7.906				
LN215 miR-10b-i 2 day1	20.356	0.000	10.316				
U251 control 1 day 1	19.309	0.000	4.322	4.235	0.069085	0.034543	**0.01940767**
U251 control 1 day 1	19.315	0.000	4.184				*****
U251 control 2 day 1	18.821	0.000	4.175				
U251 control 2 day 1	18.768	0.000	4.257				
U251 miR-10b-i 1 day1	19.572	0.000	6.246	5.291	0.798964	0.399482	
U251 miR-10b-i 1 day1	19.548	0.000	5.648	1.249473			
U251 miR-10b-i 2 day 1	19.412	0.000	4.547				
U251 miR-10b-i 2 day1	19.303	0.000	4.724				

Sample Name	GAPDH	
GBM4 control 1 day1	16.907	0.000
GBM4 control 1 day1	16.823	0.000
GBM4 control 2 day1	16.703	0.000
GBM4 control 2 day1	16.628	0.000
GBM4 miR-10b-i 1day1	16.809	0.000
GBM4 miR-10b-i 1 day1	16.857	0.000
GBM4 miR-10b-i 2 day1	16.817	0.000
GBM4 miR-10b-i 2 day1	16.809	0.000
GBM6 control 1 day1	16.065	0.000
GBM6 control 1 day1	16.530	0.000
GBM6 control 2 day1	16.595	0.000
GBM6 control 2 day1	16.568	0.000
GBM6 miR-10b-i 1 day1	16.952	0.000
GBM6 miR-10b-i 1 day1	16.941	0.000
GBM6 miR-10b-i 2 day1	16.945	0.000
GBM6 miR-10b-i 2 day1	16.965	0.000
GBM8 control 1 day1	17.322	0.000
GBM8 control 1 day1	17.198	0.000
GBM8 control 2 day1	17.168	0.000
GBM8 control 2 day1	17.209	0.000
GBM8 miR-10b-i 1 day1	18.191	0.000
GBM8 miR-10b-i 1 day1	18.068	0.000
GBM8 miR-10b-i 2 day1	18.576	0.000
GBM8 miR-10b-i 2 day1	18.345	0.000
BT74 control 1 day 1	17.892	0.000
BT74 control 1 day 1	17.765	0.000
BT74 control 2 day 1	18.287	0.000
BT74 control 2 day 1	18.213	0.000
BT74 miR_10b-i 1 day 1	18.451	0.000
BT74 miR-10b-i 1 day1	18.233	0.000
BT74 miR-10b-i 2 day 1	18.456	0.000
BT74 miR-10b-i 2 day 1	18.498	0.000
LN215 control 1 day1	17.010	0.000
LN215 control 1 day 1	16.907	0.000
LN215 control 2 day 1	16.548	0.000
LN215 control 2 day 1	16.500	0.000
LN215 miR-10b-i 1 day1	16.847	0.000
LN215 miR-10b-i 1 day1	16.993	0.000
LN215 miR-10b-i 2 day1	17.086	0.000
LN215 miR-10b-i 2 day1	17.079	0.000
U251 control 1 day 1	14.777	0.000
U251 control 1 day 1	14.736	0.000
U251 control 2 day 1	14.239	0.000
U251 control 2 day 1	14.214	0.000
U251 miR-10b-i 1 day1	15.571	0.000
U251 miR-10b-i 1 day1	15.402	0.000
U251 miR-10b-i 2 day 1	14.953	0.000
U251 miR-10b-i 2 day1	14.899	0.000

Sample Name	PTBP2			Average	SD	SE	**p-val**
GBM4 control 1 day1	22.127	0.000	2.683015	2.637	0.413504	0.206752	**0.01305232**
GBM4 control 1 day1	21.787	0.000	3.203959				*
GBM4 control 2 day1	22.112	0.000	2.353578				
GBM4 control 2 day1	22.066	0.000	2.306742				
GBM4 miR-10b-i 1day1	21.769	0.000	3.212858	3.265	0.109747	0.054874	
GBM4 miR-10b-i 1 day1	21.73	0.000	3.412566	1.238129			
GBM4 miR-10b-i 2 day1	21.802	0.000	3.157659				
GBM4 miR-10b-i 2 day1	21.741	0.000	3.275822				
GBM6 control 1 day1	22.441	0.000	1.204018	1.423	0.200406	0.100203	**0.00037703**
GBM6 control 1 day1	22.494	0.000	1.601981				***
GBM6 control 2 day1	22.576	0.000	1.583213				
GBM6 control 2 day1	22.832	0.000	1.301212				
GBM6 miR-10b-i 1 day1	22.552	0.000	2.061731	2.225	0.157902	0.078951	
GBM6 miR-10b-i 1 day1	22.459	0.000	2.182311	1.563734			
GBM6 miR-10b-i 2 day1	22.442	0.000	2.214309				
GBM6 miR-10b-i 2 day1	22.322	0.000	2.439957				
GBM8 control 1 day1	23.329	0.000	1.554937	1.473	0.057411	0.028705	**0.00024378**
GBM8 control 1 day1	23.329	0.000	1.426872				***
GBM8 control 2 day1	23.285	0.000	1.440786				
GBM8 control 2 day1	23.297	0.000	1.47004				
GBM8 miR-10b-i 1 day1	23.881	0.000	1.937043	1.902	0.111873	0.055937	
GBM8 miR-10b-i 1 day1	23.812	0.000	1.865881	1.291071			
GBM8 miR-10b-i 2 day1	24.195	0.000	2.034757				
GBM8 miR-10b-i 2 day1	24.165	0.000	1.770129				
BT74 control 1 day 1	22.135	0.000	5.281165	5.055	0.353823	0.176911	**0.24001555**
BT74 control 1 day 1	22.146	0.000	4.799405				
BT74 control 2 day 1	22.49	0.000	5.429642				
BT74 control 2 day 1	22.621	0.000	4.710419				
BT74 miR_10b-i 1 day 1	22.513	0.000	5.987094	5.299	0.543435	0.271717	
BT74 miR-10b-i 1 day1	22.657	0.000	4.658471	1.048287			
BT74 miR-10b-i 2 day 1	22.694	0.000	5.299497				
BT74 miR-10b-i 2 day 1	22.749	0.000	5.251956				
LN215 control 1 day1	24.629	0.000	0.508689	0.475	0.027739	0.01387	**0.00103249**
LN215 control 1 day 1	24.634	0.000	0.471999				**
LN215 control 2 day 1	24.257	0.000	0.477925				
LN215 control 2 day 1	24.325	0.000	0.441001				
LN215 miR-10b-i 1 day1	24.201	0.000	0.611259	0.675	0.07226	0.03613	
LN215 miR-10b-i 1 day1	24.095	0.000	0.727923	1.421695			
LN215 miR-10b-i 2 day1	24.151	0.000	0.746833				
LN215 miR-10b-i 2 day1	24.425	0.000	0.614659				
U251 control 1 day 1	23.042	0.000	0.325078	0.315	0.012216	0.006108	**0.01150531**
U251 control 1 day 1	22.997	0.000	0.32598				*
U251 control 2 day 1	22.592	0.000	0.305842				
U251 control 2 day 1	22.58	0.000	0.303098				
U251 miR-10b-i 1 day1	23.58	0.000	0.388196	0.370	0.033873	0.016936	
U251 miR-10b-i 1 day1	23.67	0.000	0.324402	1.17335			
U251 miR-10b-i 2 day 1	22.913	0.000	0.401607				
U251 miR-10b-i 2 day1	23	0.000	0.364214				

Figure 4 Panel B Source Data

Sample Name	MBNL2			Average	SD	SE	**p-val**
GBM4 control 1 day1	21.997	0.000	2.936	2.826	0.1556	0.078	**0.00033**
GBM4 control 1 day1	21.951	0.000	2.8597				*******
GBM4 control 2 day1	21.806	0.000	2.9097				
GBM4 control 2 day1	21.895	0.000	2.597				
GBM4 miR-10b-i 1day1	21.699	0.000	3.3726	3.444	0.1129	0.056	
GBM4 miR-10b-i 1 day1	21.716	0.000	3.4458	1.21873			
GBM4 miR-10b-i 2 day1	21.715	0.000	3.3539				
GBM4 miR-10b-i 2 day1	21.604	0.000	3.6021				
GBM6 control 1 day1	24.346	0.000	0.3215	0.421	0.0701	0.035	**5.8E-05**
GBM6 control 1 day1	24.213	0.000	0.4866				*******
GBM6 control 2 day1	24.425	0.000	0.4395				
GBM6 control 2 day1	24.412	0.000	0.4352				
GBM6 miR-10b-i 1 day1	23.801	0.000	0.8675	1.021	0.1164	0.058	
GBM6 miR-10b-i 1 day1	23.594	0.000	0.9937	2.42639			
GBM6 miR-10b-i 2 day1	23.428	0.000	1.118				
GBM6 miR-10b-i 2 day1	23.466	0.000	1.1041				
GBM8 control 1 day1	25.599	0.000	0.3224	0.301	0.0205	0.01	**0.0004**
GBM8 control 1 day1	25.704	0.000	0.2751				***
GBM8 control 2 day1	25.494	0.000	0.3116				
GBM8 control 2 day1	25.606	0.000	0.2967				
GBM8 miR-10b-i 1 day1	26.069	0.000	0.4251	0.397	0.0231	0.012	
GBM8 miR-10b-i 1 day1	26.100	0.000	0.3821	1.31792			
GBM8 miR-10b-i 2 day1	26.635	0.000	0.375				
GBM8 miR-10b-i 2 day1	26.286	0.000	0.4069				
BT74 control 1 day 1	25.179	0.000	0.6403	0.669	0.04	0.02	**0.00051**
BT74 control 1 day 1	25.079	0.000	0.6284				*******
BT74 control 2 day 1	25.441	0.000	0.7022				
BT74 control 2 day 1	25.363	0.000	0.7041				
BT74 miR_10b-i 1 day 1	24.932	0.000	1.1195	0.989	0.1004	0.05	
BT74 miR-10b-i 1 day1	24.948	0.000	0.9519	1.47945			
BT74 miR-10b-i 2 day 1	25.282	0.000	0.8814				
BT74 miR-10b-i 2 day 1	25.135	0.000	1.0048				
LN215 control 1 day1	22.979	0.000	1.5964	1.411	0.1374	0.069	**5.4E-07**
LN215 control 1 day 1	23.064	0.000	1.4014				*******
LN215 control 2 day 1	22.853	0.000	1.2648				
LN215 control 2 day 1	22.676	0.000	1.3831				
LN215 miR-10b-i 1 day1	21.818	0.000	3.1885	3.318	0.1354	0.068	
LN215 miR-10b-i 1 day1	21.867	0.000	3.4102	2.35116			
LN215 miR-10b-i 2 day1	22.044	0.000	3.2173				
LN215 miR-10b-i 2 day1	21.933	0.000	3.4578				
U251 control 1 day 1	20.42	0.000	2.0012	1.959	0.1419	0.071	**0.00301**
U251 control 1 day 1	20.282	0.000	2.1404				******
U251 control 2 day 1	20.013	0.000	1.8275				
U251 control 2 day 1	19.957	0.000	1.8672				
U251 miR-10b-i 1 day1	20.861	0.000	2.5559	2.424	0.1734	0.087	
U251 miR-10b-i 1 day1	20.699	0.000	2.5436	1.23719			
U251 miR-10b-i 2 day 1	20.471	0.000	2.1823				
U251 miR-10b-i 2 day1	20.272	0.000	2.413				

Sample Name	SART3			Average	SD	SE	**p-val**
GBM4 control 1 day1	22.176	0.000	2.5934	2.341	0.1785	0.089	**0.00052**
GBM4 control 1 day1	22.260	0.000	2.3083				*******
GBM4 control 2 day1	22.152	0.000	2.2892				
GBM4 control 2 day1	22.152	0.000	2.1733				
GBM4 miR-10b-i 1day1	21.934	0.000	2.8656	2.913	0.0741	0.037	
GBM4 miR-10b-i 1 day1	21.956	0.000	2.9178	1.24448			
GBM4 miR-10b-i 2 day1	21.868	0.000	3.0165				
GBM4 miR-10b-i 2 day1	21.940	0.000	2.8537				
GBM6 control 1 day1	22.288	0.000	1.3387	1.746	0.2769	0.138	**0.00065**
GBM6 control 1 day1	22.289	0.000	1.8466				*******
GBM6 control 2 day1	22.270	0.000	1.9573				
GBM6 control 2 day1	22.330	0.000	1.8427				
GBM6 miR-10b-i 1 day1	22.186	0.000	2.6571	2.583	0.1023	0.051	
GBM6 miR-10b-i 1 day1	22.302	0.000	2.4332	1.47928			
GBM6 miR-10b-i 2 day1	22.190	0.000	2.6369				
GBM6 miR-10b-i 2 day1	22.227	0.000	2.606				
GBM8 control 1 day1	23.134	0.000	1.78	1.679	0.0684	0.034	**0.00094**
GBM8 control 1 day1	23.136	0.000	1.6311				***
GBM8 control 2 day1	23.095	0.000	1.6436				
GBM8 control 2 day1	23.120	0.000	1.6619				
GBM8 miR-10b-i 1 day1	23.821	0.000	2.0193	2.055	0.1252	0.063	
GBM8 miR-10b-i 1 day1	23.746	0.000	1.9532	1.22376			
GBM8 miR-10b-i 2 day1	24.058	0.000	2.2375				
GBM8 miR-10b-i 2 day1	23.982	0.000	2.0095				
BT74 control 1 day 1	23.661	0.000	1.8338	1.818	0.1102	0.055	**0.0003**
BT74 control 1 day 1	23.676	0.000	1.6619				*******
BT74 control 2 day 1	23.99	0.000	1.9197				
BT74 control 2 day 1	23.964	0.000	1.8568				
BT74 miR_10b-i 1 day 1	23.788	0.000	2.474	2.420	0.1459	0.073	
BT74 miR-10b-i 1 day1	23.656	0.000	2.3308	1.3309			
BT74 miR-10b-i 2 day 1	23.914	0.000	2.275				
BT74 miR-10b-i 2 day 1	23.764	0.000	2.5988				
LN215 control 1 day1	23.069	0.000	1.4999	1.411	0.1064	0.053	**6.9E-05**
LN215 control 1 day 1	22.988	0.000	1.4772				*******
LN215 control 2 day 1	22.703	0.000	1.4033				
LN215 control 2 day 1	22.806	0.000	1.2639				
LN215 miR-10b-i 1 day1	22.301	0.000	2.2813	2.455	0.2193	0.11	
LN215 miR-10b-i 1 day1	22.192	0.000	2.7224	1.73999			
LN215 miR-10b-i 2 day1	22.547	0.000	2.2703				
LN215 miR-10b-i 2 day1	22.374	0.000	2.5471				
U251 control 1 day 1	20.884	0.000	1.4508	1.322	0.1125	0.056	**0.0054**
U251 control 1 day 1	20.914	0.000	1.3811				******
U251 control 2 day 1	20.594	0.000	1.2217				
U251 control 2 day 1	20.555	0.000	1.2336				
U251 miR-10b-i 1 day1	21.096	0.000	2.1717	1.845	0.2645	0.132	
U251 miR-10b-i 1 day1	21.096	0.000	1.9317	1.39609			
U251 miR-10b-i 2 day 1	20.821	0.000	1.7122				
U251 miR-10b-i 2 day1	20.896	0.000	1.5658				

Sample Name	DGCR14			Average	SD	SE	**p-val**
GBM4 control 1 day1	24.519	0.000	0.5112	0.464	0.0327	0.016	**0.01602**
GBM4 control 1 day1	24.62	0.000	0.4496				*****
GBM4 control 2 day1	24.543	0.000	0.4364				
GBM4 control 2 day1	24.394	0.000	0.4594				
GBM4 miR-10b-i 1day1	24.485	0.000	0.489	0.552	0.0544	0.027	
GBM4 miR-10b-i 1 day1	24.378	0.000	0.5444	1.18992			
GBM4 miR-10b-i 2 day1	24.312	0.000	0.5543				
GBM4 miR-10b-i 2 day1	24.139	0.000	0.6215				
GBM6 control 1 day1	24.287	0.000	0.3349	0.476	0.0978	0.049	**0.00064**
GBM6 control 1 day1	24.217	0.000	0.4853				*******
GBM6 control 2 day1	24.149	0.000	0.5321				
GBM6 control 2 day1	24.073	0.000	0.5505				
GBM6 miR-10b-i 1 day1	23.78	0.000	0.8802	0.856	0.0912	0.046	
GBM6 miR-10b-i 1 day1	24.057	0.000	0.7209	1.79916			
GBM6 miR-10b-i 2 day1	23.729	0.000	0.9074				
GBM6 miR-10b-i 2 day1	23.737	0.000	0.915				
GBM8 control 1 day1	25.676	0.000	0.3056	0.280	0.0288	0.014	**0.00015**
GBM8 control 1 day1	25.591	0.000	0.2975				***
GBM8 control 2 day1	25.864	0.000	0.2411				
GBM8 control 2 day1	25.708	0.000	0.2764				
GBM8 miR-10b-i 1 day1	25.913	0.000	0.4736	0.492	0.0486	0.024	
GBM8 miR-10b-i 1 day1	25.9	0.000	0.4389	1.75445			
GBM8 miR-10b-i 2 day1	26.222	0.000	0.4993				
GBM8 miR-10b-i 2 day1	25.84	0.000	0.5543				
BT74 control 1 day 1	24.564	0.000	0.9807	1.017	0.1455	0.073	**0.00044**
BT74 control 1 day 1	24.639	0.000	0.8525				*******
BT74 control 2 day 1	24.663	0.000	1.204				
BT74 control 2 day 1	24.812	0.000	1.0316				
BT74 miR_10b-i 1 day 1	24.459	0.000	1.5539	1.800	0.2103	0.105	
BT74 miR-10b-i 1 day1	24.082	0.000	1.7349	1.76929			
BT74 miR-10b-i 2 day 1	24.208	0.000	1.8556				
BT74 miR-10b-i 2 day 1	24.103	0.000	2.0546				
LN215 control 1 day1	26.186	0.000	0.1729	0.167	0.0131	0.007	**3.7E-06**
LN215 control 1 day 1	26.019	0.000	0.1807				*******
LN215 control 2 day 1	25.929	0.000	0.15				
LN215 control 2 day 1	25.735	0.000	0.166				
LN215 miR-10b-i 1 day1	24.674	0.000	0.4404	0.479	0.0417	0.021	
LN215 miR-10b-i 1 day1	24.649	0.000	0.4958	2.86178			
LN215 miR-10b-i 2 day1	24.882	0.000	0.45				
LN215 miR-10b-i 2 day1	24.639	0.000	0.5299				
U251 control 1 day 1	24.807	0.000	0.0956	0.083	0.0169	0.008	**0.00136**
U251 control 1 day 1	24.699	0.000	0.1002				******
U251 control 2 day 1	24.719	0.000	0.07				
U251 control 2 day 1	24.745	0.000	0.0676				
U251 miR-10b-i 1 day1	24.087	0.000	0.2732	0.272	0.0749	0.037	
U251 miR-10b-i 1 day1	23.968	0.000	0.2639	3.25809			
U251 miR-10b-i 2 day 1	23.046	0.000	0.3662				
U251 miR-10b-i 2 day1	23.992	0.000	0.1831				

Figure 4 Panel B Source Data

Sample Name	MBNL3			Average	SD	SE	**p-val**	
GBM4 control 1 day1	20.316	0.000	9.4143	8.768	0.51	0.255		**0.00786312**
GBM4 control 1 day1	20.310	0.000	8.9188				**	
GBM4 control 2 day1	20.299	0.000	8.2698					
GBM4 control 2 day1	20.190	0.000	8.467					
GBM4 miR-10b-i 1day1	20.186	0.000	9.6255	11.342	1.458	0.729		
GBM4 miR-10b-i 1 day1	19.783	0.000	13.158	1.2936				
GBM4 miR-10b-i 2 day1	19.996	0.000	11.041					
GBM4 miR-10b-i 2 day1	19.924	0.000	11.542					
GBM6 control 1 day1	23.669	0.000	0.514	0.699	0.14	0.07		**3.30092E-05**
GBM6 control 1 day1	23.440	0.000	0.8315				***	
GBM6 control 2 day1	23.813	0.000	0.6717					
GBM6 control 2 day1	23.569	0.000	0.7807					
GBM6 miR-10b-i 1 day1	23.029	0.000	1.4813	1.429	0.05	0.025		
GBM6 miR-10b-i 1 day1	23.063	0.000	1.4358	2.0423				
GBM6 miR-10b-i 2 day1	23.145	0.000	1.3602					
GBM6 miR-10b-i 2 day1	23.086	0.000	1.4368					
GBM8 control 1 day1	22.629	0.000	2.526	2.557	0.235	0.118		**0.002537012**
GBM8 control 1 day1	22.560	0.000	2.4315				**	
GBM8 control 2 day1	22.564	0.000	2.3749					
GBM8 control 2 day1	22.318	0.000	2.8976					
GBM8 miR-10b-i 1 day1	22.999	0.000	3.5698	3.314	0.261	0.131		
GBM8 miR-10b-i 1 day1	22.908	0.000	3.4915	1.2957				
GBM8 miR-10b-i 2 day1	23.551	0.000	3.1796					
GBM8 miR-10b-i 2 day1	23.397	0.000	3.0144					
BT74 control 1 day 1	24.982	0.000	0.734	0.832	0.124	0.062		**0.00074682**
BT74 control 1 day 1	24.893	0.000	0.7149				***	
BT74 control 2 day 1	25.008	0.000	0.9479					
BT74 control 2 day 1	24.962	0.000	0.9297					
BT74 miR_10b-i 1 day 1	24.888	0.000	1.1542	1.192	0.04	0.02		
BT74 miR-10b-i 1 day1	24.604	0.000	1.2082	1.4329				
BT74 miR-10b-i 2 day 1	24.881	0.000	1.1638					
BT74 miR-10b-i 2 day 1	24.831	0.000	1.2404					
LN215 control 1 day1	25.792	0.000	0.2272	0.212	0.018	0.009		**5.98871E-05**
LN215 control 1 day 1	25.686	0.000	0.2276				***	
LN215 control 2 day 1	25.563	0.000	0.1933					
LN215 control 2 day 1	25.456	0.000	0.2014					
LN215 miR-10b-i 1 day1	24.831	0.000	0.395	0.468	0.055	0.028		
LN215 miR-10b-i 1 day1	24.564	0.000	0.5259	2.2043				
LN215 miR-10b-i 2 day1	24.845	0.000	0.4616					
LN215 miR-10b-i 2 day1	24.752	0.000	0.49					
U251 control 1 day 1	25.443	0.000	0.0615	0.056	0.004	0.002		**0.000172952**
U251 control 1 day 1	25.569	0.000	0.0548				***	
U251 control 2 day 1	25.14	0.000	0.0523					
U251 control 2 day 1	25.053	0.000	0.0546					
U251 miR-10b-i 1 day1	25.463	0.000	0.1052	0.093	0.009	0.005		
U251 miR-10b-i 1 day1	25.484	0.000	0.0923	1.6686				
U251 miR-10b-i 2 day 1	25.025	0.000	0.0929					
U251 miR-10b-i 2 day1	25.149	0.000	0.0821					

Sample Name	SRSF11			Average	SD	SE	**p-val**	
GBM4 control 1 day1	20.969	0.000	5.9871	5.480	0.516	0.258		**0.026239392**
GBM4 control 1 day1	20.925	0.000	5.8234				*****	
GBM4 control 2 day1	20.959	0.000	5.2338					
GBM4 control 2 day1	20.986	0.000	4.8765					
GBM4 miR-10b-i 1day1	20.830	0.000	6.1597	6.120	0.123	0.062		
GBM4 miR-10b-i 1 day1	20.879	0.000	6.1554	1.1168				
GBM4 miR-10b-i 2 day1	20.890	0.000	5.9416					
GBM4 miR-10b-i 2 day1	20.815	0.000	6.2241					
GBM6 control 1 day1	21.091	0.000	3.0692	3.904	0.563	0.282		**1.24526E-05**
GBM6 control 1 day1	21.110	0.000	4.181				*******	
GBM6 control 2 day1	21.212	0.000	4.0752					
GBM6 control 2 day1	21.111	0.000	4.2896					
GBM6 miR-10b-i 1 day1	20.729	0.000	7.2947	7.467	0.247	0.124		
GBM6 miR-10b-i 1 day1	20.727	0.000	7.2494	1.9127				
GBM6 miR-10b-i 2 day1	20.628	0.000	7.7859					
GBM6 miR-10b-i 2 day1	20.695	0.000	7.5363					
GBM8 control 1 day1	22.538	0.000	2.6905	2.410	0.196	0.098		**0.000128496**
GBM8 control 1 day1	22.617	0.000	2.3373				***	
GBM8 control 2 day1	22.650	0.000	2.2375					
GBM8 control 2 day1	22.604	0.000	2.3765					
GBM8 miR-10b-i 1 day1	22.990	0.000	3.5922	3.590	0.237	0.119		
GBM8 miR-10b-i 1 day1	22.924	0.000	3.453	1.4893				
GBM8 miR-10b-i 2 day1	23.248	0.000	3.9227					
GBM8 miR-10b-i 2 day1	23.227	0.000	3.3913					
BT74 control 1 day 1	20.226	0.000	19.833	20.043	1.329	0.664		**0.000654218**
BT74 control 1 day 1	20.212	0.000	18.339				*******	
BT74 control 2 day 1	20.574	0.000	20.49					
BT74 control 2 day 1	20.430	0.000	21.509					
BT74 miR_10b-i 1 day 1	20.162	0.000	30.545	28.603	2.718	1.359		
BT74 miR-10b-i 1 day1	20.101	0.000	27.395	1.4271				
BT74 miR-10b-i 2 day 1	20.436	0.000	25.349					
BT74 miR-10b-i 2 day 1	20.182	0.000	31.122					
LN215 control 1 day1	20.739	0.000	7.5415	7.384	0.724	0.362		**1.57169E-05**
LN215 control 1 day 1	20.881	0.000	6.3637				*******	
LN215 control 2 day 1	20.178	0.000	8.0772					
LN215 control 2 day 1	20.227	0.000	7.552					
LN215 miR-10b-i 1 day1	19.684	0.000	13.995	15.925	1.354	0.677		
LN215 miR-10b-i 1 day1	19.547	0.000	17.028	2.1568				
LN215 miR-10b-i 2 day1	19.729	0.000	16.01					
LN215 miR-10b-i 2 day1	19.664	0.000	16.666					
U251 control 1 day 1	18.543	0.000	7.3506	6.487	0.956	0.478		**0.000730756**
U251 control 1 day 1	18.520	0.000	7.2594				*******	
U251 control 2 day 1	18.425	0.000	5.494					
U251 control 2 day 1	18.311	0.000	5.8436					
U251 miR-10b-i 1 day1	18.696	0.000	11.463	10.190	0.935	0.467		
U251 miR-10b-i 1 day1	18.679	0.000	10.316	1.5708				
U251 miR-10b-i 2 day 1	18.358	0.000	9.4405					
U251 miR-10b-i 2 day1	18.289	0.000	9.5391					

Sample Name	RSRC1			Average	SD	SE	**p-val**	
GBM4 control 1 day1	20.908	0.000	6.2457	6.253	0.14	0.07		**0.009099643**
GBM4 control 1 day1	20.792	0.000	6.3858				**	
GBM4 control 2 day1	20.687	0.000	6.3197					
GBM4 control 2 day1	20.672	0.000	6.0623					
GBM4 miR-10b-i 1day1	20.667	0.000	6.8965	6.932	0.398	0.199		
GBM4 miR-10b-i 1 day1	20.599	0.000	7.4739	1.1086				
GBM4 miR-10b-i 2 day1	20.757	0.000	6.5154					
GBM4 miR-10b-i 2 day1	20.678	0.000	6.8441					
GBM6 control 1 day1	20.342	0.000	5.1582	6.361	0.824	0.412		**0.001844677**
GBM6 control 1 day1	20.363	0.000	7.017				**	
GBM6 control 2 day1	20.493	0.000	6.7079					
GBM6 control 2 day1	20.498	0.000	6.5607					
GBM6 miR-10b-i 1 day1	20.223	0.000	10.359	9.091	0.854	0.427		
GBM6 miR-10b-i 1 day1	20.476	0.000	8.627	1.4291				
GBM6 miR-10b-i 2 day1	20.493	0.000	8.5496					
GBM6 miR-10b-i 2 day1	20.467	0.000	8.8266					
GBM8 control 1 day1	21.234	0.000	6.6431	6.157	0.698	0.349		**0.000325252**
GBM8 control 1 day1	21.459	0.000	5.2157				***	
GBM8 control 2 day1	21.217	0.000	6.0413					
GBM8 control 2 day1	21.103	0.000	6.7265					
GBM8 miR-10b-i 1 day1	21.499	0.000	10.097	9.528	0.775	0.388		
GBM8 miR-10b-i 1 day1	21.444	0.000	9.6321	1.5475				
GBM8 miR-10b-i 2 day1	21.901	0.000	9.9787					
GBM8 miR-10b-i 2 day1	21.918	0.000	8.4027					
BT74 control 1 day 1	19.488	0.000	33.079	31.422	2.134	1.067		**0.003060265**
BT74 control 1 day 1	19.585	0.000	28.322				**	
BT74 control 2 day 1	19.941	0.000	31.776					
BT74 control 2 day 1	19.834	0.000	32.511					
BT74 miR_10b-i 1 day 1	19.631	0.000	44.135	39.419	3.227	1.614		
BT74 miR-10b-i 1 day1	19.655	0.000	37.319	1.2545				
BT74 miR-10b-i 2 day 1	19.877	0.000	37.345					
BT74 miR-10b-i 2 day 1	19.861	0.000	38.877					
LN215 control 1 day1	20.621	0.000	8.1843	8.252	0.196	0.098		**0.002724921**
LN215 control 1 day 1	20.523	0.000	8.156				**	
LN215 control 2 day 1	20.097	0.000	8.5437					
LN215 control 2 day 1	20.122	0.000	8.1221					
LN215 miR-10b-i 1 day1	20.195	0.000	9.8209	10.867	1.218	0.609		
LN215 miR-10b-i 1 day1	19.98	0.000	12.613	1.3169				
LN215 miR-10b-i 2 day1	20.311	0.000	10.695					
LN215 miR-10b-i 2 day1	20.353	0.000	10.338					
U251 control 1 day 1	19.303	0.000	4.3405	4.096	0.235	0.117		**0.233594419**
U251 control 1 day 1	19.335	0.000	4.1263					
U251 control 2 day 1	18.833	0.000	4.1406					
U251 control 2 day 1	18.941	0.000	3.776					
U251 miR-10b-i 1 day1	20.295	0.000	3.7839	3.955	0.276	0.138		
U251 miR-10b-i 1 day1	20.169	0.000	3.6727	0.9657				
U251 miR-10b-i 2 day 1	19.501	0.000	4.2748					
U251 miR-10b-i 2 day1	19.511	0.000	4.0893					

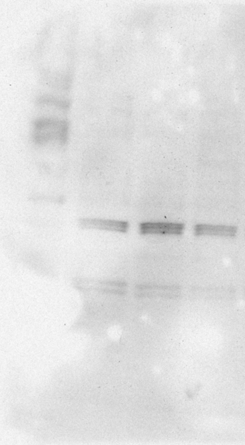

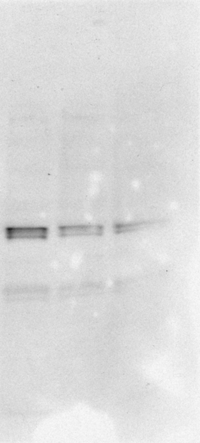

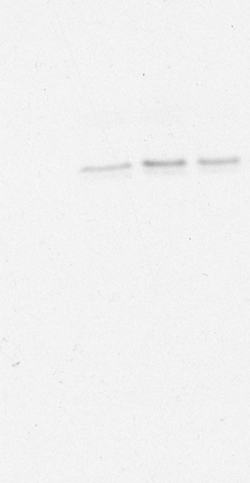

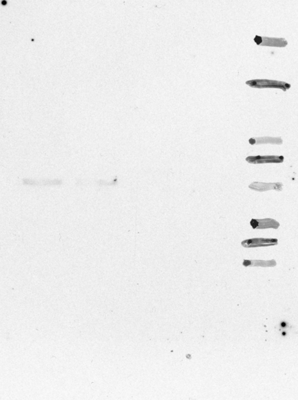

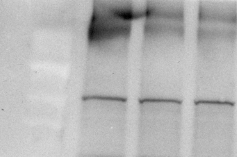

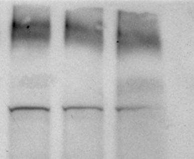

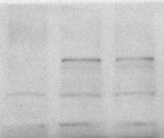

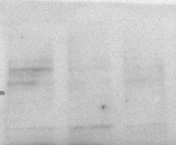

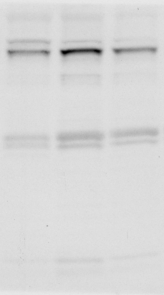

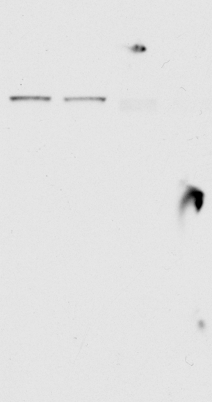

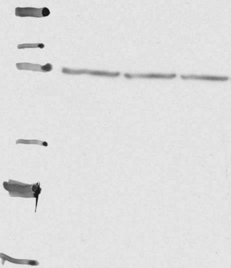

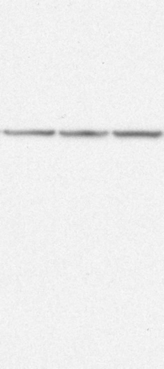

MBNL1				
GBM4			normalized to beta actin	
	1	12617.63	1.504925	
	2	7177.61	0.906037	0.602048
GBM8				
	1	11782.2	1.344774	
	2	6814.175	0.678039	0.504203
MBNL2				
GBM4				
	1	8753.64	1.044061	
	2	5239.74	0.661418	0.633505
GBM8				
	1	9537.589	1.088583	
	2	7474.004	0.743694	0.683176
MBNL3				
GBM4				
	1	6146.782	0.733137	
	2	5622.246	0.709702	0.968035
GBM8				
	1	4437.426	0.506471	
	2	3104.861	0.308947	0.609999
SART3				
GBM4				
	1	3572.962	0.426153	
	2	2438.205	0.307777	0.722222
GBM8				
	1	5707.882	0.651476	
	2	3934.811	0.39153	0.60099
RSRC1				
GBM4				
	1	5967.497	0.711753	
	2	2982.719	0.376512	0.528992
GBM8				
	1	12297.63	1.403604	
	2	8123.903	0.808362	0.575919
beta-actin				
GBM4				
	1	8384.225		
	2	7921.983		
GBM8				
	1	8761.468		
	2	10049.83		

	MBNL1		MBNL1 mutant		MBNL2		MBNL2 mutant	
control	3077	0.729814	1171	0.277742	21395	5.074547	9609	2.279099
control	3501	0.83038	1814	0.430251	16539	3.922782	6439	1.527226
control	2024	0.48006	1665	0.394911	17464	4.142177	5718	1.356217
control	2477	0.587504	1117	0.264934	17724	4.203845	7200	1.707723
control	3514	0.833464	1590	0.377122	11713	2.778134	7316	1.735236
		0.692245		0.348992		4.024297		1.7211
		0.155265		0.082978		0.823466		0.3473
		0.069437		0.037109		0.368265		0.155317
miR-10b	1878	0.597941	1742	0.55464	7417	2.361518	7038	2.240847
miR-10b	2232	0.710652	1643	0.523119	10597	3.374007	6800	2.16507
miR-10b	1766	0.562281	1157	0.36838	10134	3.226591	7001	2.229067
miR-10b	2094	0.666714	1241	0.395125	7050	2.244668	6935	2.208053
miR-10b	1997	0.63583	1366	0.434924	8829	2.811089	6846	2.179716
		0.634684		0.455238		2.803575		2.204551
		0.057859		0.080714		0.580836		0.03204
		0.025875		0.036096		0.259758		0.014329
		0.229817		0.030648		**0.011097**		0.140212
						**		

Viability

control	421614
anti-miR-10b	314077.6

	MBNL1	MBNL2	MBNL3	SART3	RSRC1
	MBNL1 mut	MBNL2 mu	MBNL3 mu	SART3 mu	RSRC1 mut
control	0.69224456	4.024297	14.3232	3.364974	14.25375
SE	0.06943656	0.368265	1.105319	0.341024	1.058321
miR-10b	0.63468391	2.803575	10.13571	2.042616	12.20686
SE	0.02615894	0.259758	0.371797	0.224241	1.195538
control mutant	0.34899221	1.7211	13.06385	4.847325	18.75052
SE	0.03271689	0.155317	1.404965	0.022327	2.344817
miR-10b mutan	0.45523781	2.204551	13.77589	3.728314	15.38008
SE	0.03609643	0.014329	1.336265	0.18227	1.274475

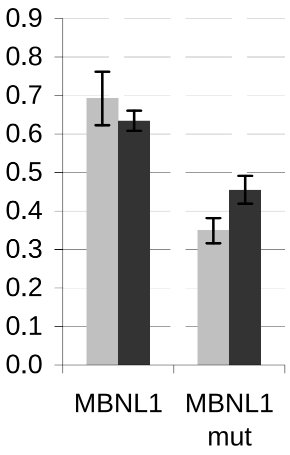

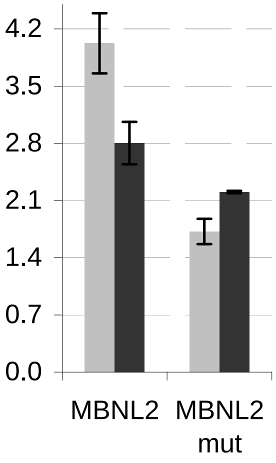

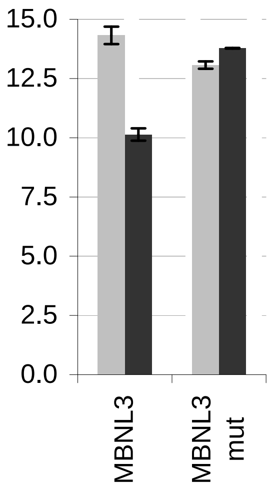

MBNL3		MBNL3 mutant		SART3		SART3 mutant		RSRC1
62605	14.84889	50643	12.0117	12824	3.041645	20702	4.910179	69422
49975	11.85326	52093	12.35561	14484	3.43537	20115	4.770952	47881
66247	15.71271	76757	18.20551	17346	4.11419	20494	4.860844	71061
73473	17.4266	40823	9.682553	9451	2.241624	20431	4.845902	53917
49643	11.77451	55079	13.06385	16831	3.99204	20443	4.848748	58198
	14.3232		13.06385		3.364974		4.847325	
	2.471569		3.141596		0.762553		0.049926	
	1.105319		1.404965		0.341024		0.022327	
34040	10.83809	53411	17.00567	8697	2.769061	9647	3.071534	44346
34794	11.07815	50761	16.16193	5164	1.64418	11512	3.665336	50121
29646	9.439069	37723	12.01073	5222	1.662646	11922	3.795877	32543
28858	9.188175	43904	13.97871	5572	1.774084	12492	3.977361	33231
31834	10.13571	30536	9.722438	7422	2.36311	12976	4.131463	31454
	10.13584		13.77589		2.042616		3.728314	
	0.831364		2.987979		0.501417		0.407567	
	0.371797		1.336265		0.224241		0.18227	
	**0.007891**		0.377749		**0.005939**		**0.001905**	
	**				**		**	

	miR-10b reporter
control	14.31724
SE	1.309811
miR-10b	1.023155
SE	0.212567

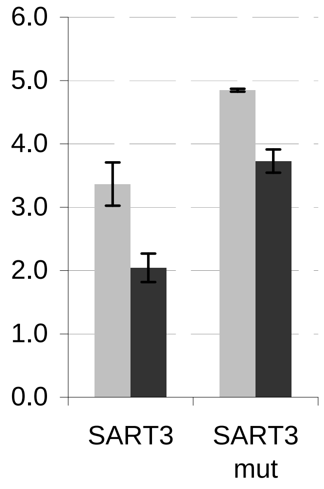

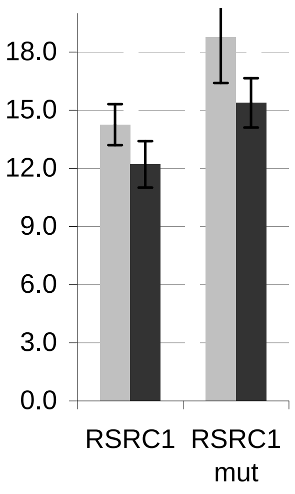

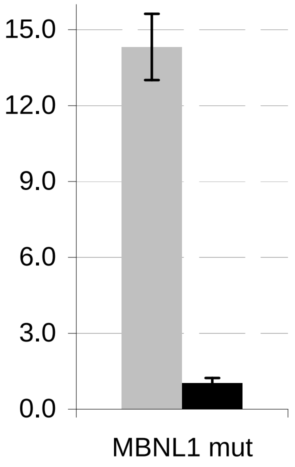

	RSRC1 mutant		miR-10b reporter	
16.46577	97950	23.23215	58012	13.75950514
11.3566	84452	20.03064	64364	15.26609648
16.85452	55109	13.07096	72636	17.22808066
12.78824	56576	13.41891	46442	11.01528887
13.80362	101187	23.99991		
14.25375		18.75052		14.31724279
2.366478		5.24317		2.619622838
1.058321		2.344817		1.309811419
14.11944	63619	20.25582	1938	0.61704496
15.95816	47872	15.24209	2205	0.702055798
10.36145	45248	14.40663	4136	1.316872009
10.58051	40588	12.92292	4575	1.456646383
10.01472	44200	14.07296		
12.20686		15.38008		1.023154787
2.673304		2.849812		0.425133125
1.195538		1.274475		0.212566563
0.117876		0.121094		2.86555E-05
*				***
