# Supplementary material for: Therapeutic potential of targeting microRNA‐10b in established intracranial glioblastoma: first steps toward the clinic
Source: EMBO Mol Med. 2016 Feb 10;8(3):268–87. doi: 10.15252/emmm.201505495 (PMC4772951; doi:10.15252/emmm.201505495)
Supplement: Supplementary file 11 — Source Data for Figure 5 [file EMMM-8-268-s009.pdf]

Figure 5 Panel B Source Data

Sample Name	miR-10b			miR-125b	
Control 1	25.564	5.43018	49.52212	22.142	0.109652
Control 1	25.676	5.024565	50.52762	22.283	0.099442
Control 2	25.533	5.548117	49.28235	22.104	0.112578
Control 2	25.71	4.90754	41.81653	22.044	0.117359
Control 3	25.846	4.466047	49.76292	22.431	0.089746
Control 3	25.803	4.601167	50.03967	22.396	0.09195
Control 4	26.073	3.815836	31.64705	22.005	0.120575
Control 4	26.146	3.627559	31.10338	22.053	0.116629
miR-10b-i 1	26.214	3.460543	5.872598	19.716	0.58927
miR-10b-i 1	26.283	3.29893	5.860398	19.782	0.562919
miR-10b-i 2	25.102	7.479801	12.18471	19.657	0.613868
miR-10b-i 2	25.24	6.797477	11.79415	19.748	0.576343
miR-10b-i 3	25.167	7.150278	10.19649	19.465	0.701249
miR-10b-i 3	25.374	6.194546	9.234276	19.529	0.670821
miR-10b-i 4	25.489	5.719935	22.89563	20.954	0.249827
miR-10b-i 4	25.575	5.388928	22.69024	21.027	0.2375
miR-10b-i 5	24.961	8.247739	18.10103	20.087	0.45565
miR-10b-i 5	24.875	8.754345	21.33279	20.238	0.41037

	miR-10b
Control 1	49.522
Control 1	50.528
Control 2	49.282
Control 2	41.81653
Control 3	49.763
Control 3	50.040
Control 4	31.64705
Control 4	31.10338
	44.213
	8.401
	2.970
miR-10b-i 1	5.873
miR-10b-i 1	5.860
miR-10b-i 2	12.185
miR-10b-i 2	11.794
miR-10b-i 3	10.196
miR-10b-i 3	9.234
miR-10b-i 4	22.89563
miR-10b-i 4	22.69024
miR-10b-i 5	18.10103
miR-10b-i 5	21.33279
	14.016
	6.690671
	2.115776

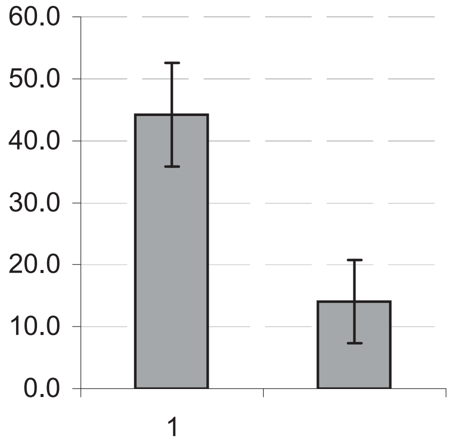

Figure 5 Panel C Source Data

	GAPDH		MBNL1			p	
Control 1	14.803	3.49827E-05	20.721	5.78572E-07	1.653882		4.09409E-06
Control 1	14.641	3.91399E-05	20.684	5.93602E-07	1.516616	***	
Control 2	14.972	3.11156E-05	20.713	5.8179E-07	1.869765		
Control 2	14.898	3.27533E-05	20.772	5.58477E-07	1.7051		
Control 3	14.823	3.45011E-05	20.507	6.71086E-07	1.945116		
Control 3	14.794	3.52016E-05	20.465	6.90909E-07	1.962722		
Control 4	15.163	2.72573E-05	20.776	5.56931E-07	2.043238		
Control 4	15.098	2.85134E-05	20.401	7.2225E-07	2.533017		
					1.903682		
					0.310195		
					0.10967		
miR-10b-i 1	16.063	1.46068E-05	21.410	3.58879E-07	2.456929		
miR-10b-i 1	15.882	1.65593E-05	21.328	3.79868E-07	2.293987		
miR-10b-i 2	16.491	1.08571E-05	21.480	3.41882E-07	3.148917		
miR-10b-i 2	16.422	1.1389E-05	21.399	3.61626E-07	3.175221		
miR-10b-i 3	15.798	1.75521E-05	20.719	5.79375E-07	3.300894		
miR-10b-i 3	15.686	1.8969E-05	20.657	6.04816E-07	3.188453		
miR-10b-i 4	16.045	1.47902E-05	20.842	5.32026E-07	3.597157		
miR-10b-i 4	15.92	1.61288E-05	21.091	4.47689E-07	2.77571		
miR-10b-i 5	16.497	1.08121E-05	21.325	3.80658E-07	3.520683		
miR-10b-i 5	16.563	1.03286E-05	21.508	3.3531E-07	3.246435		
					3.070438		
					0.429656		
					0.135869		

	MBNL1	MBNL2	MBNL3	SART3	RSRC1	PTBP2
control	1.903682	0.249103555	0.770391	0.735960495	3.344134	1.443642138
SE	0.10967	0.017351527	0.042563	0.033202707	0.094065	0.089208143
miR-10b-i	3.070438	0.692460015	0.844313	1.246401358	5.693499	1.830733423
SE	0.135869	0.028614222	0.045222	0.031191875	0.31362	0.087188373

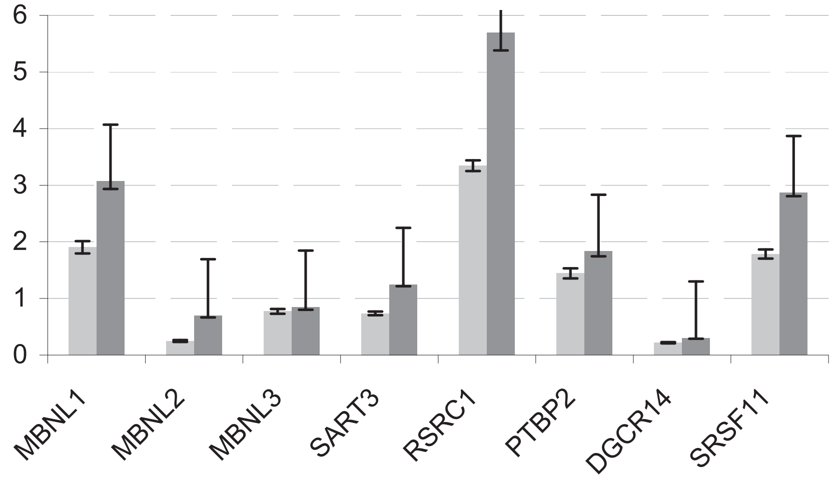

Figure 5 Panel C Source Data

MBNL2			**p**	MBNL3			**p**
23.499	8.44E-08	0.24113	6.2E-10	22.037	2.32E-07	0.664277	0.1302
23.535	8.23E-08	0.2102	***	22.042	2.32E-07	0.591667	
23.867	6.54E-08	0.21006		22.031	2.33E-07	0.749945	
23.903	6.37E-08	0.19464		21.946	2.48E-07	0.755685	
23.439	8.79E-08	0.25487		21.768	2.8E-07	0.811609	
23.461	8.66E-08	0.24602		21.870	2.61E-07	0.741159	
23.350	9.35E-08	0.34314		21.827	2.69E-07	0.986135	
23.514	8.35E-08	0.29277		21.955	2.46E-07	0.862655	
		0.2491				0.770391	
		0.04908				0.120385	
		0.01735				0.042563	
23.362	9.28E-08	0.63501		22.446	1.75E-07	1.19819	
23.534	8.23E-08	0.49719		22.578	1.6E-07	0.964503	
23.671	7.49E-08	0.68961		23.334	9.46E-08	0.871067	
23.607	7.83E-08	0.68723		23.397	9.05E-08	0.794907	
22.702	1.47E-07	0.83501		22.735	1.43E-07	0.816122	
22.723	1.44E-07	0.76147		22.708	1.46E-07	0.769427	
23.082	1.13E-07	0.76147		23.046	1.15E-07	0.780709	
23.046	1.15E-07	0.71591		23.072	1.13E-07	0.703126	
23.763	7.02E-08	0.6497		23.483	8.53E-08	0.788868	
23.738	7.15E-08	0.69201		23.610	7.81E-08	0.756208	
		0.69246				0.844313	
		0.09049				0.143003	
		0.02861				0.045222	

DGCR14	SRSF11
0.217907488	1.784651
0.011338367	0.080197
0.300590004	2.869135
0.011715556	0.063496

Figure 5 Panel C Source Data

SART3			**p**
21.930	3E-07	0.715417	3E-09
21.946	2E-07	0.632377	***
22.165	2E-07	0.683425	
22.207	2E-07	0.630625	
21.814	3E-07	0.786139	
21.934	2E-07	0.708999	
22.015	2E-07	0.865651	
21.951	2E-07	0.86505	
		0.73596	
		0.093911	
		0.033203	
22.506	2E-07	1.14938	
22.454	2E-07	1.051068	
22.833	1E-07	1.232729	
22.810	1E-07	1.194045	
21.979	2E-07	1.378268	
21.953	2E-07	1.29851	
22.285	2E-07	1.32304	
22.271	2E-07	1.225063	
22.803	1E-07	1.263877	
22.776	1E-07	1.348034	
		1.246401	
		0.098637	
		0.031192	

PTBP2			**p**
21.079	4.5E-07	1.29043	0.003655
21.238	4E-07	1.03301	**
20.677	6E-07	1.91701	
20.865	5.2E-07	1.59865	
20.911	5.1E-07	1.47004	
20.926	5E-07	1.42588	
21.284	3.9E-07	1.4368	
21.280	3.9E-07	1.37731	
		1.44364	
		0.25232	
		0.08921	
21.414	3.6E-07	2.45013	
21.604	3.1E-07	1.89455	
22.213	2.1E-07	1.89455	
22.165	2.1E-07	1.86717	
21.459	3.5E-07	1.97638	
21.821	2.7E-07	1.42292	
21.927	2.5E-07	1.69567	
21.930	2.5E-07	1.55171	
22.335	1.9E-07	1.74818	
22.354	1.9E-07	1.80607	
		1.83073	
		0.27571	
		0.08719	

Figure 5 Panel C Source Data

DGCR14			**p**
23.729	7E-08	0.206	7E-05
23.925	6E-08	0.16	***
23.934	6E-08	0.201	
23.774	7E-08	0.213	
23.603	8E-08	0.227	
23.579	8E-08	0.227	
23.691	7E-08	0.271	
23.808	7E-08	0.239	
		0.218	
		0.032	
		0.011	
24.515	4E-08	0.286	
24.412	4E-08	0.271	
24.581	4E-08	0.367	
24.628	4E-08	0.339	
24.110	6E-08	0.315	
24.379	5E-08	0.242	
24.415	4E-08	0.302	
24.416	4E-08	0.277	
24.973	3E-08	0.281	
24.816	3E-08	0.328	
		0.301	
		0.037	
		0.012	

SRSF11			**p**
20.784	6E-07	1.5832	4.94E-09
20.488	7E-07	1.7373	***
20.912	5E-07	1.6289	
20.985	5E-07	1.4711	
20.587	6E-07	1.8402	
20.564	6E-07	1.8326	
20.732	6E-07	2.1065	
20.687	6E-07	2.0775	
		1.7847	
		0.2268	
		0.0802	
21.138	4E-07	2.9667	
21.252	4E-07	2.4181	
21.507	3E-07	3.0905	
21.526	3E-07	2.9077	
20.839	5E-07	3.0374	
20.872	5E-07	2.747	
21.108	4E-07	2.9915	
21.097	4E-07	2.7642	
21.675	3E-07	2.7623	
21.619	3E-07	3.006	
		2.8691	
		0.2008	
		0.0635	

Figure 5 Panel C Source Data

RSRC1			p
19.771	1.12E-06	3.19509	5.97E-07
19.715	1.16E-06	2.96875	***
19.928	1E-06	3.221777	
19.923	1.01E-06	3.071313	
19.642	1.22E-06	3.542716	
19.653	1.21E-06	3.445841	
19.948	9.89E-07	3.6272	
19.862	1.05E-06	3.680384	
		3.344134	
		0.266056	
		0.094065	
19.696	1.18E-06	8.06043	
19.687	1.18E-06	7.154524	
20.47	6.89E-07	6.341641	
20.578	6.39E-07	5.609445	
20.045	9.24E-07	5.266544	
20.124	8.75E-07	4.61348	
20.146	8.62E-07	5.827416	
20.128	8.73E-07	5.410853	
20.555	6.49E-07	6.003716	
20.512	6.69E-07	6.474895	
		5.693499	
		0.991752	
		0.31362	

Figure 5 Panel D Source Data

Sample Name	miR-10b			
Control 1	25.564	5.43018	49.52211646	50.02487
Control 1	25.676	5.024565	50.52762264	
Control 2	25.533	5.548117	49.28235153	45.54944
Control 2	25.71	4.90754	41.81653412	
Control 3	25.846	4.466047	49.7629163	49.90129
Control 3	25.803	4.601167	50.03967304	
Control 4	26.073	3.815836	31.64705292	31.37522
Control 4	26.146	3.627559	31.10338372	
miR-10b-i 1	26.214	3.460543	5.872598258	5.866498
miR-10b-i 1	26.283	3.29893	5.860398168	
miR-10b-i 2	25.102	7.479801	12.18471288	11.98943
miR-10b-i 2	25.24	6.797477	11.79414937	
miR-10b-i 3	25.167	7.150278	10.19648771	9.715382
miR-10b-i 3	25.374	6.194546	9.23427582	
miR-10b-i 4	25.489	5.719935	22.89562747	22.79293
miR-10b-i 4	25.575	5.388928	22.69023601	
miR-10b-i 5	24.961	8.247739	18.10102509	19.71691
miR-10b-i 5	24.875	8.754345	21.33279237	

miR-125b		GAPDH	
22.1420002	0.109652	14.803	3.49827E-05
22.2830009	0.099442	14.641	3.91399E-05
22.1040001	0.112578	14.972	3.11156E-05
22.0440006	0.117359	14.898	3.27533E-05
22.4309998	0.089746	14.823	3.45011E-05
22.3959999	0.09195	14.794	3.52016E-05
22.0049992	0.120575	15.163	2.72573E-05
22.0529995	0.116629	15.098	2.85134E-05
19.7159996	0.58927	16.063	1.46068E-05
19.7819996	0.562919	15.882	1.65593E-05
19.6569996	0.613868	16.491	1.08571E-05
19.7479992	0.576343	16.422	1.1389E-05
19.4650002	0.701249	15.798	1.75521E-05
19.5289993	0.670821	15.686	1.8969E-05
20.9540005	0.249827	16.045	1.47902E-05
21.0270004	0.2375	15.92	1.61288E-05
20.0869999	0.45565	16.497	1.08121E-05
20.2380009	0.41037	16.563	1.03286E-05

Figure 5 Panel D Source Data

MBNL1				MBNL2			
20.721	5.78572E-07	1.653882	1.585249	23.499	8.43521E-08	0.241125	0.225664
20.684	5.93602E-07	1.516616		23.535	8.22733E-08	0.210203	
20.713	5.8179E-07	1.869765	1.787433	23.867	6.53607E-08	0.210057	0.202347
20.772	5.58477E-07	1.7051		23.903	6.375E-08	0.194637	
20.507	6.71086E-07	1.945116	1.953919	23.439	8.79343E-08	0.254874	0.250448
20.465	6.90909E-07	1.962722		23.461	8.66034E-08	0.246021	
20.776	5.56931E-07	2.043238	2.288127	23.35	9.35297E-08	0.343137	0.317955
20.401	7.2225E-07	2.533017		23.514	8.34797E-08	0.292773	
21.41	3.58879E-07	2.456929	2.375458	23.362	9.2755E-08	0.635012	0.566099
21.328	3.79868E-07	2.293987		23.534	8.23304E-08	0.497185	
21.48	3.41882E-07	3.148917	3.162069	23.671	7.48719E-08	0.689612	0.688419
21.399	3.61626E-07	3.175221		23.607	7.82681E-08	0.687226	
20.719	5.79375E-07	3.300894	3.244673	22.702	1.46561E-07	0.835005	0.798237
20.657	6.04816E-07	3.188453		22.723	1.44443E-07	0.761469	
20.842	5.32026E-07	3.597157	3.186433	23.082	1.12623E-07	0.761468	0.738691
21.091	4.47689E-07	2.77571		23.046	1.15468E-07	0.715913	
21.325	3.80658E-07	3.520683	3.383559	23.763	7.02464E-08	0.649704	0.670855
21.508	3.3531E-07	3.246435		23.738	7.14743E-08	0.692005	

SRSF11				RSRC1			
20.784	5.5385E-07	1.583214	1.660262	19.771	1.11773E-06	3.19509	3.08192
20.488	6.79982E-07	1.73731		19.715	1.16197E-06	2.96875	
20.912	5.06828E-07	1.628852	1.549956	19.928	1.00248E-06	3.221777	3.146545
20.985	4.81821E-07	1.471059		19.923	1.00596E-06	3.071313	
20.587	6.34886E-07	1.840192	1.836374	19.642	1.22227E-06	3.542716	3.494278
20.564	6.45089E-07	1.832555		19.653	1.21299E-06	3.445841	
20.732	5.74177E-07	2.106512	2.092011	19.948	9.88675E-07	3.6272	3.653792
20.687	5.92369E-07	2.07751		19.862	1.0494E-06	3.680384	
21.138	4.33339E-07	2.966693	2.692381	19.696	1.17737E-06	8.06043	7.607477
21.252	4.00415E-07	2.418069		19.687	1.18474E-06	7.154524	
21.507	3.35543E-07	3.090533	2.999096	20.47	6.88519E-07	6.341641	5.975543
21.526	3.31153E-07	2.907659		20.578	6.38859E-07	5.609445	
20.839	5.33133E-07	3.03744	2.892219	20.045	9.24387E-07	5.266544	4.940012
20.872	5.21077E-07	2.746999		20.124	8.75129E-07	4.61348	
21.108	4.42445E-07	2.991474	2.877831	20.146	8.61886E-07	5.827416	5.619135
21.097	4.45831E-07	2.764189		20.128	8.72706E-07	5.410853	
21.675	2.98659E-07	2.762274	2.884149	20.555	6.49125E-07	6.003716	6.239305
21.619	3.10479E-07	3.006024		20.512	6.68764E-07	6.474895	

Figure 5 Panel D Source Data

	miR-10b	MBNL1	MBNL2	RSRC1	SRSF11
Control	50.02487	1.585249	0.225664	3.08192	1.660262
Control	45.549443	1.787433	0.202347	3.146545	1.549956
Control	49.901295	1.953919	0.250448	3.494278	1.836374
Control	31.375218	2.288127	0.317955	3.653792	2.092011
miR-10b-i	5.8664982	2.375458	0.566099	7.607477	2.692381
miR-10b-i	11.989431	3.162069	0.688419	5.975543	2.999096
miR-10b-i	9.7153818	3.244673	0.798237	4.940012	2.892219
miR-10b-i	22.792932	3.186433	0.738691	5.619135	2.877831
miR-10b-i	19.716909	3.383559	0.670855	6.239305	2.884149

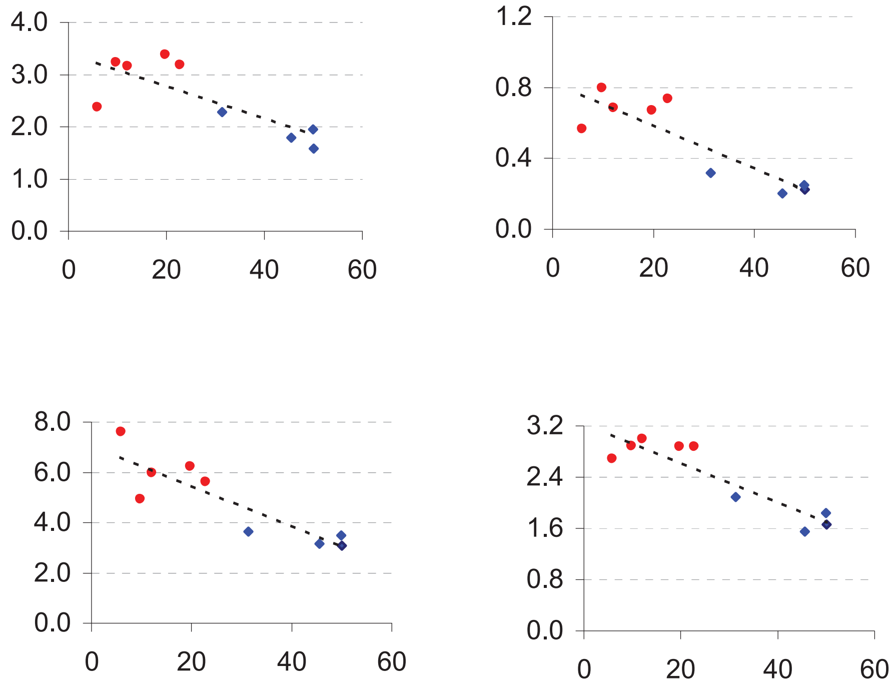

Figure 5 Panel E Source Data

**Cage1**	Total Flux [p/s]	Avg Radian	Total Flux [p.	Avg Radiance [p/s/cm²/sr]
BAT20110	474400.00	4696.00	79080.00	1148.00
BAT20110	8060000.00	79860.00	1849000.00	26850.00
BAT20110	19820000.00	196400.00	2332000.00	34200.00
BAT20110	44340000.00	438400.00	8641000.00	126900.00
BAT20110	812800.00	8028.00	134400.00	1955.00
**Cage2**	Total Flux [p/s]	Avg Radian	Total Flux [p.	Avg Radianc
BAT20110	33920000.00	336100.00	2716000.00	39640.00
BAT20110	2378000.00	23510.00	222200.00	3228.00
BAT20110	27410000.00	271600.00	3447000.00	50070.00
BAT20110	64100000.00	635800.00	2464000.00	36070.00
**Cage3**	Total Flux [p/s]	Avg Radian	Total Flux [p.	Avg Radianc
BAT20110	44130000.00	437300.00	3390000.00	33620.00
BAT20110	12650000.00	125300.00	1380000.00	13680.00
BAT20110	7329000.00	72380.00	338000.00	3345.00
BAT20110	87200.00	863.10	101900.00	1009.00
**Cage4**	Total Flux [p/s]	Avg Radian	Total Flux [p.	Avg Radiance [p/s/cm²/sr]
BAT20110	3123000.00	30940.00	2243000.00	22280.00
BAT20110	12100000.00	119600.00	249100.00	2471.00
BAT20110	7809000.00	77210.00	550100.00	5444.00
BAT20110	2362000.00	23370.00	1057000.00	10490.00

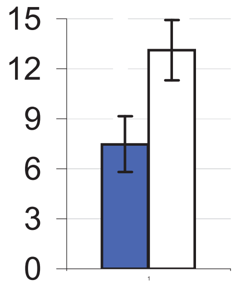

**Control**	**Total Flux [p/s**	**Avg Radian**	**Total Flux [**	**Avg Radia**	**Flux ratio**	**Radiance ratio**
BAT20110	33920000.00	336100.00	2716000.00	39640.00	12.49	8.48
BAT20110	27410000.00	271600.00	2464000.00	36070.00	11.12	7.53
BAT20110	64100000.00	635800.00	5746000.00	84250.00	11.16	7.55
BAT20110	44130000.00	437300.00	3390000.00	33620.00	13.02	13.01
BAT20110	12650000.00	125300.00	1380000.00	13680.00	9.17	9.16
BAT20110	7329000.00	72380.00	338000.00	3345.00	21.68	21.64
				Average	**13.11**	**11.23**
				SD	4.41	5.49
				SE	1.80	2.24
				**Ratio**	1.75	1.69108

**Treatment**	**Total Flux [p/s**	**Avg Radian**	**Total Flux [**	**Avg Radia**	**Flux ratio**	**Radiance ratio**
BAT20110	8060000.00	79860.00	1849000.00	26850.00	4.36	2.97
BAT20110	19820000.00	196400.00	2332000.00	34200.00	8.50	5.74
BAT20110	44340000.00	438400.00	8641000.00	126900.00	5.13	3.45
BAT20110	12100000.00	119600.00	2243000.00	22280.00	5.39	5.37
BAT20110	3123000.00	30940.00	249100.00	2471.00	12.54	12.52
BAT20110	7809000.00	77210.00	550100.00	5444.00	14.20	14.18
BAT20110	2362000.00	23370.00	1057000.00	10490.00	2.23	2.23
				Average	**7.48**	**6.64**
				SD	4.45	4.78
				SE	1.68	1.81
				**p-value**	**0.021679**	**0.06753**

Figure 5 Panel F Source Data

Treatments at days 20 and 25

	Day 5	Day 13	Day 20	Day 29	Ratio to day 5<br>13	20	29
**Cage1**	Total Flux |	Total Flux	Total Flux [p/	Total Flux [p/s]	Total Flu	Total Fl	Total Flux [p/s]
ROI 1	48590	63140	79080.00	474400.00	1.29944	1.6275	9.76333
ROI 2	101700	284300	1849000.00	8060000.00	2.79548	18.181	79.2527
ROI 3	125100	83280	2332000.00	19820000.00	0.66571	18.641	158.433
ROI 4	192000	653600	8641000.00	44340000.00	3.40417	45.005	230.938
ROI 5	82210	118200	134400.00	812800.00	1.43778	1.6348	9.88688
**Cage2**	Total Flux |	Total Flux	Total Flux [p/	Total Flux [p/s]			
ROI 1	93930	265600	2716000.00	33920000.00	2.82764	28.915	361.12
ROI 2	258100	907800	222200.00	2378000.00	3.51724	0.8609	9.21348
ROI 3	120700	84810	3447000.00	27410000.00	0.70265	28.558	227.092
ROI 4	114200	1067000	2464000.00	64100000.00	9.34326	21.576	561.296
ROI 5	64190	1019000	**5746000.00**	Too large too fa	15.8747	89.516	
**Cage3**	Total Flux |	Total Flux	Total Flux [p/	Total Flux [p/s]			
ROI 1	205800	61340	3390000.00	44130000.00	0.29806	16.472	214.431
ROI 2	59570	363400	1380000.00	12650000.00	6.10039	23.166	212.355
ROI 3	125500	120200	338000.00	7329000.00	0.95777	2.6932	58.3984
ROI 4	114700	260500	101900.00	87200.00	2.27114	0.8884	0.76024
**Cage4**	Total Flux |	Total Flux	Total Flux [p/	Total Flux [p/s]			
ROI 1	90110	192500	2243000.00	3123000.00	2.13628	24.892	34.6576
ROI 2	72750	137000	249100.00	12100000.00	1.88316	3.4241	166.323
ROI 3	79800	104800	550100.00	7809000.00	1.31328	6.8935	97.8571
ROI 4	127100	268700	1057000.00	2362000.00	2.11408	8.3163	18.5838
	112651.43	246311.43	2417314.29	**13944857.14**			
	119950	327058.33	2289166.67	**31589833.33**			

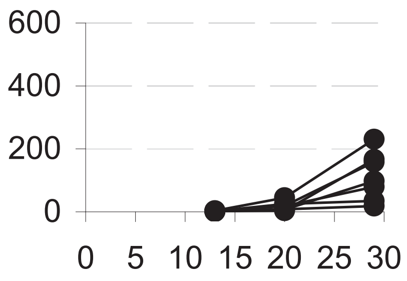

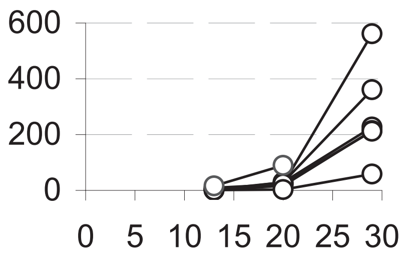

Figure 5 Panel G Source Data

Day	Group 1	Group 2
17	1	
18	1	
19	1	
19	1	
20	1	
21	1	
22	1	
18		1
19		1
20		1
23		1
24		0
24		0
24		0
