# Supplementary material for: Therapeutic potential of targeting microRNA‐10b in established intracranial glioblastoma: first steps toward the clinic
Source: EMBO Mol Med. 2016 Feb 10;8(3):268–87. doi: 10.15252/emmm.201505495 (PMC4772951; doi:10.15252/emmm.201505495)
Supplement: Supplementary file 12 — Source Data for Figure 6 [file EMMM-8-268-s010.pdf]

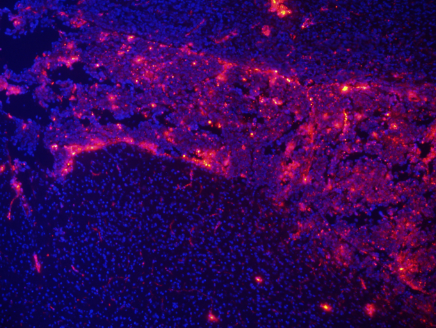

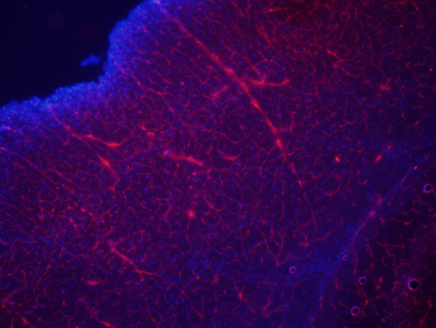

Figure 6 Panel B Source Data

Total Flux [p/s]

Day	6	10	14	17	24	28	31	34
a-10b	115700	230800	2.48E+05	636500	1740000	1773000	4844000	**11500000**
	696100	1027000	8.87E+05	748400	7103000	7891000	**47170000**	**1.36E+08**
	289800	414600	5.52E+05	531300	1838000	1883000	4116000	5364000
	681000	570400	5.77E+05	695700	3793000	4372000	**13090000**	**15410000**
	745400	525600	2.81E+05	379900	792100	1880000	5103000	9197000
	238400	190200	1.75E+05	157400	949700	1780000	3744000	8591000
	184200	187400	1.98E+05	304400	2089000	3882000	**10880000**	**18470000**
		1.994814	2.15E+00	5.50E+00	1.50E+01	1.53E+01	4.19E+01	99.39
		1.475363	1.27E+00	1.08E+00	1.02E+01	1.13E+01	6.78E+01	195.81
		1.430642	1.90E+00	1.83E+00	6.34E+00	6.50E+00	1.42E+01	18.51
		0.837592	8.47E-01	1.02E+00	5.57E+00	6.42E+00	1.92E+01	22.63
		0.705125	3.77E-01	5.10E-01	1.06E+00	2.52E+00	6.85E+00	12.34
		0.797819	7.32E-01	6.60E-01	3.98E+00	7.47E+00	1.57E+01	36.04
		1.017372	1.08E+00	1.65E+00	1.13E+01	2.11E+01	5.91E+01	100.27
Average		1.179818	1.19383	1.750542	7.648881	10.0916	3.21E+01	69.28341
SD		0.470681	0.637013	1.722437	4.789128	6.326493	24.10797	67.05414
SE		0.177901	0.240768	0.65102	1.81012	2.39119	9.111955	25.34408
Control	192600	183700	2.18E+05	282800	1187000	**29960000**	**1.35E+08**	**2.95E+08**
	208600	223000	3.88E+05	503200	2298000	4596000	**10520000**	**22590000**
	149300	395000	1.14E+06	1010000	4715000	9315000	**22480000**	**25100000**
	745100	562000	3.76E+05	525600	1950000	2389000	4616000	**12310000**
	786000	613200	6.55E+04	407300	1978000	2980000	**10160000**	**10840000**
	256000	236600	3.81E+05	1019000	849300	2763000	6447000	**19370000**
	485500	286200	2.23E+05	420200	3847000	7560000	**34400000**	**1.02E+08**
		0.95379	1.13E+00	1.47E+00	6.16E+00	1.56E+02	7.02E+02	1532.71
		1.069032	1.86E+00	2.41E+00	1.10E+01	2.20E+01	5.04E+01	108.29
		2.64568	7.60E+00	6.76E+00	3.16E+01	6.24E+01	1.51E+02	168.12
		0.754261	5.05E-01	7.05E-01	2.62E+00	3.21E+00	6.20E+00	16.52
		0.780153	8.33E-02	5.18E-01	2.52E+00	3.79E+00	1.29E+01	13.79
		0.924219	1.49E+00	3.98E+00	3.32E+00	1.08E+01	2.52E+01	75.66
		0.589495	4.60E-01	8.65E-01	7.92E+00	1.56E+01	7.09E+01	209.68
Average		1.102376	1.875476	2.387868	9.305007	39.04878	145.4476	303.54
SD		0.698185	2.601047	2.2798	10.30838	55.20505	250.2517	546.8685
SE		0.263889	0.983104	0.861683	3.896201	20.86555	94.58626	206.6969

p

0.141313

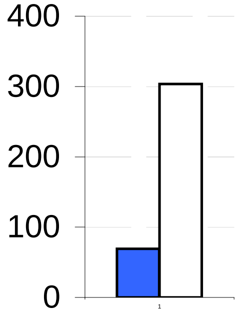

Figure 6 Panel C Source Data

Day	Group1	Group2
6	1	
6	1	
6	1	
7	1	
7	1	
7	1	
10	1	
6		1
7		1
7		1
10		1
11		1
13		1
15		0

Figure 6 Panel D Source Data

Avg Radiance [p/s/cm²/sr]

Day	6	10	14	17	24	28	31	34
miR-10b-i	1145	2284	2.46E+03	6300	17220	17550	47940	113900
	6889	13140	8.79E+03	7399	70300	78010	466900	1349000
	2874	4103	5.46E+03	5247	18250	18620	40700	53150
	6754	5640	5.71E+03	6893	37540	43310	129900	153000
	7401	5174	2.78E+03	3772	7856	18610	50500	90830
	2357	1887	1.73E+03	1558	9389	17630	37090	85110
	1825	1857	1.97E+03	3026	20740	38430	158200	268600
	1	1.99476	2.15E+00	5.50E+00	1.50E+01	1.53E+01	4.19E+01	9.95E+01
	1	1.907389	1.28E+00	1.07E+00	1.02E+01	1.13E+01	6.78E+01	1.96E+02
	1	1.427627	1.90E+00	1.83E+00	6.35E+00	6.48E+00	1.42E+01	1.85E+01
	1	0.835061	8.46E-01	1.02E+00	5.56E+00	6.41E+00	1.92E+01	2.27E+01
	1	0.699095	3.75E-01	5.10E-01	1.06E+00	2.51E+00	6.82E+00	1.23E+01
	1	0.800594	7.32E-01	6.61E-01	3.98E+00	7.48E+00	1.57E+01	3.61E+01
	1	1.017534	1.08E+00	1.66E+00	1.14E+01	2.11E+01	8.67E+01	1.47E+02
Control	1909	1822	2.14E+03	2793	11630	295600	1336000	2922000
	2055	2208	3.85E+03	4986	22720	45440	104000	223800
	1481	3918	1.12E+04	10020	46610	92190	330100	365700
	7399	10830	5.51E+03	7634	28460	35080	45780	122000
	7796	6062	9.61E+02	5925	29050	43350	99810	106500
	2528	2344	5.53E+03	14940	12470	40650	64020	192100
	4805	2836	3.25E+03	6172	56500	111400	503700	1494000
	1	0.954426	1.12E+00	1.46E+00	6.09E+00	1.55E+02	7.00E+02	1.53E+03
	1	1.074453	1.87E+00	2.43E+00	1.11E+01	2.21E+01	5.06E+01	1.09E+02
	1	2.64551	7.58E+00	6.77E+00	3.15E+01	6.22E+01	2.23E+02	2.47E+02
	1	1.463711	7.45E-01	1.03E+00	3.85E+00	4.74E+00	6.19E+00	1.65E+01
	1	0.777578	1.23E-01	7.60E-01	3.73E+00	5.56E+00	1.28E+01	1.37E+01
	1	0.927215	2.19E+00	5.91E+00	4.93E+00	1.61E+01	2.53E+01	7.60E+01
	1	0.590219	6.75E-01	1.28E+00	1.18E+01	2.32E+01	1.05E+02	3.11E+02

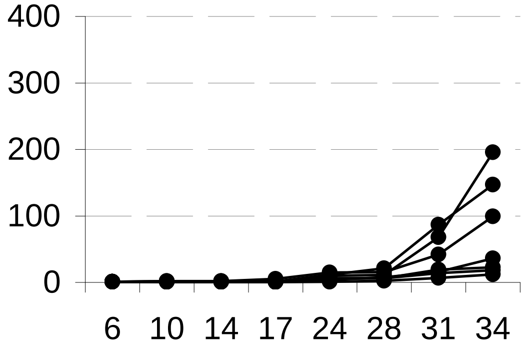

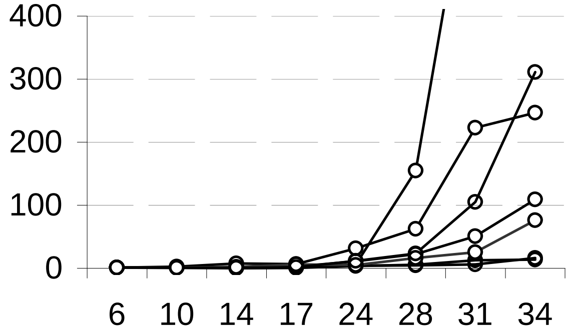

Figure 6 Panel E Source Data

Sample Name		miR10b					
4-1 T1	contol	26.160	1.33369E-08	16.91061	5.908448	4.597104	0.868771
4-1 T1	contol	26.001	1.48908E-08	17.19428			
4-1 T2	contol	25.232	2.53753E-08	11.81752		**p**	
4-1 T2	contol	25.317	2.39234E-08	11.45461		0.004298	
4-2 T1	contol	26.127	1.36455E-08	5.520716		**	
4-2 T1	contol	26.175	1.3199E-08	5.621115			
4-2 T2	contol	25.989	1.50152E-08	11.42288			
4-2 T2	contol	25.808	1.70223E-08	13.49971			
4-4 T	contol	25.618	1.94184E-08	3.903736			
4-4 T	contol	25.555	2.02852E-08	3.871402			
4-4 T1	contol	25.877	1.62273E-08	4.60709			
4-4 T1	contol	25.681	1.85887E-08	5.494001			
5-1 T1	contol	25.133	2.71777E-08	4.934338			
5-1 T1	contol	25.227	2.54634E-08	4.859666			
5-1 T2	contol	28.588	2.4783E-09	1.49263			
5-1 T2	contol	28.818	2.11309E-09	1.302114			
5-2 T2	contol	27.106	6.92278E-09	2.508552			
5-2 T2	contol	27.118	6.86544E-09	2.480887			
5-2 T3	contol	28.161	3.33192E-09	1.528225			
5-2 T3	contol	28.304	3.0175E-09	1.181692			
5-4 T1	contol	26.575	1.00029E-08	3.729172			
5-4 T1	**contol**	26.624	9.6689E-09	3.604641			
5-4 T2	contol	26.732	8.97152E-09	2.673734			
5-4 T2	contol	26.928	7.83185E-09	2.273408			
5-5 T2	contol	25.261	2.48703E-08	8.049259			
5-5 T2	contol	25.410	2.24299E-08	5.953989			
5-5 T3	contol	25.958	1.53413E-08	2.51552			
5-5 T3	contol	26.017	1.47266E-08	5.031039			
1-2 T1	anti-miR-10b	25.534	2.05826E-08	6.070673	3.468341	1.664812	0.303952
1-2 T1	anti-miR-10b	25.637	1.91644E-08	6.276049	0.587		0.051
1-2 T2	anti-miR-10b	26.117	1.37404E-08	3.03323			
1-2 T2	anti-miR-10b	26.213	1.28559E-08	1.589814			
1-3 T1	anti-miR-10b	28.508	2.61961E-09	1.303018			
1-3 T1	anti-miR-10b	28.540	2.56214E-09	1.200683			
1-3 T2	anti-miR-10b	25.625	1.93244E-08	4.069515			
1-3 T2	anti-miR-10b	25.621	1.93781E-08	4.075157			
2-1 T1	anti-miR-10b	27.459	5.42023E-09	0.968521			
2-1 T1	anti-miR-10b	27.197	6.49961E-09	1.035879			
2-1 T2	anti-miR-10b	25.969	1.52248E-08	5.186844			
2-1 T2	anti-miR-10b	25.919	1.57617E-08	5.605545			
2-3 T1	anti-miR-10b	26.504	1.05075E-08	2.127053			
2-3 T1	anti-miR-10b	26.476	1.07135E-08	1.918336			
2-3 T2	anti-miR-10b	25.437	2.2014E-08	5.422117			
2-3 T2	anti-miR-10b	25.284	2.4477E-08	6.297835			
2-4 T1	anti-miR-10b	26.150	1.34297E-08	5.648455			
2-4 T1	anti-miR-10b	26.275	1.23151E-08	5.811283			
2-4 T2	anti-miR-10b	26.975	7.58081E-09	3.407837			
2-4 T2	anti-miR-10b	27.050	7.19679E-09	3.545176			

3-1 T1	anti-miR-10b	25.630	1.92576E-08	3.624688

Figure 6 Panel E Source Data

3-1 T1	anti-miR-10b	26.102	1.3884E-08	2.754627
3-1 T2	anti-miR-10b	26.925	7.84816E-09	2.950288
3-1 T2	anti-miR-10b	26.761	8.79299E-09	3.356265
3-2 T1	anti-miR-10b	25.447	2.1862E-08	2.168738
3-2 T1	anti-miR-10b	25.557	2.02571E-08	2.006744
3-2 T2	anti-miR-10b	25.440	2.19683E-08	2.857704
3-2 T2	anti-miR-10b	25.500	2.10734E-08	2.883572
3-3 T1	anti-miR-10b	25.460	2.16659E-08	3.672743
3-3 T1	anti-miR-10b	25.441	2.19531E-08	3.181827

Figure 6 Panel E Source Data

miR125b

23.596	7.88671E-08
23.461	8.66034E-08
22.151	2.14726E-07
22.191	2.08854E-07
21.948	2.47169E-07
22.022	2.34811E-07
22.859	1.31448E-07
22.919	1.26094E-07
20.939	4.97431E-07
20.864	5.23974E-07
21.437	3.52225E-07
21.495	3.38345E-07
20.792	5.50788E-07
20.864	5.23974E-07
22.522	1.66036E-07
22.555	1.62281E-07
21.789	2.75967E-07
21.785	2.76733E-07
22.129	2.18026E-07
21.901	2.55354E-07
21.830	2.68235E-07
21.830	2.68235E-07
21.507	3.35543E-07
21.469	3.44498E-07
21.626	3.08977E-07
21.340	3.76721E-07
20.645	6.09868E-07
21.704	2.92715E-07
21.492	3.3905E-07
21.643	3.05357E-07
21.074	4.52996E-07
20.238	8.08639E-07
22.246	2.01042E-07
22.160	2.13391E-07
21.006	4.74858E-07
21.004	4.75517E-07
20.769	5.59639E-07
20.604	6.27448E-07
21.700	2.93528E-07
21.762	2.81181E-07
20.949	4.93995E-07
20.772	5.58477E-07
21.232	4.06005E-07
21.295	3.88657E-07
22.004	2.37759E-07
22.170	2.11917E-07
22.100	2.22452E-07
22.232	2.03002E-07

20.844	5.31289E-07

Figure 6 Panel E Source Data

20.920	5.04025E-07
21.842	2.66013E-07
21.864	2.61987E-07
19.920	1.00805E-06
19.918	1.00945E-06
20.311	7.6874E-07
20.384	7.3081E-07
20.693	5.8991E-07
20.467	6.89953E-07

Figure 6 Panel F Source Data

Sample Name		GAPDH	
4-1 T1	contol	16.386	0.000
4-1 T1	contol	16.372	0.000
4-1 T2	contol	16.563	0.000
4-1 T2	contol	16.541	0.000
4-2 T1	contol	17.370	0.000
4-2 T1	contol	17.355	0.000
4-2 T2	contol	16.975	0.000
4-2 T2	contol	16.973	0.000
4-4 T	contol	17.285	0.000
4-4 T	contol	17.419	0.000
4-4 T1	contol	17.599	0.000
4-4 T1	contol	17.689	0.000
5-1 T1	contol	17.362	0.000
5-1 T1	contol	17.367	0.000
5-1 T2	contol	19.482	0.000
5-1 T2	contol	19.576	0.000
5-2 T2	contol	18.610	0.000
5-2 T2	contol	18.675	0.000
5-2 T3	contol	19.006	0.000
5-2 T3	contol	18.963	0.000
5-4 T1	contol	17.856	0.000
5-4 T1	**contol**	**17.765**	0.000
5-4 T2	contol	17.836	0.000
5-4 T2	contol	17.916	0.000
5-5 T2	contol	16.727	0.000
5-5 T2	contol	16.679	0.000
5-5 T3	contol	16.963	0.000
5-5 T3	contol	17.631	0.000
1-2 T1	anti-miR-10b	17.305	0.000
1-2 T1	anti-miR-10b	17.343	0.000
1-2 T2	anti-miR-10b	17.860	0.000
1-2 T2	anti-miR-10b	17.697	0.000
1-3 T1	anti-miR-10b	19.469	0.000
1-3 T1	anti-miR-10b	19.518	0.000
1-3 T2	anti-miR-10b	17.751	0.000
1-3 T2	anti-miR-10b	17.726	0.000
2-1 T1	anti-miR-10b	18.147	0.000
2-1 T1	anti-miR-10b	18.126	0.000
2-1 T2	anti-miR-10b	16.959	0.000
2-1 T2	anti-miR-10b	16.974	0.000
2-3 T1	anti-miR-10b	18.478	0.000
2-3 T1	anti-miR-10b	18.604	0.000
2-3 T2	anti-miR-10b	17.499	0.000
2-3 T2	anti-miR-10b	17.438	0.000
2-4 T1	anti-miR-10b	17.953	0.000
2-4 T1	anti-miR-10b	18.207	0.000
2-4 T2	anti-miR-10b	18.502	0.000
2-4 T2	anti-miR-10b	18.266	0.000

3-1 T1	anti-miR-10b	17.711	0.000

Figure 6 Panel F Source Data

3-1 T1	anti-miR-10b	17.735	0.000
3-1 T2	anti-miR-10b	18.262	0.000
3-1 T2	anti-miR-10b	18.084	0.000
3-2 T1	anti-miR-10b	17.941	0.000
3-2 T1	anti-miR-10b	17.928	0.000
3-2 T2	anti-miR-10b	17.889	0.000
3-2 T2	anti-miR-10b	17.865	0.000
3-3 T1	anti-miR-10b	17.646	0.000
3-3 T1	anti-miR-10b	17.609	0.000

MBNL1	MBNL2	MBNL3	SART3	RSRC1	PTBP2	DGCR14	SRSF11
1.215	1.448499661	1.281362	1.100018	1.187488	1.101595151	1.093675	1.149561
0.023471	0.033819968	0.041382	0.034637	0.043142	0.033137098	0.033562	0.019881

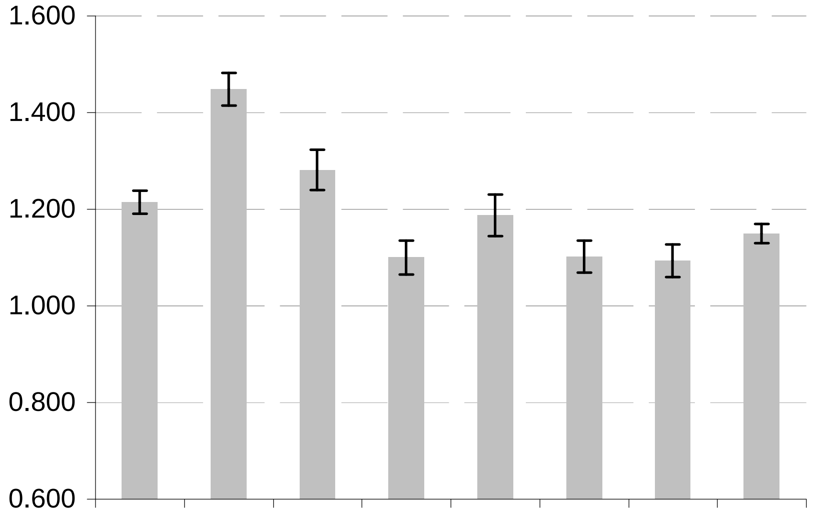

Figure 6 Panel F Source Data

DGCR14

23.589	7.92507E-08	0.678704351	1.102317	0.3609923	0.068221
24	5.96046E-08	0.505525555			
23.545	8.1705E-08	0.791058251		**p**	
23.651	7.59171E-08	0.723897574		0.090414	
23.657	7.5602E-08	1.28063331			
23.744	7.11777E-08	1.193216846			
23.525	8.28456E-08	1.067219515			
23.439	8.79343E-08	1.131202008			
24.158	5.34216E-08	0.853140402			
24.246	5.02605E-08	0.880781777			
24.209	5.15662E-08	1.023745767			
24.234	5.06803E-08	1.07092401			
23.975	6.06465E-08	1.021617625			
23.862	6.55877E-08	1.108690943			
25.305	2.41233E-08	1.766454067			
25.29	2.43754E-08	1.905085158			
24.469	4.30623E-08	1.72292146			
26.285	1.223E-08	0.511872207			
24.849	3.30907E-08	1.742134219			
25.106	2.76911E-08	1.415051572			
24.166	5.31262E-08	1.260378215			
24.173	5.28691E-08	1.177604626			
24.38	4.58025E-08	1.071667584			
24.462	4.32717E-08	1.070182366			
23.472	8.59457E-08	0.932293007			
23.635	7.67637E-08	0.805444348			
23.833	6.69194E-08	0.854916457			
23.898	6.39713E-08	1.29850898			
23.645	7.62335E-08	1.234439419	1.205576	0.2026382	0.036997
23.57	8.03014E-08	1.335015884	1.094		0.034
24.036	5.81357E-08	1.383054015			
24.105	5.54207E-08	1.177606183			
25.249	2.5078E-08	1.819895363			
25.405	2.25078E-08	1.689803103			
24.222	5.11036E-08	1.127287512			
24.084	5.62333E-08	1.21913373			
24.447	4.3724E-08	1.269143363			
24.692	3.6895E-08	1.055448515			
23.682	7.43033E-08	0.946619546			
23.565	8.05801E-08	1.037316815			
24.802	3.41865E-08	1.248206634			
24.895	3.20522E-08	1.277086127			
23.969	6.08993E-08	1.128070221			
23.978	6.05205E-08	1.074641363			
24.317	4.78469E-08	1.214073588			
24.361	4.64096E-08	1.404305539			
24.69	3.69462E-08	1.37159682			
24.514	4.17398E-08	1.315724103			

24.144	5.39426E-08	1.157375919

Figure 6 Panel F Source Data

23.962	6.11955E-08	1.335015884
24.728	3.59857E-08	1.131200513
24.519	4.15954E-08	1.155771919
24.489	4.24694E-08	1.068699208
24.683	3.71259E-08	0.925853042
24.395	4.53287E-08	1.100268532
24.478	4.27945E-08	1.021617625
24.456	4.34521E-08	0.891221983
24.18	5.26132E-08	1.051796162

Figure 6 Panel F Source Data

PTBP2

21.955	2.45972E-07	2.106512	4.241166	1.06175	0.200652
21.997	2.38915E-07	2.026312			
21.650	3.03879E-07	2.94212		**p**	
21.608	3.12856E-07	2.983192		0.040401	
21.956	2.45802E-07	4.163676		*	
22.126	2.18479E-07	3.66257			
21.661	3.01571E-07	3.884846			
21.780	2.77694E-07	3.572305			
21.697	2.94139E-07	4.697377			
21.886	2.58023E-07	4.521674			
21.861	2.62533E-07	5.212072			
22.074	2.26498E-07	4.786118			
21.782	2.7731E-07	4.671404			
21.742	2.85106E-07	4.819409			
23.428	8.86073E-08	6.488374			
23.610	7.81055E-08	6.104427			
22.912	1.26707E-07	5.069545			
23.362	9.2755E-08	3.88215			
23.234	1.01361E-07	5.336365			
23.326	9.50986E-08	4.85966			
**22.181**	2.10307E-07	4.989369			
22.242	2.016E-07	4.490434			
22.345	1.87709E-07	4.391937			
22.466	1.72608E-07	4.268878			
21.465	3.45455E-07	3.74731			
21.543	3.27274E-07	3.433923			
22.102	2.22144E-07	2.837964			
22.011	2.36608E-07	4.802738			
22.140	2.1637E-07	3.503646	4.672048	0.769769	0.14054
22.014	2.36116E-07	3.925446	1.102		0.033
22.522	1.66036E-07	3.950013			
22.534	1.64661E-07	3.498791			
23.573	8.01346E-08	5.81531			
23.496	8.45277E-08	6.346035			
22.086	2.24622E-07	4.954897			
22.179	2.10599E-07	4.56576			
22.755	1.41274E-07	4.100661			
22.679	1.48916E-07	4.260004			
21.722	2.89086E-07	3.682934			
21.615	3.11341E-07	4.00793			
22.729	1.43843E-07	5.251963			
22.746	1.42158E-07	5.664136			
21.796	2.74632E-07	5.087149			
21.838	2.66752E-07	4.736616			
22.497	1.68938E-07	4.286665			
22.265	1.98412E-07	6.003723			
22.590	1.58392E-07	5.880166			
22.712	1.45548E-07	4.587973			

22.099	2.22607E-07	4.776175

Figure 6 Panel F Source Data

21.938	2.48888E-07	5.429642
22.495	1.69173E-07	5.317892
22.527	1.65462E-07	4.597519
22.403	1.80312E-07	4.53737
22.432	1.76724E-07	4.407182
22.350	1.87059E-07	4.540514
22.311	1.92185E-07	4.587967
22.415	1.78818E-07	3.667648
22.186	2.09579E-07	4.189722

Figure 6 Panel F Source Data

SART3

22.154	2.14E-07	1.835097	3.513817	1.057323	0.199815
22.387	1.82E-07	1.546339			
22.042	2.32E-07	2.242109		**p**	
21.850	2.65E-07	2.522503		0.066438	
22.146	2.15E-07	3.6499			
22.186	2.1E-07	3.513369			
21.980	2.42E-07	3.11419			
21.841	2.66E-07	3.424412			
22.331	1.9E-07	3.026933			
22.335	1.89E-07	3.312356			
22.111	2.21E-07	4.382813			
22.308	1.93E-07	4.069509			
22.068	2.27E-07	3.831356			
22.149	2.15E-07	3.634751			
23.689	7.39E-08	5.41461			
23.771	6.99E-08	5.459832			
23.317	9.57E-08	3.828707			
23.661	7.54E-08	3.155473			
22.982	1.21E-07	6.354842			
23.612	7.8E-08	3.985763			
**22.500**	1.69E-07	3.999604			
22.425	1.78E-07	3.95549			
22.694	1.47E-07	3.448231			
22.706	1.46E-07	3.614653			
21.992	2.4E-07	2.600619			
21.976	2.42E-07	2.543574			
22.307	1.93E-07	2.462044			
22.485	1.7E-07	3.457804			
22.140	2.16E-07	3.503646	3.865262	0.666619	0.121707
22.150	2.15E-07	3.572309	1.100		0.035
22.413	1.79E-07	4.26001			
22.609	1.56E-07	3.321552			
24.024	5.86E-08	4.254106			
23.520	8.31E-08	6.241338			
22.373	1.84E-07	4.06106			
22.447	1.75E-07	3.791728			
22.977	1.21E-07	3.515808			
22.989	1.2E-07	3.436298			
21.956	2.46E-07	3.131506			
21.963	2.45E-07	3.148921			
23.014	1.18E-07	4.310505			
22.976	1.21E-07	4.829442			
22.266	1.98E-07	3.672738			
22.481	1.71E-07	3.03323			
22.615	1.56E-07	3.950008			
22.580	1.59E-07	4.826097			
22.959	1.23E-07	4.553126			
23.021	1.17E-07	3.703417			

22.522	1.66E-07	3.562419

Figure 6 Panel F Source Data

22.319	1.91E-07	4.169448
22.924	1.26E-07	3.950008
22.570	1.61E-07	4.462512
22.768	1.4E-07	3.523127
22.817	1.35E-07	3.374927
22.646	1.52E-07	3.698285
22.742	1.43E-07	3.403114
22.478	1.71E-07	3.510935
22.581	1.59E-07	3.186242

Figure 6 Panel F Source Data

SRSF11

21.263	3.97E-07	3.403	7.080968	1.721437	0.3253209
21.273	3.95E-07	3.347			
20.970	4.87E-07	4.714		**p**	
20.859	5.26E-07	5.014		0.001705	
21.186	4.19E-07	7.100		**	
21.186	4.19E-07	7.027			
20.728	5.76E-07	7.417			
20.956	4.92E-07	6.324			
21.260	3.98E-07	6.359			
21.266	3.97E-07	6.949			
21.356	3.73E-07	7.397			
21.360	3.72E-07	7.851			
20.847	5.3E-07	8.931			
20.898	5.12E-07	8.651			
22.867	1.31E-07	9.572			
22.788	1.38E-07	10.792			
22.329	1.9E-07	7.594			
22.232	2.03E-07	8.496			
22.757	1.41E-07	7.427			
22.781	1.39E-07	7.090			
21.371	3.69E-07	8.747			
**21.457**	3.47E-07	7.737			
21.628	3.09E-07	7.219			
21.473	3.44E-07	8.496			
20.851	5.29E-07	5.735			
20.711	5.83E-07	6.113			
21.270	3.95E-07	5.052			
21.328	3.8E-07	7.711			
21.031	4.67E-07	7.557	8.140002	0.771061	0.1407759
21.078	4.52E-07	7.510	1.150		0.020
21.478	3.42E-07	8.145			
21.327	3.8E-07	8.077			
23.175	1.06E-07	7.663			
23.168	1.06E-07	7.966			
21.485	3.41E-07	7.515			
21.426	3.55E-07	7.695			
21.886	2.58E-07	7.489			
21.946	2.48E-07	7.081			
20.725	5.77E-07	7.351			
20.755	5.65E-07	7.275			
22.033	2.33E-07	8.508			
22.040	2.32E-07	9.240			
21.212	4.12E-07	7.626			
21.139	4.33E-07	7.689			
21.433	3.53E-07	8.962			
21.492	3.39E-07	10.259			
21.919	2.52E-07	9.362			
21.960	2.45E-07	7.727			

21.283	3.92E-07	8.409

Figure 6 Panel F Source Data

21.418	3.57E-07	7.786
21.648	3.04E-07	9.566
21.665	3.01E-07	8.356
21.658	3.02E-07	7.605
21.640	3.06E-07	7.631
21.496	3.38E-07	8.207
21.307	3.85E-07	9.201
21.203	4.14E-07	8.496
21.209	4.13E-07	8.247

RSRC1

20.003	9.52E-07	8.150314	17.96818	5.845091	1.104618
19.856	1.05E-06	8.937399			
19.590	1.27E-06	12.26823		**p**	
19.622	1.24E-06	11.81753		0.007240	
20.057	9.17E-07	15.52862		**	
19.954	9.85E-07	16.50527			
19.801	1.09E-06	14.10227			
19.651	1.21E-06	15.62578			
19.864	1.05E-06	16.73569			
19.896	1.02E-06	17.96176			
19.921	1.01E-06	19.99902			
19.788	1.1E-06	23.34198			
19.708	1.17E-06	19.66905			
19.744	1.14E-06	19.25094			
21.239	4.04E-07	29.58628			
21.314	3.84E-07	29.97852			
20.602	6.28E-07	25.13904			
20.527	6.62E-07	27.70079			
21.137	4.34E-07	22.82998			
21.093	4.47E-07	22.84577			
20.246	8.04E-07	19.07825			
**20.150**	8.59E-07	19.14447			
20.394	7.26E-07	16.98109			
20.288	7.81E-07	19.31777			
19.677	1.19E-06	12.9408			
19.650	1.22E-06	12.75382			
20.329	7.59E-07	9.699128			
20.347	7.5E-07	15.21958			
19.610	1.25E-06	20.23605	21.33699	4.24588	0.775188
19.810	1.09E-06	18.0867	1.187		0.043
20.211	8.24E-07	19.60101			
19.800	1.1E-06	23.27739			
21.446	3.5E-07	25.40177			
21.464	3.46E-07	25.95346			
19.789	1.1E-06	24.3501			
19.778	1.11E-06	24.11495			
20.783	5.54E-07	16.08735			
20.818	5.41E-07	15.47486			
19.965	9.77E-07	12.44812			
19.941	9.93E-07	12.78923			
20.707	5.84E-07	21.33065			
20.558	6.48E-07	25.80995			
19.955	9.84E-07	18.22513			
19.831	1.07E-06	19.03861			
20.007	9.49E-07	24.08153			
19.875	1.04E-06	31.46895			
20.514	6.68E-07	24.79293			
20.581	6.38E-07	20.09629			

19.904	1.02E-06	21.86964

Figure 6 Panel F Source Data

19.812	1.09E-06	23.70068
20.159	8.54E-07	26.85009
20.067	9.1E-07	25.29633
20.442	7.02E-07	17.66543
20.343	7.52E-07	18.75048
20.065	9.12E-07	22.12883
20.108	8.85E-07	21.12466
20.011	9.46E-07	19.41172
19.885	1.03E-06	20.64693

Figure 6 Panel F Source Data

MBNL1

22.008	2.37E-07	2.03053	3.96159	0.915578	0.173028
22.191	2.09E-07	1.771358			
21.597	3.15E-07	3.052213		**p**	
21.478	3.42E-07	3.264486		0.000024	
21.892	2.57E-07	4.352538		***	
21.784	2.77E-07	4.64235			
21.477	3.43E-07	4.413298			
21.522	3.32E-07	4.271836			
21.738	2.86E-07	4.56576			
21.884	2.58E-07	4.527944			
22.026	2.34E-07	4.648799			
22.115	2.2E-07	4.652015			
21.886	2.58E-07	4.346506			
21.800	2.74E-07	4.629504			
23.936	6.23E-08	4.562599			
23.839	6.66E-08	5.208456			
22.942	1.24E-07	4.965219			
22.984	1.21E-07	5.045007			
23.568	8.04E-08	4.233515			
23.558	8.1E-08	4.137774			
22.357	1.86E-07	4.416357			
**22.394**	1.81E-07	4.041404			
22.674	1.49E-07	3.496368			
22.663	1.51E-07	3.724009			
21.800	2.74E-07	2.97081			
21.689	2.96E-07	3.103418			
22.479	1.71E-07	2.185336			
22.401	1.81E-07	3.665113			
21.674	2.99E-07	4.839496	4.81143	0.509296	0.092984
21.653	3.03E-07	5.041513	1.215		0.023
22.070	2.27E-07	5.403361			
21.997	2.39E-07	5.07658			
23.769	7E-08	5.07658			
23.910	6.34E-08	4.762952			
22.119	2.2E-07	4.842849			
22.030	2.34E-07	5.062519			
22.741	1.43E-07	4.140647			
22.698	1.47E-07	4.204271			
21.803	2.73E-07	3.481855			
21.694	2.95E-07	3.794361			
22.892	1.28E-07	4.690874			
22.990	1.2E-07	4.782803			
21.928	2.51E-07	4.642356			
21.934	2.5E-07	4.431687			
22.359	1.86E-07	4.716956			
22.261	1.99E-07	6.020391			
22.632	1.54E-07	5.71145			
22.735	1.43E-07	4.515408			

21.947	2.47E-07	5.306851

Figure 6 Panel F Source Data

22.039	2.32E-07	5.062525
22.532	1.65E-07	5.183246
22.527	1.65E-07	4.597519
22.300	1.94E-07	4.873158
22.321	1.91E-07	4.759653
22.142	2.16E-07	5.244684
22.267	1.98E-07	4.730051
22.055	2.3E-07	4.707156
22.039	2.32E-07	4.639135

Figure 6 Panel F Source Data

MBNL2

24.346	4.69E-08	0.401607	0.817803	0.277083	0.052364
24.295	4.86E-08	0.41204			
23.996	5.98E-08	0.578687		**p**	
24.126	5.46E-08	0.52082		0.000000	
24.133	5.44E-08	0.920735		***	
24.005	5.94E-08	0.995751			
23.890	6.43E-08	0.828663			
23.749	7.09E-08	0.912474			
24.105	5.54E-08	0.885066			
24.013	5.91E-08	1.035162			
24.292	4.87E-08	0.96651			
24.062	5.71E-08	1.206523			
23.782	6.93E-08	1.167851			
23.734	7.17E-08	1.211553			
25.940	1.55E-08	1.137492			
25.879	1.62E-08	1.266508			
25.095	2.79E-08	1.116403			
25.093	2.79E-08	1.16947			
26.219	1.28E-08	0.674017			
26.231	1.27E-08	0.648804			
25.034	2.91E-08	0.690569			
**24.861**	3.28E-08	0.730956			
25.000	2.98E-08	0.697303			
25.034	2.91E-08	0.719894			
24.342	4.7E-08	0.510102			
24.456	4.35E-08	0.455922			
24.844	3.32E-08	0.424212			
24.980	3.02E-08	0.613382			
23.877	6.49E-08	1.051068	1.184587	0.151489	0.027658
23.847	6.63E-08	1.101796	1.448		0.034
24.411	4.48E-08	1.06648			
23.926	6.27E-08	1.333166			
25.884	1.61E-08	1.171905			
25.840	1.66E-08	1.249937			
24.332	4.74E-08	1.044531			
24.134	5.43E-08	1.177605			
24.793	3.44E-08	0.998515			
24.822	3.37E-08	0.964501			
23.568	8.04E-08	1.024454			
23.600	7.86E-08	1.012454			
25.085	2.81E-08	1.025877			
25.061	2.86E-08	1.13828			
23.678	7.45E-08	1.380181			
23.703	7.32E-08	1.300311			
24.301	4.84E-08	1.227612			
24.189	5.23E-08	1.582119			
24.603	3.92E-08	1.456854			
24.625	3.86E-08	1.218289			

24.257	4.99E-08	1.070182

Figure 6 Panel F Source Data

24.028	5.85E-08	1.275318
24.459	4.34E-08	1.363066
24.396	4.53E-08	1.258631
24.502	4.21E-08	1.059112
24.372	4.61E-08	1.148584
24.430	4.42E-08	1.073897
24.331	4.74E-08	1.131202
23.937	6.23E-08	1.277086
23.815	6.78E-08	1.354589

Figure 6 Panel F Source Data

MBNL3

23.234	1.01E-07	0.868054	1.84525	0.555675	0.105013
23.227	1.02E-07	0.863852			
22.939	1.24E-07	1.204018		**p**	
22.821	1.35E-07	1.286862		0.000083	
22.955	1.23E-07	2.083281		***	
22.939	1.24E-07	2.084724			
22.707	1.46E-07	1.881464			
22.862	1.31E-07	1.687463			
23.028	1.17E-07	1.867174			
22.978	1.21E-07	2.121164			
23.043	1.16E-07	2.29717			
22.985	1.2E-07	2.545334			
22.950	1.23E-07	2.07895			
22.978	1.21E-07	2.046071			
24.752	3.54E-08	2.591623			
24.725	3.61E-08	2.81836			
23.917	6.31E-08	2.526004			
23.682	7.43E-08	3.109874			
24.919	3.15E-08	1.659624			
24.957	3.07E-08	1.56901			
23.622	7.75E-08	1.837644			
**23.672**	7.48E-08	1.666539			
23.865	6.55E-08	1.531406			
23.903	6.37E-08	1.576644			
22.917	1.26E-07	1.369696			
22.796	1.37E-07	1.440787			
23.347	9.37E-08	1.197359			
23.382	9.15E-08	1.856849			
22.783	1.39E-07	2.243662	2.364433	0.41824	0.07636
22.744	1.42E-07	2.366668	1.281		0.041
23.213	1.03E-07	2.446734			
23.183	1.05E-07	2.231256			
24.893	3.21E-08	2.329235			
24.836	3.34E-08	2.506815			
23.276	9.85E-08	2.171748			
23.166	1.06E-07	2.303545			
23.429	8.85E-08	2.570154			
23.951	6.17E-08	1.764006			
22.873	1.3E-07	1.658474			
22.843	1.33E-07	1.71102			
24.128	5.45E-08	1.991502			
24.026	5.85E-08	2.332468			
22.999	1.19E-07	2.209709			
22.958	1.23E-07	2.179286			
23.114	1.1E-07	2.795013			
23.103	1.11E-07	3.358591			
23.568	8.04E-08	2.985259			
23.453	8.71E-08	2.745098			

22.966	1.22E-07	2.618711

Figure 6 Panel F Source Data

22.984	1.21E-07	2.629626
23.203	1.04E-07	3.255448
23.226	1.02E-07	2.832068
23.687	7.4E-08	1.863295
23.610	7.81E-08	1.947813
23.338	9.43E-08	2.289221
23.229	1.02E-07	2.428147
23.210	1.03E-07	2.113827
23.214	1.03E-07	2.054596

Figure 6 Panel G Source Data

Sample Name		GAPDH		miR125b	
4-1 T1	contol	16.386	0.000	23.596	7.88671E-08
4-1 T1	contol	16.372	0.000	23.461	8.66034E-08
4-1 T2	contol	16.563	0.000	22.151	2.14726E-07
4-1 T2	contol	16.541	0.000	22.191	2.08854E-07
4-2 T1	contol	17.370	0.000	21.948	2.47169E-07
4-2 T1	contol	17.355	0.000	22.022	2.34811E-07
4-2 T2	contol	16.975	0.000	22.859	1.31448E-07
4-2 T2	contol	16.973	0.000	22.919	1.26094E-07
4-4 T	contol	17.285	0.000	20.939	4.97431E-07
4-4 T	contol	17.419	0.000	20.864	5.23974E-07
4-4 T1	contol	17.599	0.000	21.437	3.52225E-07
4-4 T1	contol	17.689	0.000	21.495	3.38345E-07
5-1 T1	contol	17.362	0.000	20.792	5.50788E-07
5-1 T1	contol	17.367	0.000	20.864	5.23974E-07
5-1 T2	contol	19.482	0.000	22.522	1.66036E-07
5-1 T2	contol	19.576	0.000	22.555	1.62281E-07
5-2 T2	contol	18.610	0.000	21.789	2.75967E-07
5-2 T2	contol	18.675	0.000	21.785	2.76733E-07
5-2 T3	contol	19.006	0.000	22.129	2.18026E-07
5-2 T3	contol	18.963	0.000	21.901	2.55354E-07
5-4 T1	contol	17.856	0.000	21.830	2.68235E-07
5-4 T1	contol	17.765	0.000	21.830	2.68235E-07
5-4 T2	contol	17.836	0.000	21.507	3.35543E-07
5-4 T2	contol	17.916	0.000	21.469	3.44498E-07
5-5 T2	contol	16.727	0.000	21.626	3.08977E-07
5-5 T2	contol	16.679	0.000	21.340	3.76721E-07
5-5 T3	contol	16.963	0.000	20.645	6.09868E-07
5-5 T3	contol	17.631	0.000	21.704	2.92715E-07
1-2 T1	anti-miR-10b	17.305	0.000	21.492	3.3905E-07
1-2 T1	anti-miR-10b	17.343	0.000	21.643	3.05357E-07
1-2 T2	anti-miR-10b	17.860	0.000	21.074	4.52996E-07
1-2 T2	anti-miR-10b	17.697	0.000	20.238	8.08639E-07
1-3 T1	anti-miR-10b	19.469	0.000	22.246	2.01042E-07
1-3 T1	anti-miR-10b	19.518	0.000	22.160	2.13391E-07
1-3 T2	anti-miR-10b	17.751	0.000	21.006	4.74858E-07
1-3 T2	anti-miR-10b	17.726	0.000	21.004	4.75517E-07
2-1 T1	anti-miR-10b	18.147	0.000	20.769	5.59639E-07
2-1 T1	anti-miR-10b	18.126	0.000	20.604	6.27448E-07
2-1 T2	anti-miR-10b	16.959	0.000	21.700	2.93528E-07
2-1 T2	anti-miR-10b	16.974	0.000	21.762	2.81181E-07
2-3 T1	anti-miR-10b	18.478	0.000	20.949	4.93995E-07
2-3 T1	anti-miR-10b	18.604	0.000	20.772	5.58477E-07
2-3 T2	anti-miR-10b	17.499	0.000	21.232	4.06005E-07
2-3 T2	anti-miR-10b	17.438	0.000	21.295	3.88657E-07
2-4 T1	anti-miR-10b	17.953	0.000	22.004	2.37759E-07
2-4 T1	anti-miR-10b	18.207	0.000	22.170	2.11917E-07
2-4 T2	anti-miR-10b	18.502	0.000	22.100	2.22452E-07
2-4 T2	anti-miR-10b	18.266	0.000	22.232	2.03002E-07

3-1 T1	anti-miR-10b	17.711	0.000	20.844	5.31289E-07

Figure 6 Panel G Source Data

3-1 T1	anti-miR-10b	17.735	0.000	20.920	5.04025E-07
3-1 T2	anti-miR-10b	18.262	0.000	21.842	2.66013E-07
3-1 T2	anti-miR-10b	18.084	0.000	21.864	2.61987E-07
3-2 T1	anti-miR-10b	17.941	0.000	19.920	1.00805E-06
3-2 T1	anti-miR-10b	17.928	0.000	19.918	1.00945E-06
3-2 T2	anti-miR-10b	17.889	0.000	20.311	7.6874E-07
3-2 T2	anti-miR-10b	17.865	0.000	20.384	7.3081E-07
3-3 T1	anti-miR-10b	17.646	0.000	20.693	5.8991E-07
3-3 T1	anti-miR-10b	17.609	0.000	20.467	6.89953E-07

	SRSF11	RSRC1	MBNL2	miR10b
control 4-1	4.119352017	10.29337	0.478288	14.34426
control 4-1	4.863662623	12.04288	0.549754	11.63606
control 4-2	6.96702676	15.44049	0.914406	9.016106
control 4-2	6.870593514	14.86403	0.870568	12.4613
control 4-4	7.138985415	19.50961	1.023315	4.469057
control 4-4	7.62372556	21.6705	1.086517	5.050545
control 5-1	9.486531435	24.6212	1.195851	3.147187
control 5-1	10.18199062	29.7824	1.202	1.397372
control 5-2	7.652035185	24.62889	0.902173	1.924839
control 5-2	7.258870058	22.83787	0.66141	1.354958
control 5-4	8.050141433	18.6304	0.70968	3.070239
control 5-4	7.857859127	18.14943	0.708598	2.473571
control 5-5	6.152706095	12.65333	0.500904	5.387452
control 5-5	6.381339422	12.45935	0.518797	3.773279
miR-10b-I	7.822333829	20.30029	1.138127	4.242441
miR-10b-I	8.110939701	21.4392	1.199823	2.311522
miR-10b-I	7.709700626	24.95507	1.160994	2.662093
miR-10b-I	7.605037639	24.23253	1.111068	4.072336
miR-10b-I	7.298767943	14.19989	0.999981	3.199198
miR-10b-I	7.312558135	12.61867	1.018454	5.396195
miR-10b-I	8.265730693	21.10109	1.211162	3.941335
miR-10b-I	7.657475712	18.63187	1.340246	5.859976
miR-10b-I	9.077617202	25.10993	1.371219	4.603188
miR-10b-I	8.544493158	22.44461	1.337572	3.476506
miR-10b-I	8.529062044	24.42918	1.241799	3.171467
miR-10b-I	8.960919981	26.07321	1.310849	3.153277
miR-10b-I	8.160963118	19.91735	1.103199	2.479189
miR-10b-I	8.704213263	21.62675	1.10255	2.870638

SRSF11

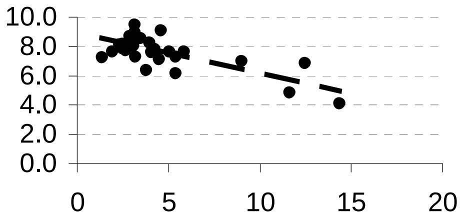

RSRC1

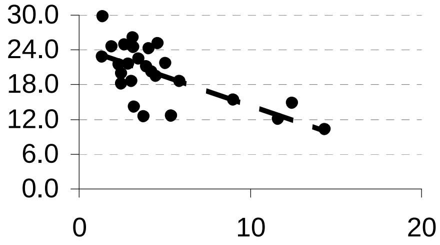

MBNL2

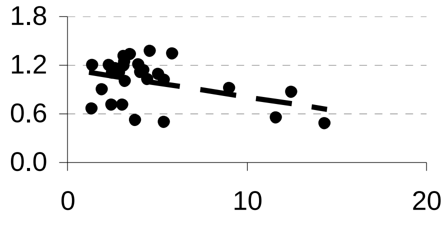

Figure 6 Panel G Source Data

SRSF11

21.263	3.97374E-07	3.403	7.080968	1.721437	0.325321
21.273	3.94629E-07	3.347			
20.970	4.86857E-07	4.714		**p**	
20.859	5.25794E-07	5.014		0.001705	
21.186	4.19158E-07	7.100		**	
21.186	4.19158E-07	7.027			
20.728	5.75771E-07	7.417			
20.956	4.91604E-07	6.324			
21.260	3.98201E-07	6.359			
21.266	3.96548E-07	6.949			
21.356	3.72566E-07	7.397			
21.360	3.71534E-07	7.851			
20.847	5.30185E-07	8.931			
20.898	5.1177E-07	8.651			
22.867	1.30721E-07	9.572			
22.788	1.38079E-07	10.792			
22.329	1.89802E-07	7.594			
22.232	2.03002E-07	8.496			
22.757	1.41078E-07	7.427			
22.781	1.38751E-07	7.090			
21.371	3.68713E-07	8.747			
21.457	3.47375E-07	7.737			
21.628	3.08548E-07	7.219			
21.473	3.43545E-07	8.496			
20.851	5.28717E-07	5.735			
20.711	5.82596E-07	6.113			
21.270	3.9545E-07	5.052			
21.328	3.79868E-07	7.711			
21.031	4.667E-07	7.557	8.140002	0.771061	0.140776
21.078	4.51742E-07	7.510	1.150		0.020
21.478	3.42356E-07	8.145			
21.327	3.80131E-07	8.077			
23.175	1.05592E-07	7.663			
23.168	1.06105E-07	7.966			
21.485	3.40699E-07	7.515			
21.426	3.54921E-07	7.695			
21.886	2.58023E-07	7.489			
21.946	2.47512E-07	7.081			
20.725	5.7697E-07	7.351			
20.755	5.65097E-07	7.275			
22.033	2.33027E-07	8.508			
22.040	2.31899E-07	9.240			
21.212	4.11672E-07	7.626			
21.139	4.33039E-07	7.689			
21.433	3.53203E-07	8.962			
21.492	3.3905E-07	10.259			
21.919	2.52187E-07	9.362			
21.960	2.45122E-07	7.727			

21.283	3.91903E-07	8.409

Figure 6 Panel G Source Data

21.418	3.56894E-07	7.786
21.648	3.043E-07	9.566
21.665	3.00736E-07	8.356
21.658	3.02198E-07	7.605
21.640	3.05993E-07	7.631
21.496	3.38111E-07	8.207
21.307	3.85438E-07	9.201
21.203	4.14249E-07	8.496
21.209	4.12529E-07	8.247

Figure 6 Panel G Source Data

RSRC1

20.003	9.51693E-07	8.15031382	17.96818	5.8450912	1.1046184
19.856	1.05378E-06	8.937398981			
19.590	1.26713E-06	12.26823466		**p**	
19.622	1.23934E-06	11.81753158		0.007240	
20.057	9.1673E-07	15.5286211		**	
19.954	9.84572E-07	16.50527486			
19.801	1.09472E-06	14.10227463			
19.651	1.21467E-06	15.62578424			
19.864	1.04795E-06	16.73568523			
19.896	1.02496E-06	17.96176114			
19.921	1.00735E-06	19.99902267			
19.788	1.10463E-06	23.34198356			
19.708	1.16762E-06	19.66905198			
19.744	1.13884E-06	19.25094402			
21.239	4.0404E-07	29.58627617			
21.314	3.83572E-07	29.97851941			
20.602	6.28319E-07	25.13903764			
20.527	6.61846E-07	27.70078584			
21.137	4.3364E-07	22.82997822			
21.093	4.47069E-07	22.84576934			
20.246	8.04168E-07	19.07824818			
20.150	8.595E-07	19.14447193			
20.394	7.25763E-07	16.98109436			
20.288	7.81094E-07	19.31776721			
19.677	1.19298E-06	12.9408047			
19.650	1.21552E-06	12.75381917			
20.329	7.59209E-07	9.699128087			
20.347	7.49795E-07	15.21957942			
19.610	1.24969E-06	20.23605113	21.33699	4.2458797	0.775188
19.810	1.08792E-06	18.08669793	1.187		0.043
20.211	8.23915E-07	19.60101363			
19.800	1.09548E-06	23.27739093			
21.446	3.50035E-07	25.4017662			
21.464	3.45694E-07	25.95346282			
19.789	1.10387E-06	24.35010491			
19.778	1.11232E-06	24.11495385			
20.783	5.54234E-07	16.0873479			
20.818	5.4095E-07	15.47486423			
19.965	9.77093E-07	12.44811744			
19.941	9.93484E-07	12.78922676			
20.707	5.84214E-07	21.3306511			
20.558	6.47776E-07	25.80995492			
19.955	9.8389E-07	18.22512514			
19.831	1.0722E-06	19.03861388			
20.007	9.49058E-07	24.08153309			
19.875	1.03999E-06	31.46894531			
20.514	6.67837E-07	24.79293322			
20.581	6.37532E-07	20.0962939			

19.904	1.01929E-06	21.86963601

Figure 6 Panel G Source Data

19.812	1.08641E-06	23.70067964
20.159	8.54154E-07	26.85008822
20.067	9.10398E-07	25.2963335
20.442	7.02013E-07	17.66542727
20.343	7.51877E-07	18.75047547
20.065	9.1166E-07	22.12883499
20.108	8.84889E-07	21.12465916
20.011	9.46431E-07	19.41172495
19.885	1.03281E-06	20.64692765

Figure 6 Panel G Source Data

MBNL2

24.346	4.68947E-08	0.401607	0.817803	0.277083	0.052363708
24.295	4.85821E-08	0.41204			
23.996	5.97701E-08	0.578687		**p**	
24.126	5.46198E-08	0.52082		0.000000	
24.133	5.43555E-08	0.920735		***	
24.005	5.93985E-08	0.995751			
23.890	6.4327E-08	0.828663			
23.749	7.09314E-08	0.912474			
24.105	5.54207E-08	0.885066			
24.013	5.90699E-08	1.035162			
24.292	4.86832E-08	0.96651			
24.062	5.70974E-08	1.206523			
23.782	6.93274E-08	1.167851			
23.734	7.16728E-08	1.211553			
25.940	1.55339E-08	1.137492			
25.879	1.62048E-08	1.266508			
25.095	2.79031E-08	1.116403			
25.093	2.79418E-08	1.16947			
26.219	1.28025E-08	0.674017			
26.231	1.26964E-08	0.648804			
25.034	2.91082E-08	0.690569			
24.861	3.28166E-08	0.730956			
25.000	2.98023E-08	0.697303			
25.034	2.91082E-08	0.719894			
24.342	4.70249E-08	0.510102			
24.456	4.34521E-08	0.455922			
24.844	3.32056E-08	0.424212			
24.980	3.02184E-08	0.613382			
23.877	6.49092E-08	1.051068	1.184587	0.151489	0.027658057
23.847	6.62732E-08	1.101796	1.448		0.034
24.411	4.48288E-08	1.06648			
23.926	6.27417E-08	1.333166			
25.884	1.61488E-08	1.171905			
25.840	1.66489E-08	1.249937			
24.332	4.7352E-08	1.044531			
24.134	5.43178E-08	1.177605			
24.793	3.44004E-08	0.998515			
24.822	3.37158E-08	0.964501			
23.568	8.04127E-08	1.024454			
23.600	7.86488E-08	1.012454			
25.085	2.80972E-08	1.025877			
25.061	2.85685E-08	1.13828			
23.678	7.45095E-08	1.380181			
23.703	7.32295E-08	1.300311			
24.301	4.83804E-08	1.227612			
24.189	5.2286E-08	1.582119			
24.603	3.92427E-08	1.456854			
24.625	3.86488E-08	1.218289			

24.257	4.98787E-08	1.070182

Figure 6 Panel G Source Data

24.028	5.8459E-08	1.275318
24.459	4.33618E-08	1.363066
24.396	4.52973E-08	1.258631
24.502	4.20884E-08	1.059112
24.372	4.60571E-08	1.148584
24.430	4.42422E-08	1.073897
24.331	4.73848E-08	1.131202
23.937	6.22651E-08	1.277086
23.815	6.77595E-08	1.354589

Figure 6 Panel G Source Data

miR10b

26.160	1.33E-08	16.91061	5.908448	4.597104	0.868771
26.001	1.49E-08	17.19428			
25.232	2.54E-08	11.81752		**p**	
25.317	2.39E-08	11.45461		0.004298	
26.127	1.36E-08	5.520716		**	
26.175	1.32E-08	5.621115			
25.989	1.5E-08	11.42288			
25.808	1.7E-08	13.49971			
25.618	1.94E-08	3.903736			
25.555	2.03E-08	3.871402			
25.877	1.62E-08	4.60709			
25.681	1.86E-08	5.494001			
25.133	2.72E-08	4.934338			
25.227	2.55E-08	4.859666			
28.588	2.48E-09	1.49263			
28.818	2.11E-09	1.302114			
27.106	6.92E-09	2.508552			
27.118	6.87E-09	2.480887			
28.161	3.33E-09	1.528225			
28.304	3.02E-09	1.181692			
26.575	1E-08	3.729172			
26.624	9.67E-09	3.604641			
26.732	8.97E-09	2.673734			
26.928	7.83E-09	2.273408			
25.261	2.49E-08	8.049259			
25.410	2.24E-08	5.953989			
25.958	1.53E-08	2.51552			
26.017	1.47E-08	5.031039			
25.534	2.06E-08	6.070673	3.468341	1.664812	0.303952
25.637	1.92E-08	6.276049	0.587		0.051
26.117	1.37E-08	3.03323			
26.213	1.29E-08	1.589814			
28.508	2.62E-09	1.303018			
28.540	2.56E-09	1.200683			
25.625	1.93E-08	4.069515			
25.621	1.94E-08	4.075157			
27.459	5.42E-09	0.968521			
27.197	6.5E-09	1.035879			
25.969	1.52E-08	5.186844			
25.919	1.58E-08	5.605545			
26.504	1.05E-08	2.127053			
26.476	1.07E-08	1.918336			
25.437	2.2E-08	5.422117			
25.284	2.45E-08	6.297835			
26.150	1.34E-08	5.648455			
26.275	1.23E-08	5.811283			
26.975	7.58E-09	3.407837			
27.050	7.2E-09	3.545176			

25.630	1.93E-08	3.624688

Figure 6 Panel G Source Data

26.102	1.39E-08	2.754627
26.925	7.85E-09	2.950288
26.761	8.79E-09	3.356265
25.447	2.19E-08	2.168738
25.557	2.03E-08	2.006744
25.440	2.2E-08	2.857704
25.500	2.11E-08	2.883572
25.460	2.17E-08	3.672743
25.441	2.2E-08	3.181827

SRSF11		RSRC1		MBNL2		miR10b	
3.403114	4.119352	8.150314	10.29337	0.401607	0.478288	16.91061	14.34426
3.346969		8.937399		0.41204		17.19428	
4.713689	4.863663	12.26823	12.04288	0.578687	0.549754	11.81752	11.63606
5.013636		11.81753		0.52082		11.45461	
7.100183	6.967027	15.52862	15.44049	0.920735	0.914406	5.520716	9.016106
7.026737		16.50527		0.995751		5.621115	
7.417104	6.870594	14.10227	14.86403	0.828663	0.870568	11.42288	12.4613
6.324083		15.62578		0.912474		13.49971	
6.359246	7.138985	16.73569	19.50961	0.885066	1.023315	3.903736	4.469057
6.949244		17.96176		1.035162		3.871402	
7.396569	7.623726	19.99902	21.6705	0.96651	1.086517	4.60709	5.050545
7.850882		23.34198		1.206523		5.494001	
8.93121	9.486531	19.66905	24.6212	1.167851	1.195851	4.934338	3.147187
8.650935		19.25094		1.211553		4.859666	
9.572236	10.18199	29.58628	29.7824	1.137492	1.202	1.49263	1.397372
10.79175		29.97852		1.266508		1.302114	
7.593981	7.652035	25.13904	24.62889	1.116403	0.902173	2.508552	1.924839
8.49642		27.70079		1.16947		2.480887	
7.427397	7.25887	22.82998	22.83787	0.674017	0.66141	1.528225	1.354958
7.090343		22.84577		0.648804		1.181692	
8.747415	8.050141	19.07825	18.6304	0.690569	0.70968	3.729172	3.070239
7.737432		19.14447		0.730956		3.604641	
7.219287	7.857859	16.98109	18.14943	0.697303	0.708598	2.673734	2.473571
8.496431		19.31777		0.719894		2.273408	
5.735247	6.152706	12.9408	12.65333	0.510102	0.500904	8.049259	5.387452
6.112898		12.75382		0.455922		5.953989	
5.052002	6.381339	9.699128	12.45935	0.424212	0.518797	2.51552	3.773279
7.710677		15.21958		0.613382		5.031039	
7.557224	7.822334	20.23605	20.30029	1.051068	1.138127	6.070673	4.242441
7.510232		18.0867		1.101796		6.276049	
8.14467	8.11094	19.60101	21.4392	1.06648	1.199823	3.03323	2.311522
8.07721		23.27739		1.333166		1.589814	
7.662721	7.709701	25.40177	24.95507	1.171905	1.160994	1.303018	2.662093
7.966006		25.95346		1.249937		1.200683	
7.515427	7.605038	24.3501	24.23253	1.044531	1.111068	4.069515	4.072336
7.694649		24.11495		1.177605		4.075157	
7.48943	7.298768	16.08735	14.19989	0.998515	0.999981	0.968521	3.199198
7.080526		15.47486		0.964501		1.035879	
7.350567	7.312558	12.44812	12.61867	1.024454	1.018454	5.186844	5.396195
7.27455		12.78923		1.012454		5.605545	
8.508211	8.265731	21.33065	21.10109	1.025877	1.211162	2.127053	3.941335
9.23976		25.80995		1.13828		1.918336	
7.625632	7.657476	18.22513	18.63187	1.380181	1.340246	5.422117	5.859976
7.68932		19.03861		1.300311		6.297835	
8.962211	9.077617	24.08153	25.10993	1.227612	1.371219	5.648455	4.603188
10.25927		31.46895		1.582119		5.811283	
9.362257	8.544493	24.79293	22.44461	1.456854	1.337572	3.407837	3.476506
7.726729		20.09629		1.218289		3.545176	

8.408542	8.529062	21.86964	24.42918	1.070182	1.241799	3.624688	3.171467

Figure 6 Panel G Source Data

7.785866		23.70068		1.275318		2.754627	
9.565594	8.96092	26.85009	26.07321	1.363066	1.310849	2.950288	3.153277
8.356246		25.29633		1.258631		3.356265	
7.60451	8.160963	17.66543	19.91735	1.059112	1.103199	2.168738	2.479189
7.630916		18.75048		1.148584		2.006744	
8.207005	8.704213	22.12883	21.62675	1.073897	1.10255	2.857704	2.870638
9.201421		21.12466		1.131202		2.883572	
