# Supplementary material for: Therapeutic potential of targeting microRNA‐10b in established intracranial glioblastoma: first steps toward the clinic
Source: EMBO Mol Med. 2016 Feb 10;8(3):268–87. doi: 10.15252/emmm.201505495 (PMC4772951; doi:10.15252/emmm.201505495)

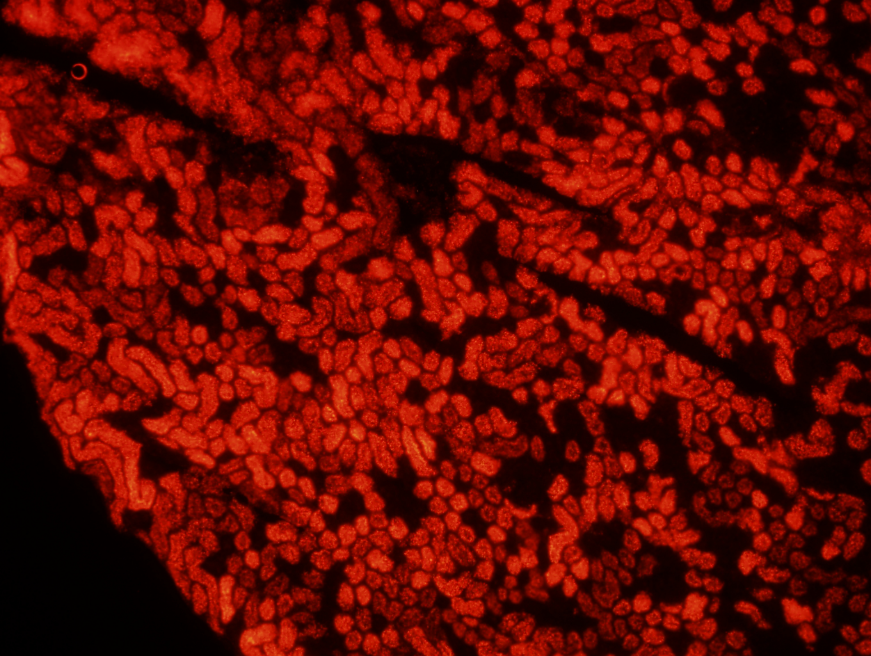

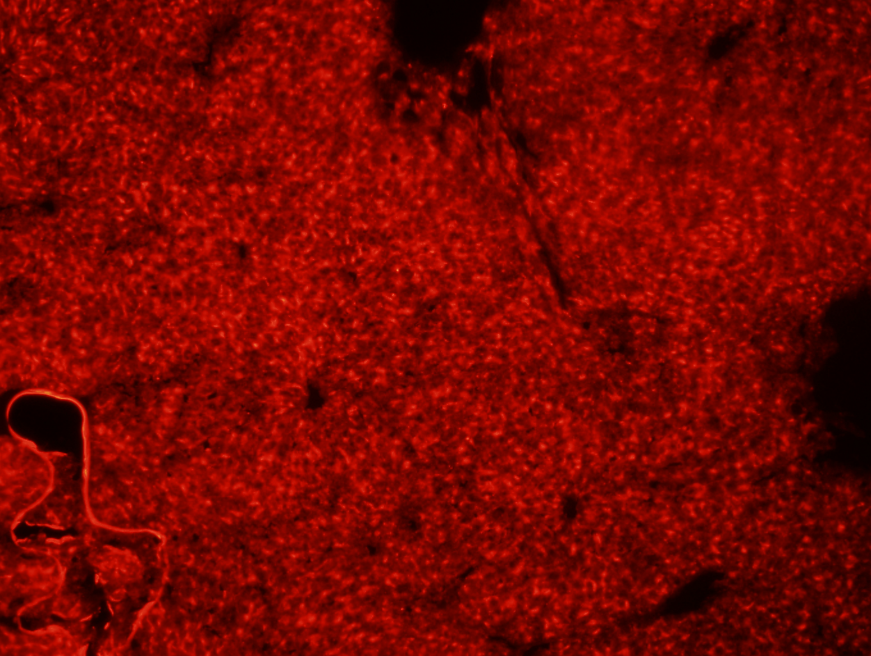

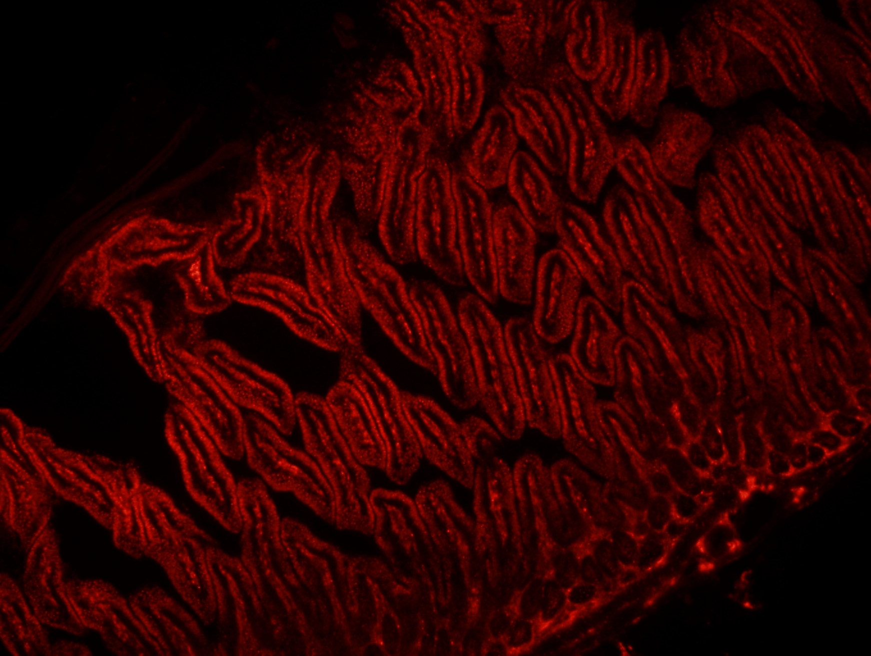

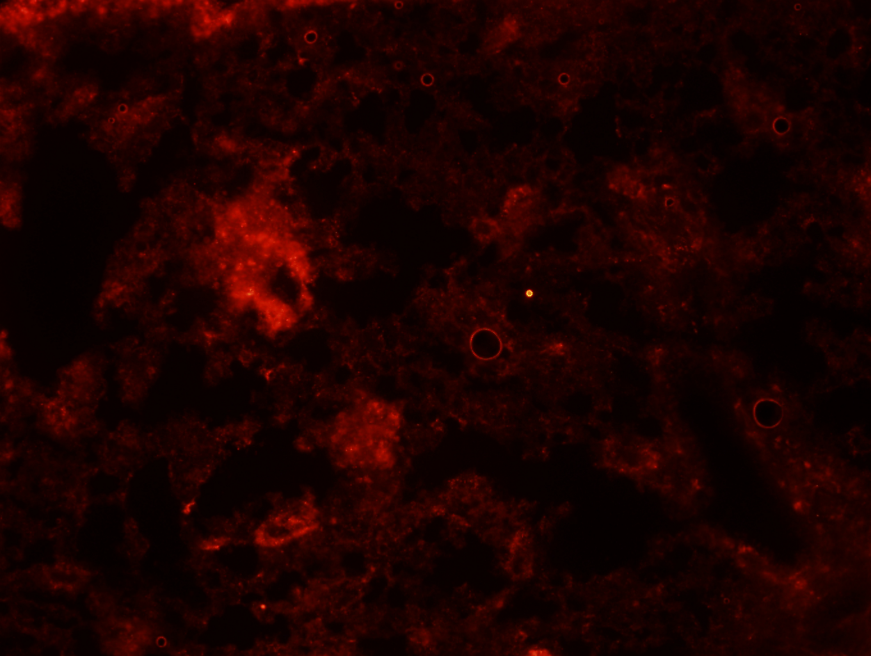

Figure 7 Panel B Source Data

| | g | 11.11.11 | 11.14.1 | 11.21.1 | 11.25.11 | 11.28.1 | 12.01.1 | 12.05. | 12.08.11 |
|---|---|---|---|---|---|---|---|---|---|
| Cage | Mouse | 7 | 10 | 17 | 21 | 24 | 27 | 31 | 34 |
| 1 | 3 | 24 | 25 | 24 | 25.5 | 25.1 | 24.9 | 25.7 | 25.5 |
| 2 | 1 | 19 | 20 | 19 | 19.7 | 19.8 | 19.5 | 19.7 | 20.5 |
| | 3 | 21 | 22 | 21 | 24.6 | 24.4 | 23.5 | 23.5 | 24.5 |
| | 4 | 17 | 18 | 17 | 20.6 | 21.3 | 21.1 | 20.9 | 21.7 |
| 3 | 1 | 22 | 23 | 22 | 23.9 | 24 | 23.9 | 23.9 | 24.6 |
| | 2 | 23 | 23 | 23 | 23.2 | 23.6 | 23.4 | 22.3 | 24.2 |
| | 3 | 20 | 20 | 20 | 24.2 | 23.6 | 23.5 | 23.7 | 24.3 |
| 4 | 1 | 21 | 23 | 21 | 22.2 | 21.9 | 22.5 | 21.7 | 20.8 |
| | 2 | 22 | 22 | 22 | 20.8 | 20.4 | 21.4 | 21.6 | 22 |
| | 4 | 24 | 25 | 24 | 23.1 | 22.8 | 22.2 | 22.4 | 22.6 |
| 5 | 1 | 21 | 22 | 21 | 22.8 | 23.1 | 24.4 | 23.9 | 23.7 |
| | 2 | 22 | 23 | 22 | 25.2 | 24 | 25 | 24.6 | 25.1 |
| | 4 | 21 | 22 | 21 | 21.9 | 22.8 | 23.6 | 23 | 23.8 |
| | 5 | 22 | 23 | 22 | 22.7 | 22 | 22.6 | 22.1 | 21.7 |
| | | 20.8571 | 21.571 | 20.857 | 23.1 | 23.114 | 22.83 | 22.81 | 23.6 |
| | | 2.4103 | 2.3705 | 2.4103 | 2.1479 | 1.8765 | 1.859 | 2.016 | 1.8 |
| | | 21.8571 | 22.857 | 21.857 | 22.671 | 22.429 | 23.1 | 22.76 | 22.8 |
| | | 1.06904 | 1.069 | 1.069 | 1.3487 | 1.1383 | 1.282 | 1.139 | 1.47 |
| | | 0.91101 | 0.8959 | 0.911 | 0.8118 | 0.7093 | 0.703 | 0.762 | 0.68 |
| | | 0.40406 | 0.4041 | 0.4041 | 0.5098 | 0.4302 | 0.485 | 0.43 | 0.56 |
| | **p** | 0.10573 | 0.0768 | 0.0931 | 0.4309 | 0.1632 | 0.4 | 0.222 | 0.11 |

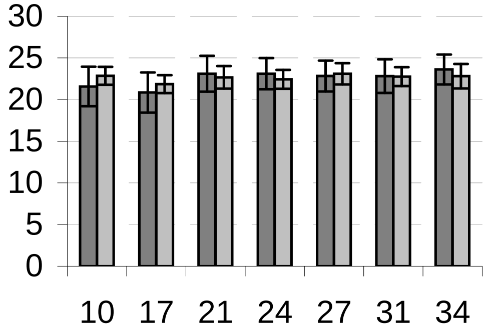

Figure 7 Panel C Source Data

| Mouse 1 | Cage 2 | Cage 3 | | | | Cage 4 | Cage 5 | | | | |
|---|---|---|---|---|---|---|---|---|---|---|---|
| mg | a-10b | a-10b | Average | SD | SE | Ctrl | Ctrl | Average | SD | SE | **p** |
| Kidney | 210 | 302 | 256 | 65.05 | 46 | 217 | 206 | 211.5 | 7.778 | 5.5 | 0.21907 |
| Spleen | 102 | 146 | 124 | 31.11 | 22 | 98 | 103 | 100.5 | 3.536 | 2.5 | 0.19987 |
| Heart | 164 | 184 | 174 | 14.14 | 10 | 142 | 156 | 149 | 9.899 | 7 | 0.08856 |
| Lung | 130 | 171 | 150.5 | 28.99 | 20.5 | 116 | 127 | 121.5 | 7.778 | 5.5 | 0.15259 |
| Liver | 390 | 557 | 473.5 | 118.1 | 83.5 | 444 | 809 | 626.5 | 258.1 | 182.5 | 0.26274 |

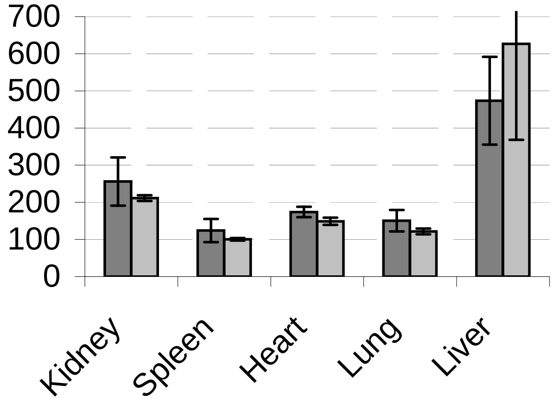

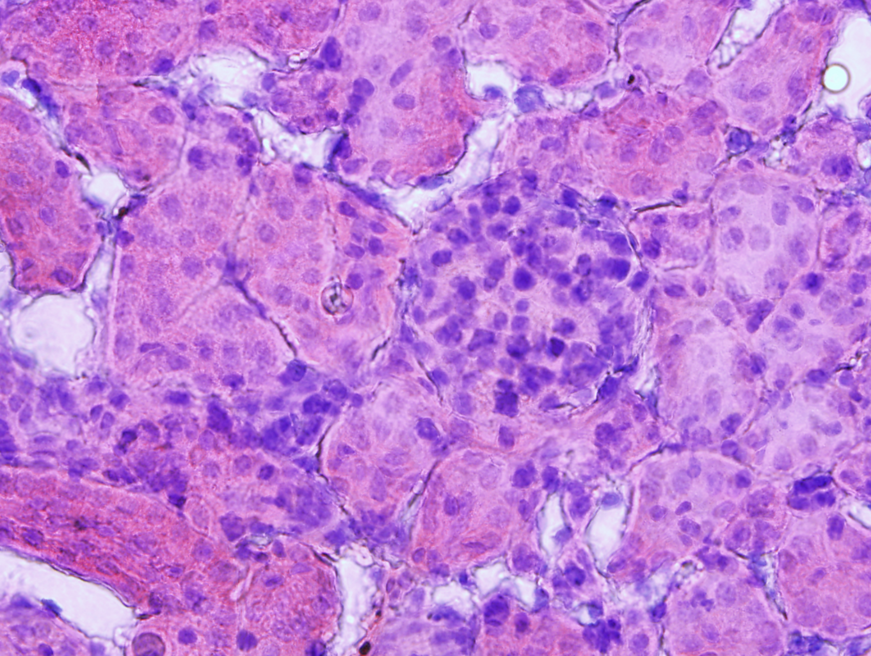

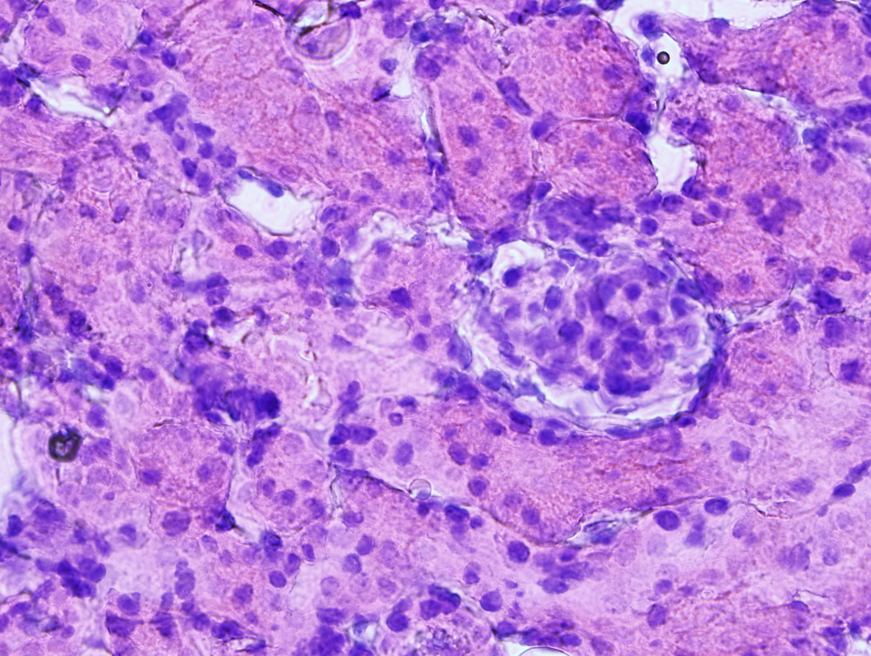

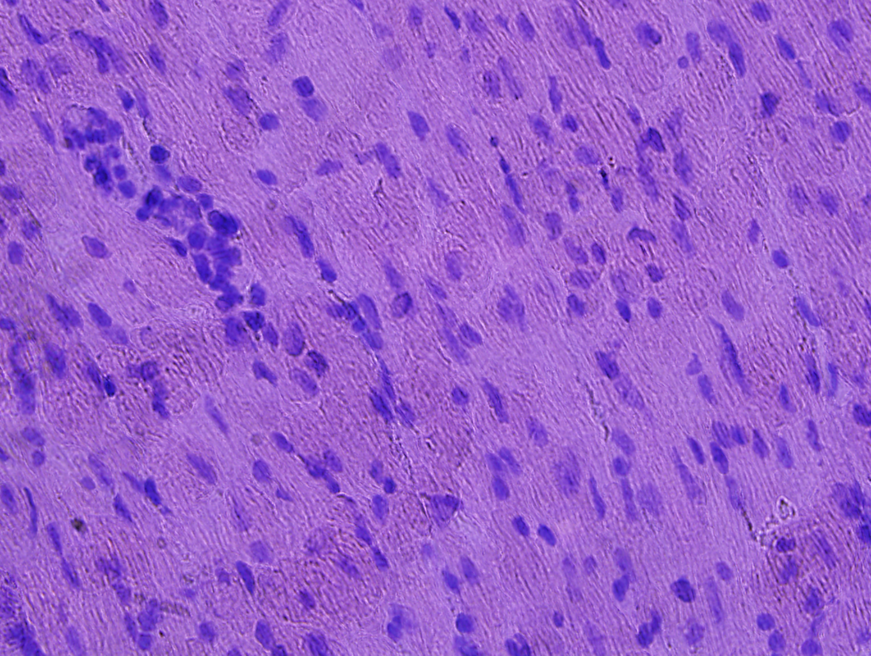

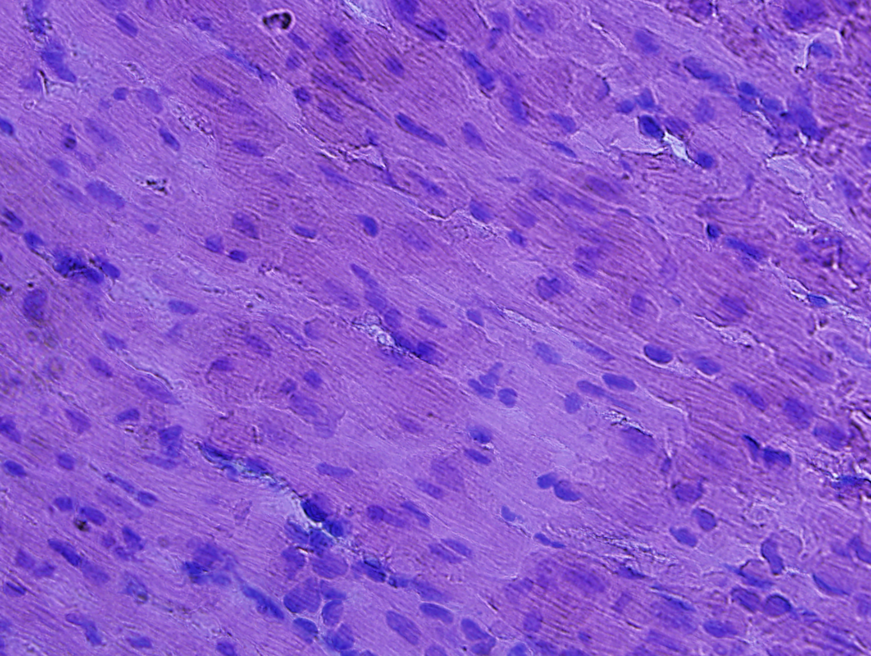

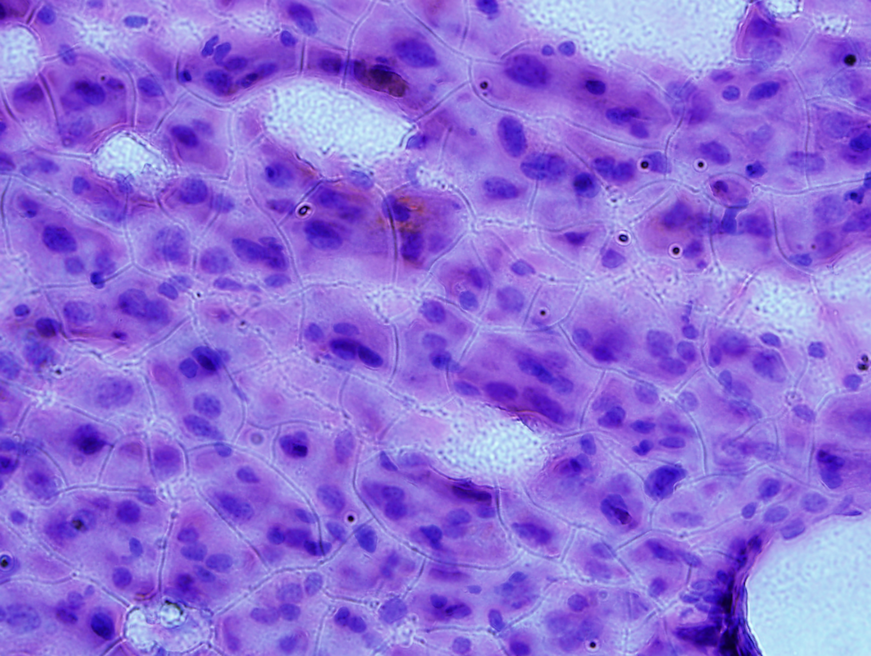

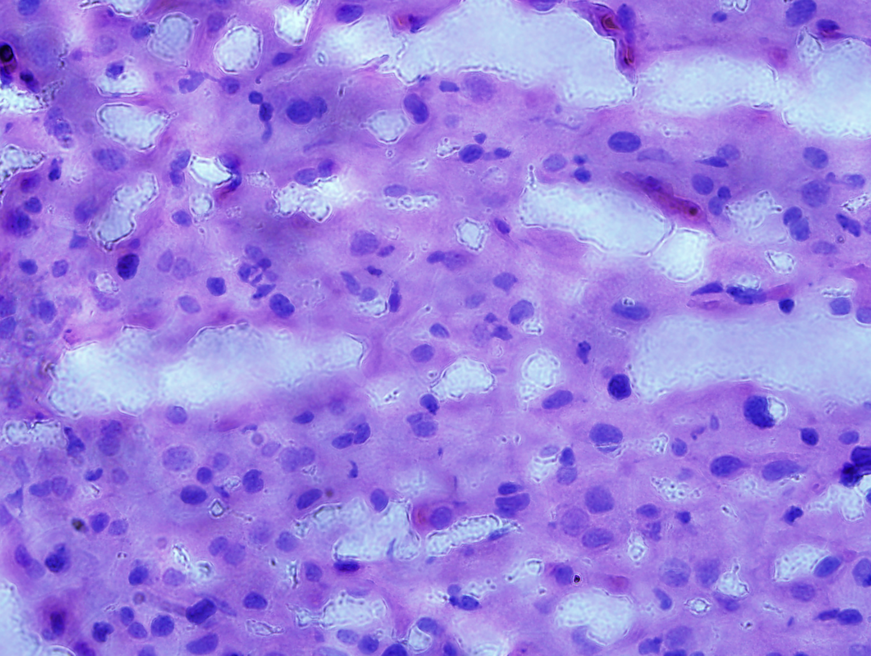

Supplement: Supplementary file 13 — Source Data for Figure 7 [file EMMM-8-268-s011.pdf]
