# Supplementary material for: Therapeutic potential of targeting microRNA‐10b in established intracranial glioblastoma: first steps toward the clinic
Source: EMBO Mol Med. 2016 Feb 10;8(3):268–87. doi: 10.15252/emmm.201505495 (PMC4772951; doi:10.15252/emmm.201505495)

Figure 8 Panel A Source Data

	Total Flux [p/s]		Total Flux [p/s]			
Days of treatm	-8	-2	2	5	8	13
control	3.66E+05	1.62E+06	1.80E+06	1.82E+06	7.86E+06	1.63E+07
anti-miR-10b	3.94E+05	1.31E+06	1.30E+06	1.64E+05	3.24E+06	4.54E+06
control	1.70E+05	9.38E+05	1.22E+06	2.93E+06	4.55E+06	1.48E+07
anti-miR-10b	4.54E+05	2.47E+06	3.94E+06	5.80E+06	2.25E+05	6.45E+05

Cage 2

	Total Flux [p/s]					
control	1.21E+06	2.55E+06	2.45E+05	5.10E+07	1.06E+08	3.06E+08
control	9.36E+05	2.88E+06	4.40E+06	1.14E+07	3.43E+07	1.93E+07
anti-miR-10b	5.65E+05	2.91E+06	8.66E+06	1.68E+07	1.98E+07	2.01E+08
anti-miR-10b	1.40E+06	2.37E+06	4.60E+06	1.42E+07	3.08E+07	4.58E+07

Group/ Days	minus 2/ mi	8 / minus 8	8 / minus 2	13 / minus 2
control	4.42	21.48	4.86	10.06
anti-miR-10b	3.32	8.23	2.48	3.47
control	5.51	26.72	4.85	15.73
anti-miR-10b	5.44	0.49	0.09	0.26
control	2.11	87.77	41.61	119.98
control	3.07	36.61	11.92	6.72
anti-miR-10b	5.15	35.07	6.81	69.05
anti-miR-10b	1.69	22.02	13.03	19.37
control	3.78	**43.15**	**15.81**	**38.12**
anti-miR-10b	3.90	**16.45**	**5.60**	**23.04**
control SD	1.49	30.40	17.52	54.70
anti-miR-10b S	1.75	15.27	5.68	31.79
control SE	0.75	15.20	8.76	27.35
anti-miR-10b S	0.87	7.64	2.84	15.90

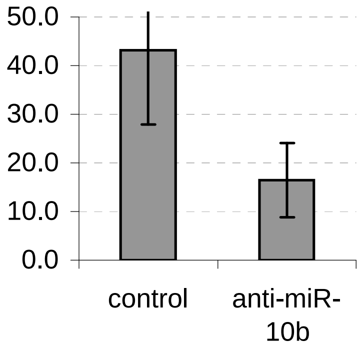

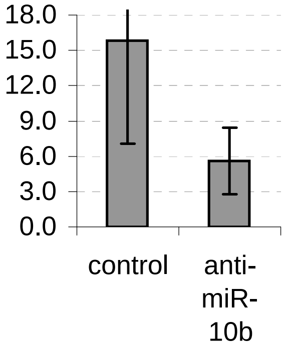

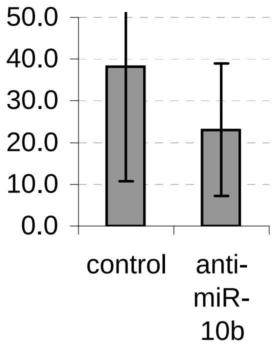

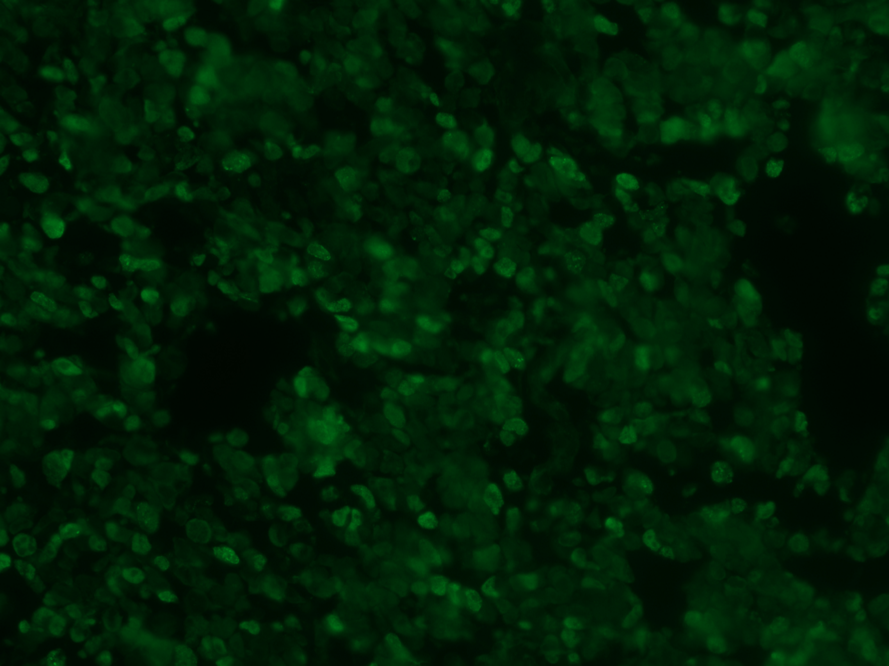

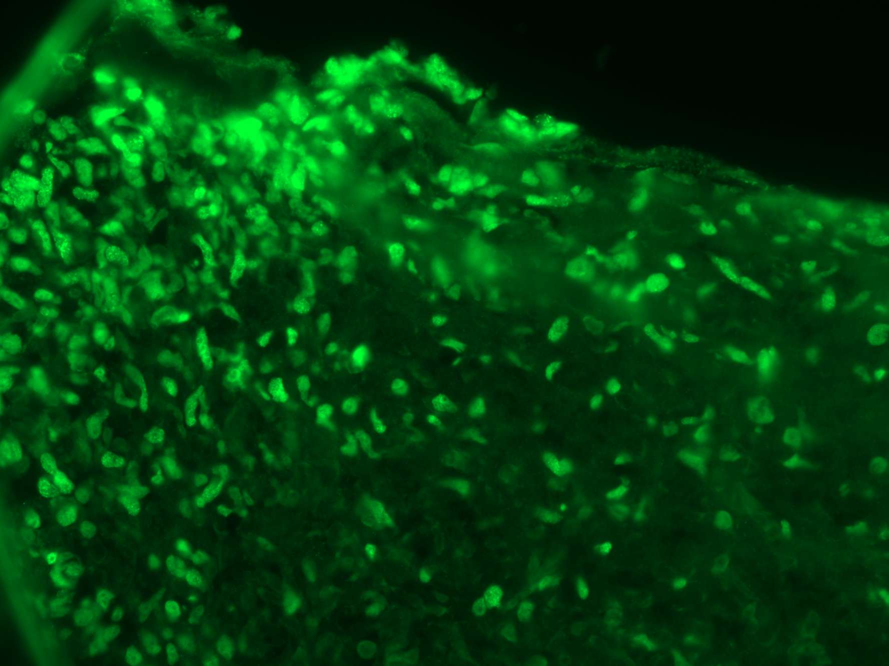

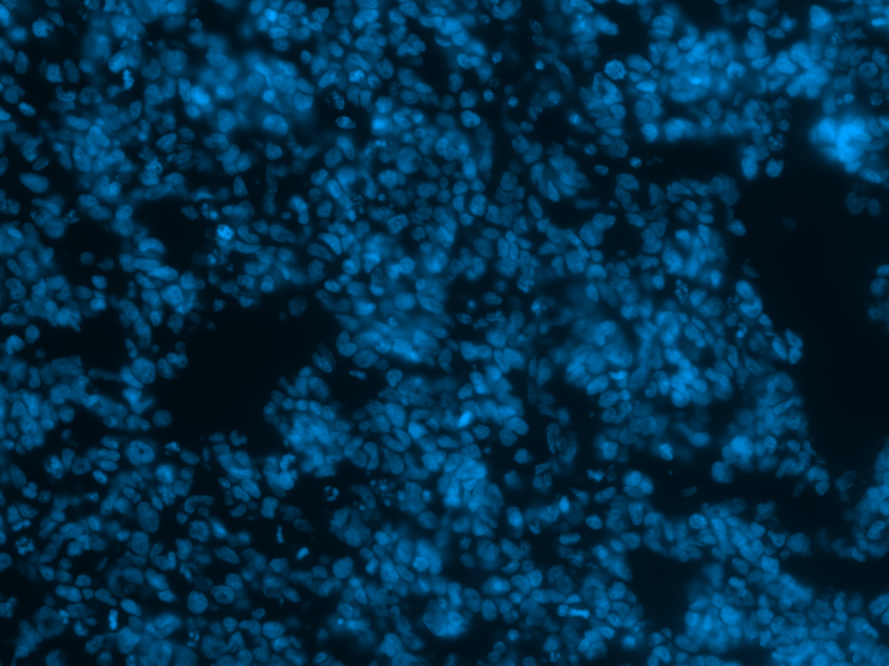

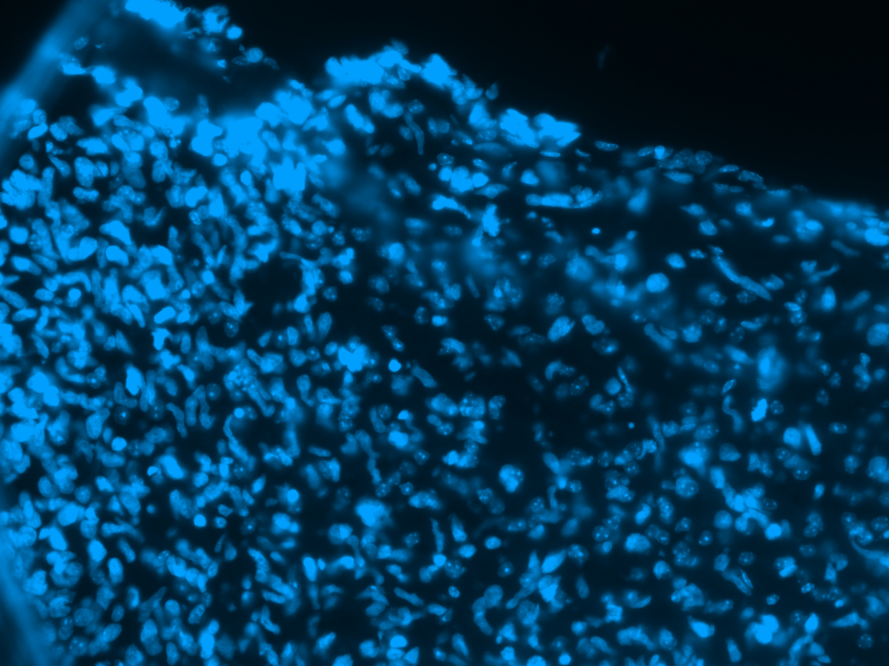

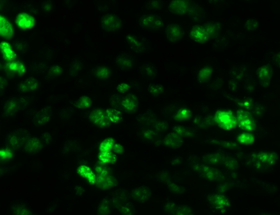

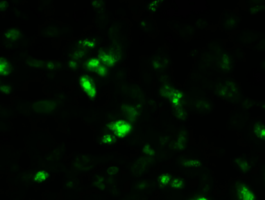

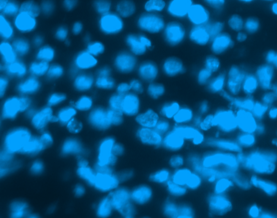

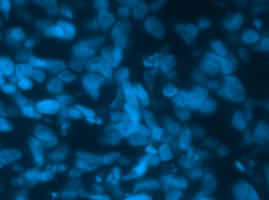

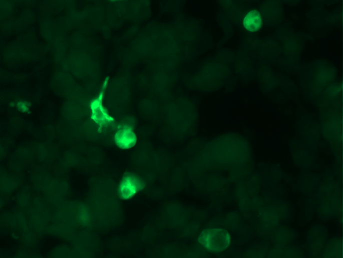

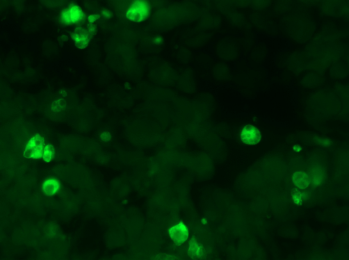

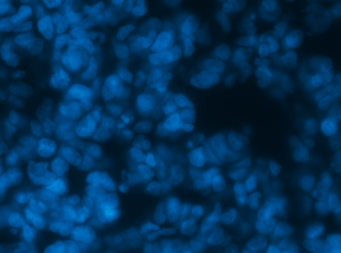

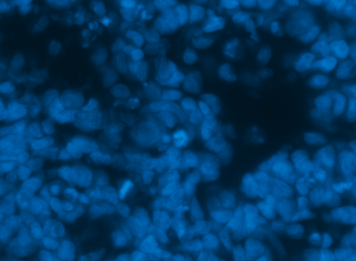

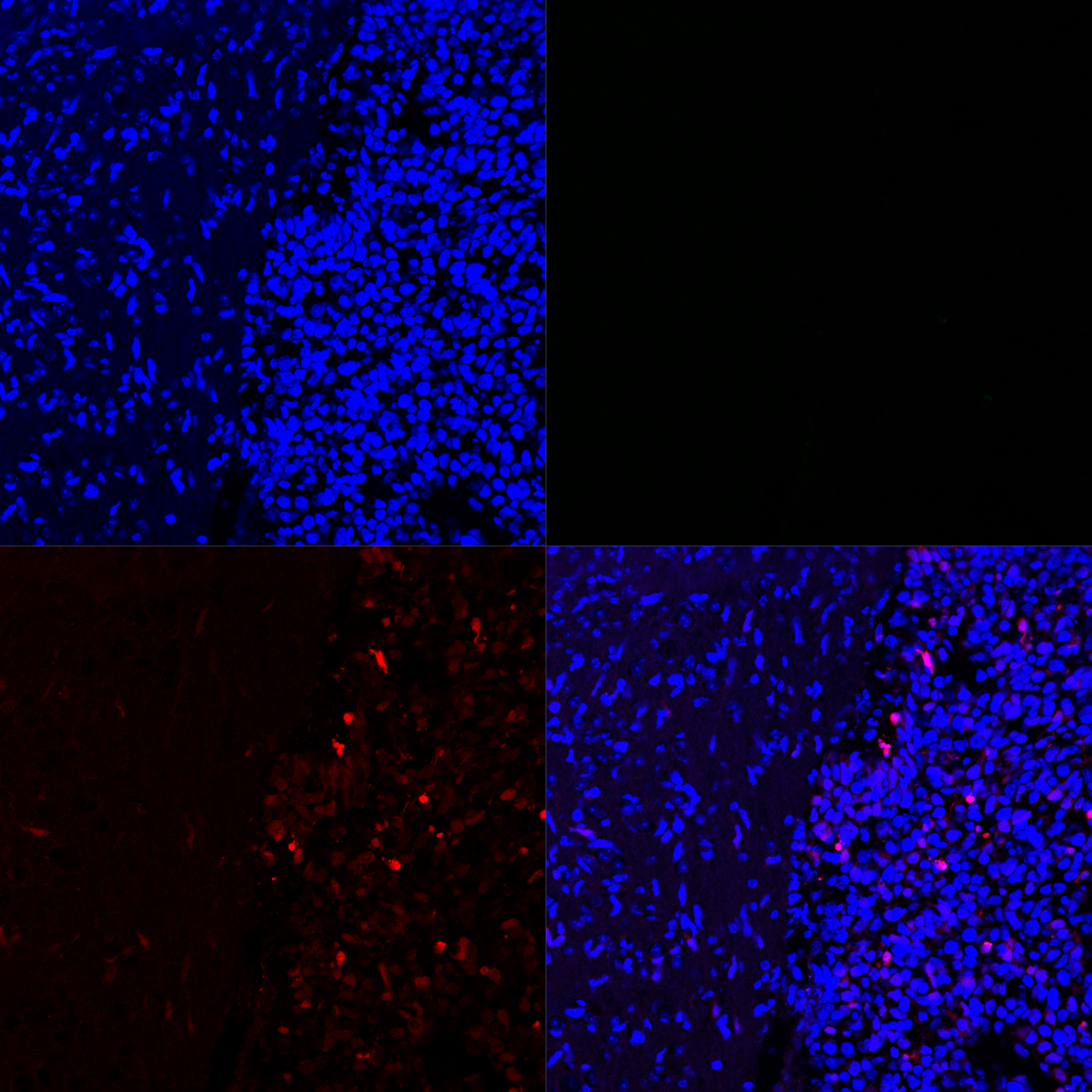

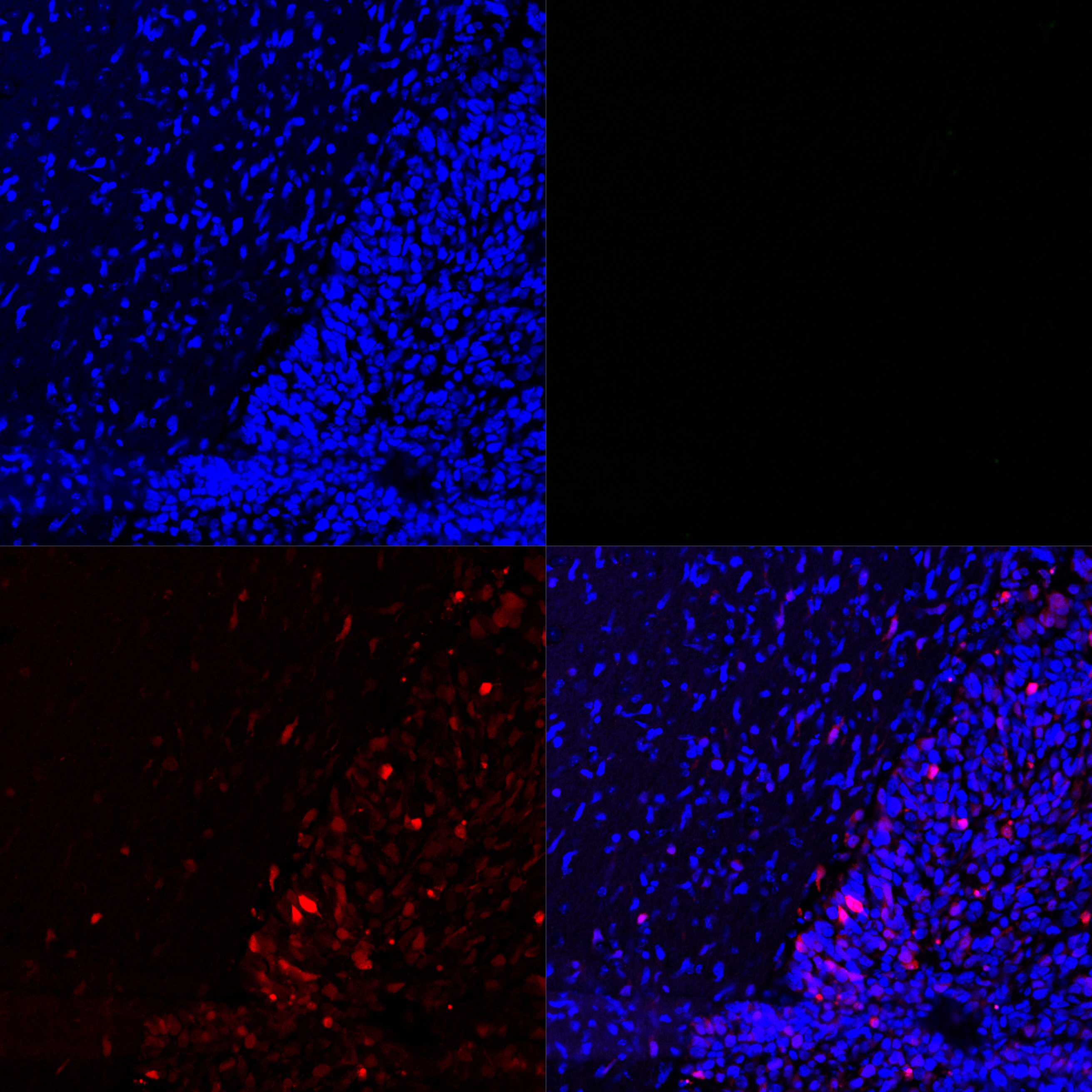

Figure 8 Panel F Source Data

**Tumor 1**	Label	Area	%Area		Label	Area	%Area	
1	BatchResu	1443520	50.085	1	Snap-7496	2567337	1.084	2.164321
2	BatchResu	1443520	34.037	2	Snap-7498	2567337	12.805	37.62082
3	BatchResu	1443520	34.41	3	Snap-7500	2567337	10.474	30.43883
4	BatchResu	1443520	39.095	4	Snap-7502	2567337	14.232	36.40363
5	BatchResu	1443520	36.365	5	Snap-7504	2567337	9.737	26.77575
6	BatchResu	1443520	77.255	6	Snap-7506	2567337	16.911	21.88985
7	BatchResu	1443520	35.495	7	Snap-7508	2567337	0.019	0.053529
8	BatchResu	1443520	48.471	8	Snap-7510	2567337	0.007	0.014442
9	BatchResu	1443520	42.907	9	Snap-7512	2567337	7.382	17.20465
10	BatchResu	1443520	50.335	10	Snap-7514	2567337	5.797	11.51684
11	BatchResu	1443520	28.16	11	Snap-7516	2567337	5.313	18.86719
12	BatchResu	1443520	47.561	12	Snap-7518	2567337	4.413	9.278611
13	BatchResu	1443520	65.459	13	Snap-7520	2567337	0.514	0.785224
14	BatchResu	1443520	54.155	14	Snap-7522	2567337	0.975	1.800388
15	BatchResu	1443520	35.48	15	Snap-7524	2567337	3.69	10.40023
16	BatchResu	1443520	34.071	16	Snap-7526	2567337	18.467	54.20152
17	BatchResu	1443520	57.719	17	Snap-7528	2567337	0.005	0.008663
18	BatchResu	1443520	36.059	18	Snap-7530	2567337	7.487	20.76319
19	BatchResu	1443520	30.152	19	Snap-7532	2567337	2.439	8.089016
21	BatchResu	1443520	43.56	21	Snap-7536	2567337	0.301	0.691001
22	BatchResu	1443520	32.71	22	Snap-7538	2567337	2.178	6.658514
23	BatchResu	1443520	29.084	23	Snap-7540	2567337	0.075	0.257874
24	BatchResu	1443520	28.225	24	Snap-7542	2567337	1.703	6.033658
25	BatchResu	1443520	30.054	25	Snap-7544	2567337	0.381	1.267718
26	BatchResu	1443520	31.139	26	Snap-7546	2567337	0.339	1.088667
27	BatchResu	1443520	20.556	27	Snap-7548	2567337	0.037	0.179996
28	BatchResu	1443520	36.288	28	Snap-7550	2567337	0.068	0.18739
29	BatchResu	1443520	32.639	29	Snap-7552	2567337	0.228	0.698551
30	BatchResu	1443520	43.45	30	Snap-7554	2567337	3.667	8.439586
31	BatchResu	1443520	64.859	31	Snap-7557	2567337	2.6	4.008696
32	BatchResu	1443520	51.875	32	Snap-7559	2567337	0.002	0.003855
34	BatchResu	1443520	34.097	34	Snap-7563	2567337	0.055	0.161305
35	BatchResu	1443520	37.8	35	Snap-7565	2567337	0.036	0.095238
36	BatchResu	1443520	40.483	36	Snap-7567	2567337	9.634	23.79764
37	BatchResu	1443520	40.657	37	Snap-7569	2567337	0.02	0.049192
39	BatchResu	1443520	34.18	39	Snap-7573	2567337	0.003	0.008777
40	BatchResu	1443520	31.859	40	Snap-7575	2567337	12.214	38.33768
41	BatchResu	1443520	62.454	41	Snap-7577	2567337	4.303	6.889871
42	BatchResu	1443520	68.279	42	Snap-7579	2567337	2.597	3.803512
43	BatchResu	1443520	61.183	43	Snap-7581	2567337	2.527	4.130232
44	BatchResu	1443520	68.283	44	Snap-7583	2567337	21.41	31.3548
45	BatchResu	1443520	75.111	45	Snap-7585	2567337	0.014	0.018639
46	BatchResu	1443520	51.636	46	Snap-7587	2567337	5.849	11.32737
47	BatchResu	1443520	61.622	47	Snap-7589	2567337	2.206	3.57989
50	BatchResu	1443520	61.983	50	Snap-7595	2567337	13.278	21.422
51	BatchResu	1443520	85.244	51	Snap-7597	2567337	0.002	0.002346
52	BatchResu	1443520	55.044	52	Snap-7599	2567337	5.274	9.581426
53	BatchResu	1443520	49.793	53	Snap-7601	2567337	21.189	42.55417

54	BatchResu	1443520	48.853	54	Snap-7603	2567337	16.032	32.81682

Figure 8 Panel F Source Data

55	BatchResu	1443520	80.087	55	Snap-7605	2567337	11.535	14.40309
56	BatchResu	1443520	62.404	56	Snap-7607	2567337	1.879	3.011025
57	BatchResu	1443520	55.191	57	Snap-7609	2567337	0.101	0.183001
58	BatchResu	1443520	74.184	58	Snap-7611	2567337	0.159	0.214332
59	BatchResu	1443520	77.568	59	Snap-7613	2567337	0.359	0.46282
60	BatchResu	1443520	68.382	60	Snap-7615	2567337	0.065	0.095054
61	BatchResu	1443520	73.131	61	Snap-7617	2567337	0.411	0.562005
62	BatchResu	1443520	80.763	62	Snap-7619	2567337	2.575	3.188341
63	BatchResu	1443520	54.069	63	Snap-7621	2567337	0.005	0.009247
64	BatchResu	1443520	37.464	64	Snap-7623	2567337	12.237	32.66336
65	BatchResu	1443520	48.263	65	Snap-7625	2567337	0.673	1.394443
66	BatchResu	1443520	75.93	66	Snap-7627	2567337	0.002	0.002634
67	BatchResu	1443520	42.968	67	Snap-7629	2567337	2.102	4.892013
68	BatchResu	1443520	56.916	68	Snap-7631	2567337	0.297	0.521822
69	BatchResu	1443520	46.315	69	Snap-7633	2567337	0.455	0.982403
70	BatchResu	1443520	49.765	70	Snap-7635	2567337	0.737	1.480961
71	BatchResu	1443520	60.112	71	Snap-7637	2567337	3.505	5.830783
72	BatchResu	1443520	77.561	72	Snap-7639	2567337	0.918	1.183585
73	BatchResu	1443520	71.57	73	Snap-7641	2567337	4.83	6.748638
74	BatchResu	1443520	47.198	74	Snap-7643	2567337	4.38	9.280054
								**9.490329**
								12.95396
								**1.505867**

Figure 8 Panel F Source Data

**Tumor 2**	Label	Area	%Area		Label	Area	%Area	
1	Snap-7704	2567337	37.777	1	Snap-7705	2567337	0.312	0.825899
2	Snap-7707	2567337	51.591	2	Snap-7708	2567337	1.646	3.190479
3	Snap-7709	2567337	56.572	3	Snap-7710	2567337	1.702	3.008555
4	Snap-7711	2567337	59.542	4	Snap-7712	2567337	4.273	7.176447
5	Snap-7713	2567337	61.257	5	Snap-7714	2567337	8.673	14.15838
6	Snap-7715	2567337	73.425	6	Snap-7716	2567337	3.565	4.855295
7	Snap-7717	2567337	58.698	7	Snap-7718	2567337	1.496	2.548639
8	Snap-7719	2567337	62.66	8	Snap-7720	2567337	1.367	2.181615
9	Snap-7721	2567337	54.541	9	Snap-7722	2567337	0.723	1.325608
10	Snap-7723	2567337	59.601	10	Snap-7724	2567337	0.809	1.35736
11	Snap-7725	2567337	62.349	11	Snap-7726	2567337	4.13	6.624004
12	Snap-7727	2567337	62.385	12	Snap-7728	2567337	1.654	2.651278
13	Snap-7729	2567337	54.549	13	Snap-7730	2567337	0.407	0.746118
14	Snap-7731	2567337	53.398	14	Snap-7732	2567337	0.221	0.413873
15	Snap-7733	2567337	49.134	15	Snap-7734	2567337	0.199	0.405015
16	Snap-7735	2567337	46.79	16	Snap-7736	2567337	0.207	0.442402
17	Snap-7737	2567337	45.396	17	Snap-7738	2567337	0.534	1.176315
18	Snap-7739	2567337	59.194	18	Snap-7740	2567337	0.009	0.015204
19	Snap-7741	2567337	57.941	19	Snap-7742	2567337	0.137	0.236447
20	Snap-7743	2567337	51.664	20	Snap-7744	2567337	1.118	2.163983
21	Snap-7745	2567337	59.175	21	Snap-7746	2567337	0.808	1.365441
22	Snap-7747	2567337	47.358	22	Snap-7748	2567337	0.078	0.164703
23	Snap-7749	2567337	53.947	23	Snap-7750	2567337	0.232	0.430052
24	Snap-7751	2567337	52.1	24	Snap-7752	2567337	0.043	0.082534
25	Snap-7753	2567337	53.234	25	Snap-7754	2567337	0.092	0.172822
26	Snap-7755	2567337	41.175	26	Snap-7756	2567337	0.06	0.145719
27	Snap-7757	2567337	22.705	27	Snap-7758	2567337	0.119	0.524114
28	Snap-7759	2567337	24.356	28	Snap-7760	2567337	1.688	6.93053
29	Snap-7761	2567337	31.805	29	Snap-7762	2567337	2.788	8.765917
30	Snap-7763	2567337	32.221	30	Snap-7764	2567337	3.904	12.11632
31	Snap-7765	2567337	60.6	31	Snap-7766	2567337	0.174	0.287129
32	Snap-7767	2567337	45.209	32	Snap-7768	2567337	0.045	0.099538
33	Snap-7769	2567337	53.129	33	Snap-7770	2567337	2.238	4.212389
34	Snap-7771	2567337	59.294	34	Snap-7772	2567337	3.522	5.939893
35	Snap-7773	2567337	60.12	35	Snap-7774	2567337	1.656	2.754491
36	Snap-7775	2567337	62.7	36	Snap-7776	2567337	0.158	0.251994
37	Snap-7777	2567337	44.432	37	Snap-7778	2567337	0.18	0.405113
38	Snap-7779	2567337	39.237	38	Snap-7780	2567337	0.967	2.464511
39	Snap-7781	2567337	36.714	39	Snap-7782	2567337	0.264	0.719072
40	Snap-7783	2567337	66.183	40	Snap-7784	2567337	0.395	0.59683
41	Snap-7785	2567337	66.41	41	Snap-7786	2567337	0.255	0.383978
42	Snap-7787	2567337	55.998	42	Snap-7788	2567337	1.657	2.959034
43	Snap-7789	2567337	50.711	43	Snap-7790	2567337	0.942	1.857585
44	Snap-7791	2567337	58.517	44	Snap-7792	2567337	0.011	0.018798
45	Snap-7793	2567337	49.584	45	Snap-7794	2567337	0.237	0.477977
46	Snap-7795	2567337	54.389	46	Snap-7796	2567337	0.054	0.099285
47	Snap-7797	2567337	42.384	47	Snap-7798	2567337	0.02	0.047188
48	Snap-7799	2567337	53.887	48	Snap-7800	2567337	4.226	7.842337

49	Snap-7801	2567337	50.795		49	Snap-7802	2567337	2.931	5.770253

Figure 8 Panel F Source Data

50	Snap-7803	2567337	39.633		50	Snap-7804	2567337	0.005	0.012616
51	Snap-7805	2567337	52.683		51	Snap-7806	2567337	0.066	0.125278
52	Snap-7807	2567337	32.234		52	Snap-7808	2567337	0.101	0.313334
53	Snap-7809	2567337	39.869		53	Snap-7810	2567337	0.091	0.228248
54	Snap-7811	2567337	33.884		54	Snap-7812	2567337	3.504	10.34116
55	Snap-7813	2567337	42.342		55	Snap-7814	2567337	1.398	3.301686
56	Snap-7815	2567337	23.326		56	Snap-7816	2567337	7.346	31.49275
57	Snap-7817	2567337	35.014		57	Snap-7818	2567337	0.27	0.77112
58	Snap-7819	2567337	51.706		58	Snap-7820	2567337	0.562	1.086914
59	Snap-7821	2567337	44.917		59	Snap-7822	2567337	1.602	3.566578
60	Snap-7823	2567337	53.643		60	Snap-7824	2567337	8.232	15.3459
61	Snap-7825	2567337	39.722		61	Snap-7826	2567337	1.653	4.161422
62	Snap-7827	2567337	55.058		62	Snap-7828	2567337	1.034	1.87802
63	Snap-7829	2567337	36.986		63	Snap-7830	2567337	0.205	0.554264
64	Snap-7831	2567337	38.342		64	Snap-7832	2567337	0.208	0.542486
									**3.079848**
									5.026835
									**0.628354**

Figure 8 Panel F Source Data

Tumor 3	Label	Area	%Area		Label	Area	%Area	
1	Snap-7891	2567337	82.241	1	Snap-7892	2567337	0.604	0.734427
2	Snap-7893	2567337	70.089	2	Snap-7894	2567337	4.026	5.744125
3	Snap-7895	2567337	71.496	3	Snap-7896	2567337	4.656	6.512252
4	Snap-7897	2567337	67.01	4	Snap-7898	2567337	2.15	3.208476
5	Snap-7899	2567337	50.3	5	Snap-7900	2567337	2.467	4.904573
6	Snap-7901	2567337	51.956	6	Snap-7902	2567337	1.688	3.248903
7	Snap-7903	2567337	65.222	7	Snap-7904	2567337	0.144	0.220784
8	Snap-7905	2567337	68.955	8	Snap-7906	2567337	0.177	0.256689
9	Snap-7907	2567337	55.031	9	Snap-7908	2567337	0.017	0.030892
10	Snap-7909	2567337	50.691	10	Snap-7910	2567337	0.018	0.035509
11	Snap-7911	2567337	42.047	11	Snap-7912	2567337	0.008	0.019026
12	Snap-7913	2567337	49.248	12	Snap-7914	2567337	1.046	2.123944
13	Snap-7916	2567337	70.435	13	Snap-7917	2567337	4.756	6.752325
14	Snap-7918	2567337	67.251	14	Snap-7919	2567337	6.161	9.161202
15	Snap-7920	2567337	63.443	15	Snap-7921	2567337	6.294	9.920716
16	Snap-7922	2567337	64.317	16	Snap-7923	2567337	1.373	2.134739
17	Snap-7924	2567337	67.809	17	Snap-7925	2567337	0.22	0.324441
18	Snap-7926	2567337	57.471	18	Snap-7927	2567337	0.4	0.696003
19	Snap-7928	2567337	59.767	19	Snap-7929	2567337	0.821	1.373668
20	Snap-7930	2567337	35.639	20	Snap-7931	2567337	0.1	0.280591
21	Snap-7932	2567337	35.906	21	Snap-7933	2567337	2.972	8.277168
22	Snap-7934	2567337	49.72	22	Snap-7935	2567337	0.74	1.488335
23	Snap-7936	2567337	43.216	23	Snap-7937	2567337	0.77	1.781748
24	Snap-7938	2567337	21.532	24	Snap-7939	2567337	1.636	7.597994
25	Snap-7940	2567337	32.635	25	Snap-7941	2567337	2.975	9.11598
26	Snap-7942	2567337	43.454	26	Snap-7943	2567337	0.107	0.246237
27	Snap-7944	2567337	60.665	27	Snap-7945	2567337	0.077	0.126927
28	Snap-7946	2567337	46.141	28	Snap-7947	2567337	0.279	0.604668
29	Snap-7948	2567337	48.561	29	Snap-7949	2567337	3.35	6.89854
30	Snap-7950	2567337	46.361	30	Snap-7951	2567337	3.776	8.144777
31	Snap-7952	2567337	53.116	31	Snap-7953	2567337	0.054	0.101664
32	Snap-7954	2567337	58.979	32	Snap-7955	2567337	5.68	9.630546
33	Snap-7956	2567337	51.551	33	Snap-7957	2567337	0.142	0.275455
34	Snap-7958	2567337	47.522	34	Snap-7959	2567337	2.084	4.385337
35	Snap-7960	2567337	45.234	35	Snap-7961	2567337	3.024	6.685237
36	Snap-7962	2567337	56.091	36	Snap-7963	2567337	6.659	11.87178
37	Snap-7964	2567337	54.534	37	Snap-7965	2567337	2.147	3.936993
38	Snap-7966	2567337	57.374	38	Snap-7967	2567337	0.522	0.90982
39	Snap-7968	2567337	50.79	39	Snap-7969	2567337	10.481	20.63595
40	Snap-7978	2567337	32.78	40	Snap-7979	2567337	1.157	3.529591
41	Snap-7980	2567337	43.976	41	Snap-7981	2567337	0.022	0.050027
42	Snap-7982	2567337	71.986	42	Snap-7983	2567337	1.282	1.780902
43	Snap-7984	2567337	66.239	43	Snap-7985	2567337	6.346	9.580459
44	Snap-7986	2567337	40.415	44	Snap-7987	2567337	0.024	0.059384
45	Snap-8008	2567337	53.815	45	Snap-8009	2567337	6.048	11.2385
								**4.147496**
								4.495317
								**0.670122**

Figure 8 Panel F Source Data

**Tumor 4**	Label	Area	%Area		Label	Area	%Area	
1	Snap-8605	2567337	38.548	1	Snap-8606	2567337	0.015	0.038913
2	Snap-8607	2567337	52.264	2	Snap-8608	2567337	0.877	1.678019
3	Snap-8609	2567337	64.553	3	Snap-8610	2567337	1.038	1.607981
4	Snap-8611	2567337	63.808	4	Snap-8612	2567337	0.38	0.595537
5	Snap-8613	2567337	54.822	5	Snap-8614	2567337	0.041	0.074787
6	Snap-8615	2567337	45.616	6	Snap-8616	2567337	0.028	0.061382
7	Snap-8617	2567337	24.232	7	Snap-8618	2567337	0.023	0.094916
8	Snap-8619	2567337	53.905	8	Snap-8620	2567337	0.004	0.00742
9	Snap-8621	2567337	67.834	9	Snap-8622	2567337	0.86	1.267801
10	Snap-8623	2567337	69.337	10	Snap-8624	2567337	0.316	0.455745
11	Snap-8625	2567337	56.133	11	Snap-8626	2567337	0.201	0.358078
12	Snap-8627	2567337	58.653	12	Snap-8628	2567337	0.746	1.271887
13	Snap-8629	2567337	69.253	13	Snap-8630	2567337	0.17	0.245477
14	Snap-8631	2567337	46.305	14	Snap-8632	2567337	0.043	0.092863
15	Snap-8633	2567337	37.052	15	Snap-8634	2567337	0.013	0.035086
16	Snap-8635	2567337	41.314	16	Snap-8636	2567337	0.125	0.302561
17	Snap-8637	2567337	24.005	17	Snap-8638	2567337	0.507	2.11206
18	Snap-8639	2567337	43.237	18	Snap-8640	2567337	5.179	11.97817
19	Snap-8641	2567337	52.455	19	Snap-8642	2567337	2.056	3.91955
20	Snap-8643	2567337	54.132	20	Snap-8644	2567337	2.51	4.636814
21	Snap-8645	2567337	44.606	21	Snap-8646	2567337	0.073	0.163655
22	Snap-8647	2567337	70.381	22	Snap-8648	2567337	0.025	0.035521
23	Snap-8649	2567337	54.227	23	Snap-8650	2567337	0.161	0.2969
24	Snap-8651	2567337	52.698	24	Snap-8652	2567337	0.046	0.08729
25	Snap-8653	2567337	58.47	25	Snap-8654	2567337	0.03	0.051308
26	Snap-8655	2567337	63.272	26	Snap-8656	2567337	1.949	3.080351
27	Snap-8657	2567337	66.925	27	Snap-8658	2567337	0.187	0.279417
28	Snap-8659	2567337	53.15	28	Snap-8660	2567337	1.663	3.128881
29	Snap-8661	2567337	49.657	29	Snap-8662	2567337	0.287	0.577965
30	Snap-8663	2567337	33.315	30	Snap-8664	2567337	1.615	4.847666
31	Snap-8665	2567337	32.438	31	Snap-8666	2567337	2.036	6.276589
32	Snap-8667	2567337	45.537	32	Snap-8668	2567337	0.849	1.864418
33	Snap-8669	2567337	56.589	33	Snap-8670	2567337	0.013	0.022973
34	Snap-8671	2567337	64.578	34	Snap-8672	2567337	0.834	1.291461
35	Snap-8673	2567337	62.875	35	Snap-8674	2567337	0.261	0.415109
36	Snap-8675	2567337	53.068	36	Snap-8676	2567337	0.033	0.062184
37	Snap-8677	2567337	66.52	37	Snap-8678	2567337	0.363	0.545701
38	Snap-8679	2567337	82.284	38	Snap-8680	2567337	0.098	0.1191
39	Snap-8681	2567337	62.594	39	Snap-8682	2567337	0.699	1.11672
40	Snap-8683	2567337	42.495	40	Snap-8684	2567337	0.042	0.098835
41	Snap-8685	2567337	31.863	41	Snap-8686	2567337	0.241	0.756363
42	Snap-8687	2567337	31.75	42	Snap-8688	2567337	1.483	4.670866
43	Snap-8689	2567337	50.945	43	Snap-8690	2567337	1.255	2.463441
44	Snap-8691	2567337	57.401	44	Snap-8692	2567337	0.315	0.548771
45	Snap-8693	2567337	57.956	45	Snap-8694	2567337	0.521	0.898958
46	Snap-8695	2567337	26.667	46	Snap-8696	2567337	0.996	3.734953
47	Snap-8697	2567337	37.883	47	Snap-8698	2567337	1.429	3.772141
48	Snap-8699	2567337	51.432	48	Snap-8700	2567337	1.187	2.307902

49	Snap-8701	2567337	45.118	49	Snap-8702	2567337	1.641	3.637129

Figure 8 Panel F Source Data

50	Snap-8703	2567337	28.166	50	Snap-8704	2567337	3.595	12.76362
51	Snap-8705	2567337	14.141	51	Snap-8706	2567337	2.732	19.31971
52	Snap-8707	2567337	30.316	52	Snap-8708	2567337	2.981	9.833091
53	Snap-8709	2567337	25.972	53	Snap-8710	2567337	2.11	8.124134
54	Snap-8711	2567337	21.574	54	Snap-8712	2567337	2.099	9.729304
55	Snap-8713	2567337	19.998	55	Snap-8714	2567337	3.064	15.32153
56	Snap-8715	2567337	27.24	56	Snap-8716	2567337	3.953	14.51175
57	Snap-8717	2567337	44.273	57	Snap-8718	2567337	2.027	4.578411
58	Snap-8719	2567337	56.383	58	Snap-8720	2567337	1.03	1.826792
59	Snap-8721	2567337	53.873	59	Snap-8722	2567337	1.084	2.01214
60	Snap-8723	2567337	71.57	60	Snap-8724	2567337	1.132	1.581668
61	Snap-8725	2567337	56.424	61	Snap-8726	2567337	0.037	0.065575
62	Snap-8727	2567337	58.133	62	Snap-8728	2567337	0.035	0.060207
63	Snap-8729	2567337	53.924	63	Snap-8730	2567337	1.112	2.062162
64	Snap-8731	2567337	34.953	64	Snap-8732	2567337	0.017	0.048637
65	Snap-8733	2567337	62.607	65	Snap-8734	2567337	0.438	0.699602
66	Snap-8735	2567337	62.038	66	Snap-8736	2567337	0.349	0.562558
67	Snap-8737	2567337	60.237	67	Snap-8738	2567337	0.175	0.290519
68	Snap-8739	2567337	66.121	68	Snap-8740	2567337	0.234	0.353897
69	Snap-8741	2567337	79.593	69	Snap-8742	2567337	0.832	1.045318
70	Snap-8743	2567337	62.713	70	Snap-8744	2567337	0.917	1.462217
71	Snap-8745	2567337	60.351	71	Snap-8746	2567337	0.114	0.188895
72	Snap-8747	2567337	58.219	72	Snap-8748	2567337	0.173	0.297154
73	Snap-8749	2567337	60.29	73	Snap-8750	2567337	0.04	0.066346
74	Snap-8753	2567337	61.5	74	Snap-8754	2567337	0.188	0.305691
75	Snap-8755	2567337	63.461	75	Snap-8756	2567337	1.513	2.384141
76	Snap-8757	2567337	57.368	76	Snap-8758	2567337	2.808	4.894715
77	Snap-8759	2567337	64.902	77	Snap-8760	2567337	5.55	8.551354
78	Snap-8761	2567337	69.136	78	Snap-8762	2567337	1.803	2.607903
79	Snap-8763	2567337	52.512	79	Snap-8764	2567337	0.143	0.272319
80	Snap-8765	2567337	58.635	80	Snap-8766	2567337	0.221	0.376908
81	Snap-8767	2567337	58.433	81	Snap-8768	2567337	0.045	0.077011
82	Snap-8769	2567337	44.991	82	Snap-8770	2567337	0.924	2.053744
83	Snap-8771	2567337	45.538	83	Snap-8772	2567337	0.074	0.162502
84	Snap-8773	2567337	54.818	84	Snap-8774	2567337	0.097	0.176949
85	Snap-8775	2567337	61.239	85	Snap-8776	2567337	0.154	0.251474
86	Snap-8777	2567337	51.283	86	Snap-8778	2567337	0.5	0.974982
87	Snap-8779	2567337	49.398	87	Snap-8780	2567337	1.911	3.868578
88	Snap-8781	2567337	50.606	88	Snap-8782	2567337	0.621	1.227127
89	Snap-8783	2567337	54.198	89	Snap-8784	2567337	0.21	0.387468
90	Snap-8785	2567337	27.088	90	Snap-8786	2567337	0.509	1.879061
								**2.391609**
								3.761254
								**0.396471**

Figure 8 Panel F Source Data

**Tumor 5**	Label	Area	%Area		Label	Area	%Area	
1	Snap-8485	2567337	53.347	1	Snap-8486	2567337	7.417	13.90331
2	Snap-8487	2567337	64.779	2	Snap-8488	2567337	18.746	28.93839
3	Snap-8489	2567337	55.485	3	Snap-8490	2567337	22.513	40.57493
4	Snap-8491	2567337	22.983	4	Snap-8492	2567337	8.004	34.82574
5	Snap-8493	2567337	61.301	5	Snap-8494	2567337	9.439	15.39779
6	Snap-8495	2567337	51.96	6	Snap-8496	2567337	5.987	11.52232
7	Snap-8497	2567337	44.653	7	Snap-8498	2567337	3.076	6.888675
8	Snap-8499	2567337	41.262	8	Snap-8500	2567337	0.945	2.290243
9	Snap-8501	2567337	50.773	9	Snap-8502	2567337	3.817	7.517775
10	Snap-8503	2567337	42.503	10	Snap-8504	2567337	13.366	31.44719
11	Snap-8505	2567337	43.001	11	Snap-8506	2567337	12.108	28.15748
12	Snap-8507	2567337	48.588	12	Snap-8508	2567337	0.946	1.946983
13	Snap-8509	2567337	48.905	13	Snap-8510	2567337	1.662	3.398426
14	Snap-8511	2567337	45.989	14	Snap-8512	2567337	0.421	0.915436
15	Snap-8513	2567337	43.701	15	Snap-8514	2567337	0.653	1.494245
16	Snap-8515	2567337	40.98	16	Snap-8516	2567337	2.336	5.700342
17	Snap-8517	2567337	52.876	17	Snap-8518	2567337	0.083	0.156971
18	Snap-8519	2567337	47.494	18	Snap-8520	2567337	0.772	1.625468
19	Snap-8521	2567337	30.941	19	Snap-8522	2567337	0.569	1.838984
20	Snap-8523	2567337	48.827	20	Snap-8524	2567337	0.365	0.747537
21	Snap-8525	2567337	51.744	21	Snap-8526	2567337	0.904	1.747062
22	Snap-8527	2567337	57.732	22	Snap-8528	2567337	0.24	0.415714
23	Snap-8529	2567337	67.583	23	Snap-8530	2567337	0.9	1.331696
24	Snap-8531	2567337	71.54	24	Snap-8532	2567337	0.588	0.821918
25	Snap-8533	2567337	72.164	25	Snap-8534	2567337	2.399	3.324372
26	Snap-8535	2567337	65.658	26	Snap-8536	2567337	1.408	2.144445
27	Snap-8537	2567337	66.924	27	Snap-8538	2567337	0.227	0.339191
28	Snap-8539	2567337	71.527	28	Snap-8540	2567337	3.409	4.766032
29	Snap-8541	2567337	70.937	29	Snap-8542	2567337	0.711	1.002298
30	Snap-8543	2567337	62.059	30	Snap-8544	2567337	0.713	1.148907
31	Snap-8545	2567337	60.853	31	Snap-8546	2567337	0.238	0.391106
32	Snap-8547	2567337	65.227	32	Snap-8548	2567337	9.378	14.37748
33	Snap-8549	2567337	48.238	33	Snap-8550	2567337	0.886	1.836726
34	Snap-8551	2567337	57.669	34	Snap-8552	2567337	1.933	3.351887
35	Snap-8553	2567337	71.433	35	Snap-8554	2567337	10.601	14.84048
36	Snap-8555	2567337	74.589	36	Snap-8556	2567337	7.997	10.72142
37	Snap-8557	2567337	68.515	37	Snap-8558	2567337	4.855	7.08604
38	Snap-8559	2567337	65.556	38	Snap-8560	2567337	0.191	0.291354
39	Snap-8561	2567337	59.129	39	Snap-8562	2567337	0.607	1.026569
40	Snap-8563	2567337	56.636	40	Snap-8564	2567337	0.789	1.393107
41	Snap-8565	2567337	47.484	41	Snap-8566	2567337	3.245	6.833881
42	Snap-8567	2567337	50.159	42	Snap-8568	2567337	3.098	6.176359
43	Snap-8569	2567337	40.584	43	Snap-8570	2567337	10.992	27.08457
44	Snap-8571	2567337	45.254	44	Snap-8572	2567337	1.525	3.369868
45	Snap-8573	2567337	44.107	45	Snap-8574	2567337	0.582	1.319518
46	Snap-8575	2567337	38.413	46	Snap-8576	2567337	0.353	0.91896
47	Snap-8577	2567337	51.577	47	Snap-8578	2567337	0.008	0.015511
48	Snap-8579	2567337	47.551	48	Snap-8580	2567337	0.632	1.329099

49	Snap-8581	2567337	49.939	49	Snap-8582	2567337	0.155	0.310379

Figure 8 Panel F Source Data

50	Snap-8583	2567337	45.519	50	Snap-8584	2567337	0.301	0.661262
51	Snap-8585	2567337	44.317	51	Snap-8586	2567337	0.076	0.171492
52	Snap-8587	2567337	46.261	52	Snap-8588	2567337	0.192	0.415036
53	Snap-8589	2567337	50.254	53	Snap-8590	2567337	1.481	2.947029
54	Snap-8591	2567337	59.173	54	Snap-8592	2567337	0.46	0.777382
55	Snap-8593	2567337	59.9	55	Snap-8594	2567337	0.785	1.310518
56	Snap-8595	2567337	56.007	56	Snap-8596	2567337	0.534	0.953452
57	Snap-8597	2567337	67.196	57	Snap-8598	2567337	10.254	15.25984
58	Snap-8599	2567337	56.708	58	Snap-8600	2567337	0.777	1.370177
59	Snap-8601	2567337	55.698	59	Snap-8602	2567337	0.701	1.258573
60	Snap-8603	2567337	45.556	60	Snap-8604	2567337	0.427	0.937308
								**6.417771**
								9.635206
								**1.2439**

Figure 8 Panel F Source Data

**Tumor 6**	Label	Area	%Area		Label	Area	%Area	
1	Snap-8341	2567337	36.12	1	Snap-8342	2567337	12.525	34.67608
2	Snap-8343	2567337	59.661	2	Snap-8344	2567337	8.711	14.60083
3	Snap-8345	2567337	52.868	3	Snap-8346	2567337	4.704	8.897632
4	Snap-8347	2567337	36.632	4	Snap-8348	2567337	2.541	6.936558
5	Snap-8349	2567337	53.12	5	Snap-8350	2567337	1.008	1.89759
6	Snap-8351	2567337	60.304	6	Snap-8352	2567337	0.624	1.034757
7	Snap-8353	2567337	50.415	7	Snap-8354	2567337	6.151	12.20073
8	Snap-8355	2567337	51.506	8	Snap-8356	2567337	0.677	1.31441
9	Snap-8357	2567337	57.59	9	Snap-8358	2567337	1.341	2.328529
10	Snap-8359	2567337	62.687	10	Snap-8360	2567337	4.539	7.240736
11	Snap-8361	2567337	62.74	11	Snap-8362	2567337	0.286	0.45585
12	Snap-8363	2567337	53.036	12	Snap-8364	2567337	0.303	0.57131
13	Snap-8365	2567337	55.65	13	Snap-8366	2567337	1.592	2.860737
14	Snap-8367	2567337	44.13	14	Snap-8368	2567337	7.792	17.65692
15	Snap-8369	2567337	53.463	15	Snap-8370	2567337	10.242	19.15717
16	Snap-8371	2567337	57.62	16	Snap-8372	2567337	1.228	2.131204
17	Snap-8373	2567337	63.878	17	Snap-8374	2567337	0.149	0.233257
18	Snap-8375	2567337	61.503	18	Snap-8376	2567337	1.276	2.074696
19	Snap-8377	2567337	82.052	19	Snap-8378	2567337	0.739	0.900648
20	Snap-8379	2567337	60.604	20	Snap-8380	2567337	0.195	0.321761
21	Snap-8381	2567337	49.049	21	Snap-8382	2567337	0.843	1.718689
22	Snap-8383	2567337	56.587	22	Snap-8384	2567337	0.657	1.161044
23	Snap-8385	2567337	53.48	23	Snap-8386	2567337	1.808	3.380703
24	Snap-8387	2567337	47.16	24	Snap-8388	2567337	4.941	10.4771
25	Snap-8389	2567337	45.851	25	Snap-8390	2567337	3.309	7.216855
26	Snap-8391	2567337	57.29	26	Snap-8392	2567337	0.904	1.577937
27	Snap-8393	2567337	59.918	27	Snap-8394	2567337	1.762	2.940686
28	Snap-8395	2567337	47.511	28	Snap-8396	2567337	0.175	0.368336
29	Snap-8397	2567337	54.648	29	Snap-8398	2567337	1.253	2.292856
30	Snap-8399	2567337	62.341	30	Snap-8400	2567337	0.048	0.076996
31	Snap-8401	2567337	54.171	31	Snap-8402	2567337	0.1	0.184601
32	Snap-8403	2567337	55.814	32	Snap-8404	2567337	0.806	1.444082
33	Snap-8405	2567337	61.48	33	Snap-8406	2567337	0.254	0.413142
34	Snap-8407	2567337	78.846	34	Snap-8408	2567337	0.429	0.544099
35	Snap-8409	2567337	60.908	35	Snap-8410	2567337	2.077	3.410061
36	Snap-8411	2567337	58.906	36	Snap-8412	2567337	0.374	0.63491
37	Snap-8413	2567337	76.617	37	Snap-8414	2567337	0.237	0.309331
38	Snap-8415	2567337	66.305	38	Snap-8416	2567337	0.146	0.220195
39	Snap-8417	2567337	79.325	39	Snap-8418	2567337	4.771	6.014497
40	Snap-8419	2567337	70.757	40	Snap-8420	2567337	13.392	18.92675
41	Snap-8423	2567337	50.298	41	Snap-8424	2567337	0.434	0.862857
42	Snap-8425	2567337	53.582	42	Snap-8426	2567337	6.348	11.84726
43	Snap-8449	2567337	49.034	43	Snap-8450	2567337	1.079	2.200514
44	Snap-8451	2567337	60.584	44	Snap-8452	2567337	0.048	0.079229
45	Snap-8453	2567337	60.64	45	Snap-8454	2567337	3.279	5.407322
46	Snap-8455	2567337	60.748	46	Snap-8456	2567337	0.034	0.055969
47	Snap-8457	2567337	59.602	47	Snap-8458	2567337	2.298	3.855575
48	Snap-8459	2567337	59.951	48	Snap-8460	2567337	0.83	1.384464

49	Snap-8461	2567337	58.61		49	Snap-8462	2567337	0.239	0.40778

Figure 8 Panel F Source Data

50	Snap-8463	2567337	62.266		50	Snap-8464	2567337	0.243	0.390261
51	Snap-8465	2567337	73.882		51	Snap-8466	2567337	2.669	3.612517
52	Snap-8467	2567337	76.907		52	Snap-8468	2567337	5.84E-04	0.00076
53	Snap-8469	2567337	59.289		53	Snap-8470	2567337	2.681	4.521918
54	Snap-8471	2567337	66.994		54	Snap-8472	2567337	2.281	3.404783
55	Snap-8473	2567337	50.327		55	Snap-8474	2567337	1.931	3.836907
56	Snap-8475	2567337	46.746		56	Snap-8476	2567337	1.812	3.876267
57	Snap-8477	2567337	56.347		57	Snap-8478	2567337	4.225	7.498181
58	Snap-8479	2567337	48.715		58	Snap-8480	2567337	5.383	11.04998
59	Snap-8481	2567337	52.364		59	Snap-8482	2567337	0.229	0.437323
60	Snap-8483	2567337	55.702		60	Snap-8484	2567337	0.638	1.145381
									**4.444659**
									6.268351
									**0.809241**

Figure 8 Panel F Source Data

Tumor 7	Label	Area	%Area		Label	Area	%Area	
1	Snap-8209	2567337	26.886	1	Snap-8210	2567337	7.235	26.90992
2	Snap-8211	2567337	65.637	2	Snap-8212	2567337	5.429	8.271249
3	Snap-8213	2567337	72.867	3	Snap-8214	2567337	0.548	0.752055
4	Snap-8215	2567337	62.703	4	Snap-8216	2567337	3.1	4.943942
5	Snap-8217	2567337	78.186	5	Snap-8218	2567337	3.215	4.111989
6	Snap-8219	2567337	77.704	6	Snap-8220	2567337	4.171	5.367806
7	Snap-8221	2567337	52.662	7	Snap-8222	2567337	8.262	15.68873
8	Snap-8223	2567337	56.35	8	Snap-8224	2567337	6.197	10.99734
9	Snap-8225	2567337	53.364	9	Snap-8226	2567337	0.924	1.731504
10	Snap-8227	2567337	54.633	10	Snap-8228	2567337	0.357	0.653451
11	Snap-8229	2567337	61.238	11	Snap-8230	2567337	0.298	0.486626
12	Snap-8231	2567337	62.905	12	Snap-8232	2567337	0.174	0.276608
13	Snap-8233	2567337	57.157	13	Snap-8234	2567337	1.414	2.473888
14	Snap-8235	2567337	58.831	14	Snap-8236	2567337	0.303	0.515035
15	Snap-8237	2567337	55.812	15	Snap-8238	2567337	0.01	0.017917
16	Snap-8239	2567337	55.954	16	Snap-8240	2567337	0.458	0.81853
17	Snap-8241	2567337	60.705	17	Snap-8242	2567337	0.002	0.003295
18	Snap-8243	2567337	53.05	18	Snap-8244	2567337	8.18E-04	0.001542
19	Snap-8245	2567337	56.464	19	Snap-8246	2567337	1.562	2.766364
20	Snap-8247	2567337	53.064	20	Snap-8248	2567337	0.045	0.084803
21	Snap-8249	2567337	64.297	21	Snap-8250	2567337	0.121	0.188189
22	Snap-8251	2567337	34.305	22	Snap-8252	2567337	0.35	1.020259
23	Snap-8253	2567337	59.107	23	Snap-8254	2567337	0.718	1.214746
24	Snap-8255	2567337	53.046	24	Snap-8256	2567337	0.721	1.359198
25	Snap-8257	2567337	54.865	25	Snap-8258	2567337	1.294	2.358516
26	Snap-8259	2567337	55.433	26	Snap-8260	2567337	0.638	1.150939
27	Snap-8261	2567337	65.388	27	Snap-8262	2567337	0.664	1.015477
28	Snap-8263	2567337	66.534	28	Snap-8264	2567337	0.793	1.191872
29	Snap-8265	2567337	60.158	29	Snap-8266	2567337	1.009	1.67725
30	Snap-8267	2567337	60.523	30	Snap-8268	2567337	0.02	0.033045
31	Snap-8269	2567337	61.198	31	Snap-8270	2567337	0.536	0.875846
32	Snap-8271	2567337	66.491	32	Snap-8272	2567337	1.902	2.860538
33	Snap-8273	2567337	69.476	33	Snap-8274	2567337	4.826	6.946284
34	Snap-8275	2567337	72.653	34	Snap-8276	2567337	2.161	2.974413
35	Snap-8277	2567337	50.923	35	Snap-8278	2567337	1.096	2.152269
36	Snap-8279	2567337	61.831	36	Snap-8280	2567337	2.108	3.409293
37	Snap-8281	2567337	63.46	37	Snap-8282	2567337	0.005	0.007879
38	Snap-8283	2567337	53.773	38	Snap-8284	2567337	0.699	1.299909
39	Snap-8285	2567337	52.625	39	Snap-8286	2567337	2.422	4.602375
40	Snap-8287	2567337	62.417	40	Snap-8288	2567337	0.935	1.497989
41	Snap-8289	2567337	56.431	41	Snap-8290	2567337	5.693	10.08843
42	Snap-8291	2567337	54.627	42	Snap-8292	2567337	11.214	20.52831
43	Snap-8293	2567337	46.755	43	Snap-8294	2567337	8.606	18.40659
44	Snap-8295	2567337	55.1	44	Snap-8296	2567337	1.285	2.332123
45	Snap-8297	2567337	71.725	45	Snap-8298	2567337	0.224	0.312304
46	Snap-8299	2567337	64.28	46	Snap-8300	2567337	10.071	15.66739
47	Snap-8301	2567337	62.555	47	Snap-8302	2567337	4.581	7.323156
48	Snap-8303	2567337	50.187	48	Snap-8304	2567337	11.077	22.07145

49	Snap-8305	2567337	27.883	49	Snap-8306	2567337	10.762	38.59699

Figure 8 Panel F Source Data

50	Snap-8307	2567337	21.51	50	Snap-8308	2567337	6.732	31.29707
51	Snap-8309	2567337	56.578	51	Snap-8310	2567337	2.393	4.229559
52	Snap-8311	2567337	50.177	52	Snap-8312	2567337	0.567	1.13
53	Snap-8313	2567337	61.659	53	Snap-8314	2567337	0.06	0.097309
54	Snap-8315	2567337	50.683	54	Snap-8316	2567337	0.148	0.292011
55	Snap-8317	2567337	62.845	55	Snap-8318	2567337	0.035	0.055693
56	Snap-8319	2567337	62.249	56	Snap-8320	2567337	0.61	0.979935
57	Snap-8321	2567337	74.007	57	Snap-8322	2567337	1.633	2.206548
58	Snap-8323	2567337	52.621	58	Snap-8324	2567337	0.08	0.152031
59	Snap-8325	2567337	46.82	59	Snap-8326	2567337	0.562	1.200342
60	Snap-8327	2567337	67.84	60	Snap-8328	2567337	2.796	4.121462
61	Snap-8329	2567337	62.338	61	Snap-8330	2567337	0.461	0.739517
62	Snap-8331	2567337	56.487	62	Snap-8332	2567337	0.56	0.991379
63	Snap-8333	2567337	57.765	63	Snap-8334	2567337	0.495	0.85692
64	Snap-8335	2567337	58.244	64	Snap-8336	2567337	0.275	0.472152
65	Snap-8337	2567337	56.256	65	Snap-8338	2567337	0.064	0.113766
								**4.753405**
								7.977678
								**0.989509**

Figure 8 Panel F Source Data

**Tumor 8**	Label	Area	%Area		Label	Area	%Area	
1	Snap-8130	2567337	48.636	1	Snap-8131	2567337	11.993	24.65869
2	Snap-8133	2567337	42.126	2	Snap-8134	2567337	8.134	19.30874
3	Snap-8135	2567337	29.894	3	Snap-8136	2567337	10.892	36.43541
4	Snap-8137	2567337	28.016	4	Snap-8138	2567337	6.245	22.29083
5	Snap-8139	2567337	45.676	5	Snap-8140	2567337	1.214	2.657851
6	Snap-8141	2567337	53.521	6	Snap-8142	2567337	0.096	0.179369
7	Snap-8143	2567337	58.142	7	Snap-8144	2567337	1.574	2.707165
8	Snap-8145	2567337	57.561	8	Snap-8146	2567337	0.141	0.244958
9	Snap-8147	2567337	46.071	9	Snap-8148	2567337	1.289	2.797855
10	Snap-8149	2567337	38.651	10	Snap-8150	2567337	3.658	9.464179
11	Snap-8151	2567337	44.779	11	Snap-8152	2567337	0.939	2.096965
12	Snap-8153	2567337	46.212	12	Snap-8154	2567337	0.731	1.58184
13	Snap-8155	2567337	37.089	13	Snap-8156	2567337	0.586	1.579983
14	Snap-8157	2567337	43.863	14	Snap-8158	2567337	0.219	0.499282
15	Snap-8159	2567337	57.99	15	Snap-8160	2567337	1.187	2.046905
16	Snap-8161	2567337	40.899	16	Snap-8162	2567337	4.704	11.5015
17	Snap-8163	2567337	44.675	17	Snap-8164	2567337	0.05	0.111919
18	Snap-8165	2567337	44.175	18	Snap-8166	2567337	5.84E-04	0.001323
19	Snap-8167	2567337	57.148	19	Snap-8168	2567337	0.594	1.039406
20	Snap-8169	2567337	43.123	20	Snap-8170	2567337	5.237	12.14433
21	Snap-8171	2567337	48.526	21	Snap-8172	2567337	0.154	0.317356
22	Snap-8173	2567337	40.531	22	Snap-8174	2567337	5.06E-04	0.001249
23	Snap-8175	2567337	41.37	23	Snap-8176	2567337	1.95E-04	0.000471
24	Snap-8177	2567337	36.514	24	Snap-8178	2567337	0.788	2.158076
25	Snap-8179	2567337	48.118	25	Snap-8180	2567337	0.041	0.085207
26	Snap-8181	2567337	20.134	26	Snap-8182	2567337	11.386	56.55111
27	Snap-8183	2567337	46.16	27	Snap-8184	2567337	0.057	0.123484
28	Snap-8185	2567337	40.81	28	Snap-8186	2567337	0.391	0.958099
29	Snap-8187	2567337	41.027	29	Snap-8188	2567337	0.017	0.041436
30	Snap-8189	2567337	39.261	30	Snap-8190	2567337	0.119	0.3031
31	Snap-8191	2567337	41.942	31	Snap-8192	2567337	0.026	0.06199
32	Snap-8193	2567337	51.817	32	Snap-8194	2567337	0.363	0.700542
33	Snap-8195	2567337	67.51	33	Snap-8196	2567337	0.958	1.419049
34	Snap-8197	2567337	40.582	34	Snap-8198	2567337	0.469	1.155685
35	Snap-8199	2567337	47.354	35	Snap-8200	2567337	0.045	0.095029
36	Snap-8201	2567337	37.328	36	Snap-8202	2567337	0.003	0.008037
37	Snap-8203	2567337	60.276	37	Snap-8204	2567337	0.296	0.491074
38	Snap-8205	2567337	77.793	38	Snap-8206	2567337	2.365	3.040119
39	Snap-8207	2567337	66.208	39	Snap-8208	2567337	5.84E-04	0.000883
								**5.66309**
								11.6735
								**1.869256**

Figure 8 Panel F Source Data

Average between all fields of all tumors

1	3	7	8	2	4	5	6
2.164321	0.088413	26.90992	24.65869	4.704935	0.038913	13.90331	34.67608
37.62082	0.055543	8.271249	19.30874	4.774327	1.678019	28.93839	14.60083
30.43883	9.929133	0.752055	36.43541	3.739246	1.607981	40.57493	8.897632
36.40363	6.3059	4.943942	22.29083	9.418337	0.595537	34.82574	6.936558
26.77575	0.051161	4.111989	2.657851	4.706569	0.074787	15.39779	1.89759
21.88985	0.551402	5.367806	0.179369	1.040542	0.061382	11.52232	1.034757
0.053529	3.6359	15.68873	2.707165	1.682662	0.094916	6.888675	12.20073
0.014442	0.106984	10.99734	0.244958	2.833581	0.00742	2.290243	1.31441
17.20465	0.513067	1.731504	2.797855	1.724821	1.267801	7.517775	2.328529
11.51684	0.03566	0.653451	9.464179	8.099339	0.455745	31.44719	7.240736
18.86719	0.526041	0.486626	2.096965	2.174704	0.358078	28.15748	0.45585
9.278611	0.193435	0.276608	1.58184	0.705966	1.271887	1.946983	0.57131
0.785224	0.431471	2.473888	1.579983	0.168043	0.245477	3.398426	2.860737
1.800388	0.013255	0.515035	0.499282	0.187269	0.092863	0.915436	17.65692
10.40023	0.26585	0.017917	2.046905	0.619518	0.035086	1.494245	19.15717
54.20152	0.329528	0.81853	11.5015	0.124642	0.302561	5.700342	2.131204
0.008663	0.609181	0.003295	0.111919	0.06318	2.11206	0.156971	0.233257
20.76319	0.648225	0.001542	0.001323	0.135746	11.97817	1.625468	2.074696
8.089016	0.340606	2.766364	1.039406	1.079711	3.91955	1.838984	0.900648
0.691001	0.610975	0.084803	12.14433	1.579392	4.636814	0.747537	0.321761
6.658514	12.81876	0.188189	0.317356	0.230917	0.163655	1.747062	1.718689
0.257874	0.070445	1.020259	0.001249	0.086162	0.035521	0.415714	1.161044
6.033658	0.316965	1.214746	0.000471	0.06103	0.2969	1.331696	3.380703
1.267718	0.083159	1.359198	2.158076	8.048607	0.08729	0.821918	10.4771
1.088667	0.399318	2.358516	0.085207	7.818219	0.051308	3.324372	7.216855
0.179996	3.809244	1.150939	56.55111	0.256447	3.080351	2.144445	1.577937
0.18739	1.026462	1.015477	0.123484	2.651923	0.279417	0.339191	2.940686
0.698551	0.324056	1.191872	0.958099	0.466499	3.128881	4.766032	0.368336
8.439586	4.90067	1.67725	0.041436	1.998466	0.577965	1.002298	2.292856
4.008696	7.288841	0.033045	0.3031	3.649988	4.847666	1.148907	0.076996
0.003855	0.080762	0.875846	0.06199	0.210201	6.276589	0.391106	0.184601
0.161305	0.423924	2.860538	0.700542	0.005501	1.864418	14.37748	1.444082
0.095238	4.470332	6.946284	1.419049	0.025029	0.022973	1.836726	0.413142
23.79764	0.356369	2.974413	1.155685	0.121962	1.291461	3.351887	0.544099
0.049192	4.463825	2.152269	0.095029	0.034206	0.415109	14.84048	3.410061
0.008777	2.103965	3.409293	0.008037	1.347755	0.062184	10.72142	0.63491
38.33768	0.083576	0.007879	0.491074	0.021539	0.545701	7.08604	0.309331
6.889871	0.205048	1.299909	3.040119	0.367246	0.1191	0.291354	0.220195
3.803512	4.516315	4.602375	0.000883	2.025374	1.11672	1.026569	6.014497
4.130232	0.818525	1.497989	5.66309	2.632401	0.098835	1.393107	18.92675
31.3548	5.947934	10.08843	11.6735	0.427032	0.756363	6.833881	0.862857
0.018639	11.84831	20.52831	1.869256	0.188895	4.670866	6.176359	11.84726
11.32737	9.840605	18.40659	1.183585	0.297154	2.463441	27.08457	2.200514
3.57989	0.099295	2.332123	6.748638	0.066346	0.548771	3.369868	0.079229
21.422	6.722689	0.312304	9.280054	0.305691	0.898958	1.319518	5.407322
0.002346	1.838011	15.66739	4.121462	2.384141	3.734953	0.91896	0.055969
9.581426	6.373126	7.323156	0.739517	4.894715	3.772141	0.015511	3.855575

42.55417	0.543797	22.07145	0.991379	8.551354	2.307902	1.329099	1.384464

Figure 8 Panel F Source Data

32.81682	0.048826	38.59699	0.85692	2.607903	3.637129	0.310379	0.40778
14.40309	2.389079	31.29707	0.472152	0.272319	12.76362	0.661262	0.390261
3.011025	3.414779	4.229559	0.113766	0.376908	19.31971	0.171492	3.612517
0.183001	0.487826	1.13	4.753405	0.077011	9.833091	0.415036	0.00076
0.214332	1.394443	0.097309	7.977678	2.053744	8.124134	2.947029	4.521918
0.46282	0.002634	0.292011	0.989509	0.162502	9.729304	0.777382	3.404783
0.095054	4.892013	0.055693	32.66336	0.176949	15.32153	1.310518	3.836907
0.562005	0.521822	0.979935	5.830783	0.251474	14.51175	0.953452	3.876267
3.188341	0.982403	2.206548	1.200342	0.974982	4.578411	15.25984	7.498181
0.009247	1.480961	0.152031		3.868578	1.826792	1.370177	11.04998
			**5.804968**	1.227127	2.01214	1.258573	0.437323
			9.984356	0.387468	1.581668	0.937308	1.145381
	231		**0.656922**	1.879061	0.065575	0.562558	4.444659
				1.462217	0.060207	0.290519	6.268351
				1.045318	2.062162	0.353897	0.809241
				0.699602	0.048637		
							**3.78174**
				254			6.311
							**0.395987**
							**p**
							**0.003674**
							**

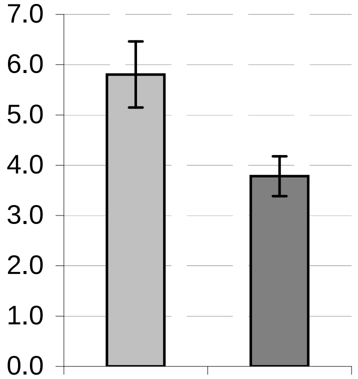

Figure 8 Panel F Source Data

Slice		%Area		Slice		%Area		
GBM 8 1_1_c1.tif	Control	17.9	51	GBM 8 1_1_c1.tif	Control	17.9	50.615	
GBM 8 1_1_c2.tif	Control	9.06		GBM 8 1-02_c1.tif	Control	24.01	43.898	
GBM 8 1-02_c1.tif	Control	24.01	44	GBM 8 1-03_c1.tif	Control	22.59	15.405	
GBM 8 1-02_c2.tif	Control	10.54		GBM 8 1-04_c1.tif	Control	17.11	12.683	
GBM 8 1-03_c1.tif	Control	22.59	15	GBM 8 1-05_c1.tif	Control	11.28	40.514	
GBM 8 1-03_c2.tif	Control	3.48		GBM 8 1-06_c1.tif	Control	25.78	27.192	
GBM 8 1-04_c1.tif	Control	17.11	13	GBM 8 1-07_c1.tif	Control	26.02	25.096	
GBM 8 1-04_c2.tif	Control	2.17		GBM 8 1-08_c1.tif	Control	12.17	6.6557	
GBM 8 1-05_c1.tif	Control	11.28	41	GBM 8 1-09_c1.tif	Control	19.14	1.3584	
GBM 8 1-05_c2.tif	Control	4.57		GBM 8 1-10_c1.tif	Control	30.25	5.3884	
GBM 8 1-06_c1.tif	Control	25.78	27	GBM 8 1-11_c1.tif	Control	24.37	20.312	
GBM 8 1-06_c2.tif	Control	7.01		GBM 8 1-12_c1.tif	Control	12.46	20.305	
GBM 8 1-07_c1.tif	Control	26.02	25	GBM 8 1-13_c1.tif	Control	2.97	82.492	27.07
GBM 8 1-07_c2.tif	Control	6.53		GBM 8 2-01_c1.tif	miR-10b-i	20.77	8.5701	
GBM 8 1-08_c1.tif	Control	12.17	6.7	GBM 8 2-02_c1.tif	miR-10b-i	29.05	13.632	
GBM 8 1-08_c2.tif	Control	0.81		GBM 8 2-03_c1.tif	miR-10b-i	23.94	7.6859	
GBM 8 1-09_c1.tif	Control	19.14	1.4	GBM 8 2-04_c1.tif	miR-10b-i	30.55	17.938	
GBM 8 1-09_c2.tif	Control	0.26		GBM 8 2-05_c1.tif	miR-10b-i	22.17	8.6152	
GBM 8 1-10_c1.tif	Control	30.25	5.4	GBM 8 2-06_c1.tif	miR-10b-i	27.66	0.1085	
GBM 8 1-10_c2.tif	Control	1.63		GBM 8 2-07_c1.tif	miR-10b-i	11.57	4.8401	
GBM 8 1-11_c1.tif	Control	24.37	20	GBM 8 2-08_c1.tif	miR-10b-i	21.28	7.0019	
GBM 8 1-11_c2.tif	Control	4.95		GBM 8 2-09_c1.tif	miR-10b-i	14.52	14.738	
GBM 8 1-12_c1.tif	Control	12.46	20	GBM 8 2-10_c1.tif	miR-10b-i	1.48	62.838	14.6
GBM 8 1-12_c2.tif	Control	2.53		GBM 8 3-01_c1.tif	Control	18.89	9.2112	
GBM 8 1-13_c1.tif	Control	2.97	82	GBM 8 3-02_c1.tif	Control	20.86	34.995	
GBM 8 1-13_c2.tif	Control	2.45		GBM 8 3-03_c1.tif	Control	24.86	1.8504	
GBM 8 2-01_c1.tif	miR-10b-i	20.77	8.6	GBM 8 3-04_c1.tif	Control	24.7	20.769	
GBM 8 2-01_c2.tif	miR-10b-i	1.78		GBM 8 3-05_c1.tif	Control	23.41	25.032	
GBM 8 2-02_c1.tif	miR-10b-i	29.05	14	GBM 8 3-06_c1.tif	Control	29.15	20.309	
GBM 8 2-02_c2.tif	miR-10b-i	3.96		GBM 8 3-07_c1.tif	Control	12.19	24.774	
GBM 8 2-03_c1.tif	miR-10b-i	23.94	7.7	GBM 8 3-08_c1.tif	Control	24.31	1.1518	
GBM 8 2-03_c2.tif	miR-10b-i	1.84		GBM 8 3-09_c1.tif	Control	24.06	5.5694	
GBM 8 2-04_c1.tif	miR-10b-i	30.55	18	GBM 8 3-10_c1.tif	Control	11.25	13.333	15.7
GBM 8 2-04_c2.tif	miR-10b-i	5.48		GBM 8 4-01_c1.tif	miR-10b-i	19.53	10.804	
GBM 8 2-05_c1.tif	miR-10b-i	22.17	8.6	GBM 8 4-02_c1.tif	miR-10b-i	8.19	44.933	
GBM 8 2-05_c2.tif	miR-10b-i	1.91		GBM 8 4-03_c1.tif	miR-10b-i	14.16	64.901	
GBM 8 2-06_c1.tif	miR-10b-i	27.66	0.1	GBM 8 4-04_c1.tif	miR-10b-i	11.26	11.368	
GBM 8 2-06_c2.tif	miR-10b-i	0.03		GBM 8 4-05_c1.tif	miR-10b-i	17.33	11.483	
GBM 8 2-07_c1.tif	miR-10b-i	11.57	4.8	GBM 8 4-06_c1.tif	miR-10b-i	30.74	1.3663	
GBM 8 2-07_c2.tif	miR-10b-i	0.56		GBM 8 4-07_c1.tif	miR-10b-i	30	32.9	
GBM 8 2-08_c1.tif	miR-10b-i	21.28	7	GBM 8 4-09_c1.tif	miR-10b-i	7.41	20.243	
GBM 8 2-08_c2.tif	miR-10b-i	1.49		GBM 8 4-10_c1.tif	miR-10b-i	15.47	26.568	
GBM 8 2-09_c1.tif	miR-10b-i	14.52	15	GBM 8 4-11_c1.tif	miR-10b-i	25.67	23.023	
GBM 8 2-09_c2.tif	miR-10b-i	2.14		GBM 8 4-12_c1.tif	miR-10b-i	28.49	13.97	
GBM 8 2-10_c1.tif	miR-10b-i	1.48	63	GBM 8 4-13_c1.tif	miR-10b-i	20.66	62.972	27.04
GBM 8 2-10_c2.tif	miR-10b-i	0.93		GBM 8 5-01_c1.tif	miR-10b-i	17.69	13.51	
GBM 8 3-01_c1.tif	Control	18.89	9.2	GBM 8 5-02_c1.tif	miR-10b-i	23.48	7.5809	
GBM 8 3-01_c2.tif	Control	1.74		GBM 8 5-03_c1.tif	miR-10b-i	13.45	83.569	

Figure 8 Panel F Source Data

Slice		%Area						
GBM 8 3-02_c1.tif	Control	20.86	35	GBM 8 5-04_c1.tif	miR-10b-i	19.81	7.4205	
GBM 8 3-02_c2.tif	Control	7.3		GBM 8 5-05_c1.tif	miR-10b-i	16.11	2.7933	22.97
GBM 8 3-03_c1.tif	Control	24.86	1.9	GBM 8 6-01_c2.tif	miR-10b-i	12.43	39.984	
GBM 8 3-03_c2.tif	Control	0.46		GBM 8 6-02_c1.tif	miR-10b-i	26.16	32.531	
GBM 8 3-04_c1.tif	Control	24.7	21	GBM 8 6-03_c1.tif	miR-10b-i	13.71	39.096	
GBM 8 3-04_c2.tif	Control	5.13		GBM 8 6-04_c1.tif	miR-10b-i	19.72	2.8398	
GBM 8 3-05_c1.tif	Control	23.41	25	GBM 8 6-05_c1.tif	miR-10b-i	17.08	10.422	
GBM 8 3-05_c2.tif	Control	5.86		GBM 8 6-06_c1.tif	miR-10b-i	19.26	8.4112	18.06
GBM 8 3-06_c1.tif	Control	29.15	20	GBM 8 7-01_c1.tif	Control	25.13	7.6801	
GBM 8 3-06_c2.tif	Control	5.92		GBm 8 7 02_c1.tif	Control	9.12	31.469	
GBM 8 3-07_c1.tif	Control	12.19	25	GBm 8 7 03_c1.tif	Control	4.12	100	46.38
GBM 8 3-07_c2.tif	Control	3.02		GBM 8 8-01_c1.tif	Control	14.59	60.178	
GBM 8 3-08_c1.tif	Control	24.31	1.2	GBM 8 8-02_c1.tif	Control	15.08	67.175	
GBM 8 3-08_c2.tif	Control	0.28		GBM 8 8-03_c1.tif	Control	5.18	58.301	
GBM 8 3-09_c1.tif	Control	24.06	5.6	GBM 8 8-04_c1.tif	Control	19.97	21.582	
GBM 8 3-09_c2.tif	Control	1.34		GBM 8 8-05_c1.tif	Control	14.41	91.672	
GBM 8 3-10_c1.tif	Control	11.25	13	GBM 8 8-06_c1.tif	Control	26.36	2.5417	50.24
GBM 8 3-10_c2.tif	Control	1.5						
GBM 8 4-01_c1.tif	miR-10b-i	19.53	11					
GBM 8 4-01_c2.tif	miR-10b-i	2.11						
GBM 8 4-02_c1.tif	miR-10b-i	8.19	45					
GBM 8 4-02_c2.tif	miR-10b-i	3.68						
GBM 8 4-03_c1.tif	miR-10b-i	14.16	65					
GBM 8 4-03_c2.tif	miR-10b-i	9.19						
GBM 8 4-04_c1.tif	miR-10b-i	11.26	11					
GBM 8 4-04_c2.tif	miR-10b-i	1.28						
GBM 8 4-05_c1.tif	miR-10b-i	17.33	11					
GBM 8 4-05_c2.tif	miR-10b-i	1.99						
GBM 8 4-06_c1.tif	miR-10b-i	30.74	1.4					
GBM 8 4-06_c2.tif	miR-10b-i	0.42						
GBM 8 4-07_c1.tif	miR-10b-i	30	33					
GBM 8 4-07_c2.tif	miR-10b-i	9.87						
GBM 8 4-09_c1.tif	miR-10b-i	7.41	20					
GBM 8 4-09_c2.tif	miR-10b-i	1.5						
GBM 8 4-10_c1.tif	miR-10b-i	15.47	27					
GBM 8 4-10_c2.tif	miR-10b-i	4.11						
GBM 8 4-11_c1.tif	miR-10b-i	25.67	23					
GBM 8 4-11_c2.tif	miR-10b-i	5.91						
GBM 8 4-12_c1.tif	miR-10b-i	28.49	14					
GBM 8 4-12_c2.tif	miR-10b-i	3.98						
GBM 8 4-13_c1.tif	miR-10b-i	20.66	63					
GBM 8 4-13_c2.tif	miR-10b-i	13.01						
GBM 8 5-01_c1.tif	miR-10b-i	17.69	14					
GBM 8 5-01_c2.tif	miR-10b-i	2.39						
GBM 8 5-02_c1.tif	miR-10b-i	23.48	7.6					
GBM 8 5-02_c2.tif	miR-10b-i	1.78						

Figure 8 Panel F Source Data

Slice	%Area	
GBM 8 5-0 miR-10b-i	13.45	83.56877
GBM 8 5-0 miR-10b-i	11.24	
GBM 8 5-0 miR-10b-i	19.81	7.420495
GBM 8 5-0 miR-10b-i	1.47	
GBM 8 5-0 miR-10b-i	16.11	2.793296
GBM 8 5-0 miR-10b-i	0.45	
GBM 8 6-0 miR-10b-i	12.43	39.98391
GBM 8 6-0 miR-10b-i	4.97	
GBM 8 6-0 miR-10b-i	26.16	32.53058
GBM 8 6-0 miR-10b-i	8.51	
GBM 8 6-0 miR-10b-i	13.71	39.09555
GBM 8 6-0 miR-10b-i	5.36	
GBM 8 6-0 miR-10b-i	19.72	2.839757
GBM 8 6-0 miR-10b-i	0.56	
GBM 8 6-0 miR-10b-i	17.08	10.42155
GBM 8 6-0 miR-10b-i	1.78	
GBM 8 6-0 miR-10b-i	19.26	8.411215
GBM 8 6-0 miR-10b-i	1.62	
GBM 8 7-0 Control	25.13	7.680064
GBM 8 7-0 Control	1.93	
GBm 8 7 0 Control	9.12	31.4693
GBm 8 7 0 Control	2.87	
GBm 8 7 0 Control	4.12	100
GBm 8 7 0 Control	4.12	
GBM 8 8-0 Control	14.59	60.1782
GBM 8 8-0 Control	8.78	
GBM 8 8-0 Control	15.08	67.17507
GBM 8 8-0 Control	10.13	
GBM 8 8-0 Control	5.18	58.30116
GBM 8 8-0 Control	3.02	
GBM 8 8-0 Control	19.97	21.58237
GBM 8 8-0 Control	4.31	
GBM 8 8-0 Control	14.41	91.67245
GBM 8 8-0 Control	13.21	
GBM 8 8-0 Control	26.36	2.54173
GBM 8 8-0 Control	0.67	

Figure 8 Panel F Source Data

Control	anti-miR-10b
50.61453	8.570053
43.89838	13.63167
15.40505	7.685881
12.68264	17.93781
40.51418	8.615246
27.19162	0.10846
25.09608	4.840104
6.655711	7.00188
1.358412	14.73829
5.38843	62.83784
20.31186	10.80389
20.30498	44.93284
82.49158	64.90113
9.211223	11.36767
34.99521	11.48298
1.850362	1.366298
20.76923	32.9
25.03204	20.24291
20.30875	26.56755
24.77441	23.02298
1.151789	13.96981
5.56941	62.97193
13.33333	13.51046
7.680064	7.58092
31.4693	83.56877
100	7.420495
60.1782	2.793296
67.17507	39.98391
58.30116	32.53058
21.58237	39.09555
91.67245	2.839757
2.54173	10.42155
	8.411215
29.67217	21.77739
26.93727	21.25819
4.761882	3.700575

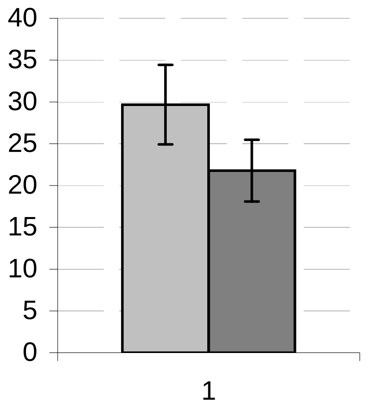

p

0.096829

Figure 8 Panel F Source Data

CC3

**Tumor 1**	Area	% area		Area	% area	
1DAPI1	77.284	49.341	CC3 Tumor1.tif	156.632	0.829	1.680144
1DAPI2	80.706	51.526	CC3 Tumor12.tif	156.632	1.01	1.960175
1DAPI3	156.632	51.593	CC3 Tumor13.tif	156.632	1.114	2.159208
1DAPI4	156.632	48.408	CC3 Tumor14.tif	156.632	0.655	1.353082
1DAPI5	156.632	51.584	CC3 Tumor15.tif	156.632	0.558	1.081731
1DAPI6	156.632	50.523	CC3 Tumor16.tif	156.632	0.637	1.260812
1DAPI7	156.632	58.433	CC3 Tumor17.tif	156.632	0.813	1.391337
1DAPI8	156.632	57.708	CC3 Tumor18.tif	156.632	0.428	0.741665
1DAPI9	156.632	51.359	CC3 Tumor19.tif	156.632	0.187	0.364104
1DAPI10	12.345	7.881	CC3 Tumor110.tif	156.632	0.056	0.71057
1DAPI11	45.703	29.179	CC3 Tumor111.tif	156.632	0.279	0.956167
1DAPI12	156.632	58.232	CC3 Tumor112.tif	156.632	0.314	0.539222
1DAPI13	156.632	64.077	CC3 Tumor113.tif	156.632	0.29	0.45258
1DAPI14	86.138	54.994	CC3 Tumor114.tif	156.632	0.37	0.672801
1DAPI15	89.407	57.081	CC3 Tumor115.tif	156.632	0.829	1.452322
1DAPI16	156.632	61.914	CC3 Tumor116.tif	156.632	1.028	1.660368
1DAPI17	85.904	54.845	CC3 Tumor117.tif	156.632	0.509	0.92807
1DAPI18	100.25	64.004	CC3 Tumor118.tif	156.632	1.245	1.945191
1DAPI19	156.632	68.993	CC3 Tumor119.tif	156.632	0.683	0.989956
1DAPI20	53.862	34.388	CC3 Tumor120.tif	156.632	0.109	0.316971
1DAPI21	156.632	78.286	CC3 Tumor121.tif	156.632	0.884	1.129193
1DAPI22	156.632	71.254	CC3 Tumor122.tif	156.632	1.461	2.050411
1DAPI23	156.632	42.96	CC3 Tumor123.tif	156.632	0.419	0.975326
1DAPI24	156.632	61.122	CC3 Tumor124.tif	156.632	1.185	1.938745
1DAPI25	156.632	63.189	CC3 Tumor125.tif	156.632	0.844	1.335676
1DAPI26	156.632	59.8	CC3 Tumor126.tif	156.632	1.237	2.068562
1DAPI27	156.632	66.777	CC3 Tumor127.tif	156.632	0.944	1.41366
1DAPI28	156.632	56.078	CC3 Tumor128.tif	156.632	0.61	1.087771
1DAPI29	156.632	53.046	CC3 Tumor129.tif	156.632	0.234	0.441127
1DAPI30	156.632	56.717	CC3 Tumor130.tif	156.632	0.765	1.348802
1DAPI31	156.632	62.071	CC3 Tumor131.tif	156.632	0.725	1.168017
1DAPI32	156.632	47.367	CC3 Tumor132.tif	156.632	0.618	1.304706
1DAPI33	156.632	58.109	CC3 Tumor133.tif	156.632	0.936	1.610766
1DAPI34	156.632	56.067	CC3 Tumor134.tif	156.632	0.344	0.613552
1DAPI35	156.632	68.245	CC3 Tumor135.tif	156.632	0.847	1.241117
1DAPI36	156.632	60.044	CC3 Tumor136.tif	156.632	0.716	1.192459
1DAPI37	156.632	71.012	CC3 Tumor137.tif	156.632	3.456	4.866783
1DAPI38	156.632	73.472	CC3 Tumor138.tif	156.632	1.488	2.025261
1DAPI39	156.632	76.883	CC3 Tumor139.tif	156.632	1.609	2.09279
1DAPI40	156.632	49.992	CC3 Tumor140.tif	156.632	0.711	1.422228
1DAPI41	156.632	41.72	CC3 Tumor141.tif	156.632	0.413	0.989933
1DAPI42	156.632	62.254	CC3 Tumor142.tif	156.632	0.429	0.689112
1DAPI43	156.632	56.639	CC3 Tumor143.tif	156.632	0.792	1.39833
1DAPI44	156.632	75.472	CC3 Tumor144.tif	156.632	2.539	3.364162
1DAPI45	156.632	62.485	CC3 Tumor145.tif	156.632	1.063	1.701208
1DAPI46	156.632	66.288	CC3 Tumor146.tif	156.632	1.735	2.617367

Figure 8 Panel F Source Data

**Tumor 1**	Area	% area		Area	% area	
1DAPI47	156.632	72.778	CC3 Tumo	156.632	3.83	5.262579
1DAPI48	156.632	63.66	CC3 Tumo	156.632	0.789	1.239397
1DAPI50	156.632	77.105	CC3 Tumo	156.632	0.771	0.999935
1DAPI51	156.632	71.608	CC3 Tumo	156.632	1.011	1.411853
1DAPI52	156.632	79.248	CC3 Tumo	156.632	1.672	2.109832
1DAPI53	156.632	75.754	CC3 Tumo	156.632	2.954	3.899464
1DAPI54	156.632	74.129	CC3 Tumo	156.632	1.196	1.613404
						**1.53283**
						0.986081
						**0.135449**

control				anti-miR-10b			
1	3	7	8	2	4	5	6
1.53283	1.590371	2.142785	1.520238	1.853704	4.357311	1.938964	4.078324
0.135449	0.157426	0.121177	0.110593	0.203577	0.292285	0.207957	0.47357
			1.696556				3.057076
			0.299048				1.34559
			0.149524				0.672795
							**p**
							0.047907
							*

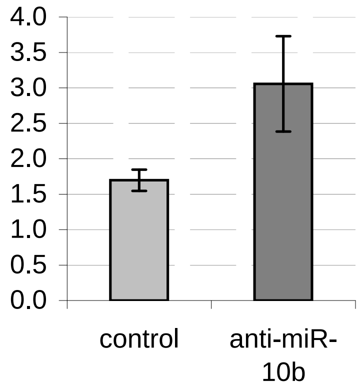

Figure 8 Panel F Source Data

**Tumor 2**	Area	% area	Label	Area	%Area	
CC3 Tumo	1443520	50.705	CC3 Tumo	1443520	0.595	1.173454
CC3 Tumo	1443520	28.45	CC3 Tumo	1443520	0.373	1.311072
CC3 Tumo	1443520	45.191	CC3 Tumo	1443520	0.763	1.688389
CC3 Tumo	1443520	44.793	CC3 Tumo	1443520	2.079	4.64135
CC3 Tumo	1443520	55.583	CC3 Tumo	1443520	2.108	3.792526
CC3 Tumo	1443520	56.726	CC3 Tumo	1443520	0.419	0.738638
CC3 Tumo	1443520	39.989	CC3 Tumo	1443520	2.726	6.816875
CC3 Tumo	1443520	43.778	CC3 Tumo	1443520	0.501	1.14441
CC3 Tumo	1443520	43.469	CC3 Tumo	1443520	0.776	1.78518
CC3 Tumo	1443520	53.852	CC3 Tumo	1443520	0.609	1.130877
CC3 Tumo	1443520	53.922	CC3 Tumo	1443520	0.303	0.561923
CC3 Tumo	1443520	34.627	CC3 Tumo	1443520	0.637	1.839605
CC3 Tumo	1443520	15.177	CC3 Tumo	1443520	0.277	1.82513
CC3 Tumo	1443520	37.08	CC3 Tumo	1443520	0.615	1.658576
CC3 Tumo	1443520	40.998	CC3 Tumo	1443520	0.527	1.285429
CC3 Tumo	1443520	64.035	CC3 Tumo	1443520	0.293	0.457562
CC3 Tumo	1443520	59.708	CC3 Tumo	1443520	0.81	1.356602
CC3 Tumo	1443520	30.625	CC3 Tumo	1443520	0.341	1.113469
CC3 Tumo	1443520	54.188	CC3 Tumo	1443520	0.52	0.959622
CC3 Tumo	1443520	57.382	CC3 Tumo	1443520	1.233	2.148757
CC3 Tumo	1443520	65.427	CC3 Tumo	1443520	0.376	0.574686
CC3 Tumo	1443520	36.405	CC3 Tumo	1443520	0.057	0.156572
CC3 Tumo	1443520	17.523	CC3 Tumo	1443520	0.434	2.476745
CC3 Tumo	1443520	34.741	CC3 Tumo	1443520	0.458	1.318327
CC3 Tumo	1443520	32.903	CC3 Tumo	1443520	0.225	0.683828
CC3 Tumo	1443520	47.251	CC3 Tumo	1443520	1.133	2.397833
CC3 Tumo	1443520	51.762	CC3 Tumo	1443520	1.803	3.48325
CC3 Tumo	1443520	28.571	CC3 Tumo	1443520	0.818	2.863043
CC3 Tumo	1443520	31.596	CC3 Tumo	1443520	0.402	1.272313
CC3 Tumo	1443520	56.996	CC3 Tumo	1443520	0.769	1.349217
CC3 Tumo	1443520	57.445	CC3 Tumo	1443520	1.485	2.585081
CC3 Tumo	1443520	42.388	CC3 Tumo	1443520	0.521	1.229121
CC3 Tumo	1443520	30.527	CC3 Tumo	1443520	0.325	1.064631
CC3 Tumo	1443520	41.773	CC3 Tumo	1443520	0.771	1.84569
CC3 Tumo	1443520	50.07	CC3 Tumo	1443520	2.053	4.10026
CC3 Tumo	1443520	54.952	CC3 Tumo	1443520	0.612	1.113699
CC3 Tumo	1443520	73.491	CC3 Tumo	1443520	1.495	2.034263
CC3 Tumo	1443520	52.084	CC3 Tumo	1443520	1.552	2.979802
CC3 Tumo	1443520	29.199	CC3 Tumo	1443520	0.411	1.407582
CC3 Tumo	1443520	19.296	CC3 Tumo	1443520	0.344	1.782753
						**1.853704**
						1.287535
						**0.203577**

Figure 8 Panel F Source Data

**Tumor 3**	Area	%Area	Label	Area	%Area	
CC3 Tumo	1443520	54.02	CC3 Tumo	1443520	0.324	0.59977786
CC3 Tumo	1443520	51.823	CC3 Tumo	1443520	0.15	0.28944677
CC3 Tumo	1443520	53.978	CC3 Tumo	1443520	0.209	0.38719478
CC3 Tumo	1443520	47.888	CC3 Tumo	1443520	0.635	1.32601069
CC3 Tumo	1443520	62.095	CC3 Tumo	1443520	0.342	0.55076898
CC3 Tumo	1443520	56.909	CC3 Tumo	1443520	0.569	0.99984185
CC3 Tumo	1443520	27.404	CC3 Tumo	1443520	0.266	0.97066122
CC3 Tumo	1443520	66.742	CC3 Tumo	1443520	1.239	1.85640227
CC3 Tumo	1443520	59.577	CC3 Tumo	1443520	1.017	1.70703459
CC3 Tumo	1443520	65.145	CC3 Tumo	1443520	0.805	1.23570497
CC3 Tumo	1443520	26.169	CC3 Tumo	1443520	0.502	1.91830028
CC3 Tumo	1443520	39.493	CC3 Tumo	1443520	0.34	0.86091206
CC3 Tumo	1443520	62.271	CC3 Tumo	1443520	0.833	1.33770134
CC3 Tumo	1443520	57.638	CC3 Tumo	1443520	1.302	2.2589264
CC3 Tumo	1443520	58.833	CC3 Tumo	1443520	1.53	2.60058131
CC3 Tumo	1443520	40.009	CC3 Tumo	1443520	1.01	2.524432
CC3 Tumo	1443520	58.277	CC3 Tumo	1443520	1.678	2.87935206
CC3 Tumo	1443520	56.623	CC3 Tumo	1443520	1.555	2.74623386
CC3 Tumo	1443520	32.539	CC3 Tumo	1443520	0.883	2.71366668
CC3 Tumo	1443520	35.664	CC3 Tumo	1443520	0.549	1.53936743
CC3 Tumo	1443520	49.759	CC3 Tumo	1443520	1.885	3.78825941
CC3 Tumo	1443520	71.47	CC3 Tumo	1443520	1.24	1.7349937
CC3 Tumo	1443520	66.168	CC3 Tumo	1443520	1.529	2.31078467
CC3 Tumo	1443520	68.909	CC3 Tumo	1443520	0.541	0.78509338
CC3 Tumo	1443520	65.695	CC3 Tumo	1443520	0.244	0.37141335
CC3 Tumo	1443520	55.435	CC3 Tumo	1443520	0.372	0.67105619
CC3 Tumo	1443520	55	CC3 Tumo	1443520	0.397	0.72181818
CC3 Tumo	1443520	63.995	CC3 Tumo	1443520	0.67	1.04695679
CC3 Tumo	1443520	53.187	CC3 Tumo	1443520	0.849	1.59625472
CC3 Tumo	1443520	63.382	CC3 Tumo	1443520	0.503	0.79360071
CC3 Tumo	1443520	64.612	CC3 Tumo	1443520	0.504	0.78004086
CC3 Tumo	1443520	62.741	CC3 Tumo	1443520	0.532	0.84793038
CC3 Tumo	1443520	36.352	CC3 Tumo	1443520	1.398	3.84573063
CC3 Tumo	1443520	62.15	CC3 Tumo	1443520	1.488	2.39420756
CC3 Tumo	1443520	58.975	CC3 Tumo	1443520	1.416	2.40101738
CC3 Tumo	1443520	64.773	CC3 Tumo	1443520	1.206	1.8618869
						**1.59037117**
						0.94455462
						**0.15742577**

Figure 8 Panel F Source Data

**Tumor 4**	Area	%Area	Label	Area	%Area	
CC3 tumor	1443520	31.455	CC3 tumor	1443520	1.696	5.39183
CC3 tumor	1443520	19.056	CC3 tumor	1443520	0.884	4.638959
CC3 tumor	1443520	35.4	CC3 tumor	1443520	1.801	5.087571
CC3 tumor	1443520	36.366	CC3 tumor	1443520	2.504	6.885552
CC3 tumor	1443520	28.713	CC3 tumor	1443520	0.764	2.660816
CC3 tumor	1443520	37.102	CC3 tumor	1443520	1.282	3.455339
CC3 tumor	1443520	42.399	CC3 tumor	1443520	3.573	8.427086
CC3 tumor	1443520	49.502	CC3 tumor	1443520	2.729	5.512909
CC3 tumor	1443520	39.684	CC3 tumor	1443520	3.801	9.578168
CC3 tumor	1443520	41.217	CC3 tumor	1443520	2.509	6.087294
CC3 tumor	1443520	33.527	CC3 tumor	1443520	0.573	1.70907
CC3 tumor	1443520	33.192	CC3 tumor	1443520	1.344	4.049168
CC3 tumor	1443520	30.112	CC3 tumor	1443520	1.245	4.134564
CC3 tumor	1443520	22.82	CC3 tumor	1443520	0.657	2.879053
CC3 tumor	1443520	36.574	CC3 tumor	1443520	1.149	3.141576
CC3 tumor	1443520	46.285	CC3 tumor	1443520	2.012	4.346981
CC3 tumor	1443520	42.961	CC3 tumor	1443520	1.229	2.860734
CC3 tumor	1443520	28.682	CC3 tumor	1443520	0.596	2.077958
CC3 tumor	1443520	40.148	CC3 tumor	1443520	1.658	4.12972
CC3 tumor	1443520	39.867	CC3 tumor	1443520	2.095	5.254973
CC3 tumor	1443520	33.44	CC3 tumor	1443520	1.047	3.130981
CC3 tumor	1443520	56.909	CC3 tumor	1443520	2.891	5.08004
CC3 tumor	1443520	53.949	CC3 tumor	1443520	1.557	2.886059
CC3 tumor	1443520	52.394	CC3 tumor	1443520	0.897	1.712028
CC3 tumor	1443520	45.548	CC3 tumor	1443520	1.699	3.730131
CC3 tumor	1443520	28.36	CC3 tumor	1443520	0.767	2.704513
CC3 tumor	1443520	39.108	CC3 tumor	1443520	1.035	2.646517
CC3 tumor	1443520	42.674	CC3 tumor	1443520	2.454	5.750574
CC3 tumor	1443520	39.122	CC3 tumor	1443520	1.297	3.31527
CC3 tumor	1443520	47.902	CC3 tumor	1443520	3.741	7.809695
CC3 tumor	1443520	48.704	CC3 tumor	1443520	1.951	4.005831
CC3 tumor	1443520	29.844	CC3 tumor	1443520	1.719	5.759952
CC3 tumor	1443520	62.177	CC3 tumor	1443520	3.012	4.844235
CC3 tumor	1443520	58.526	CC3 tumor	1443520	3.33	5.689779
CC3 tumor	1443520	56.575	CC3 tumor	1443520	5.234	9.251436
CC3 tumor	1443520	40.126	CC3 tumor	1443520	3.005	7.48891
CC3 tumor	1443520	30.58	CC3 tumor	1443520	0.943	3.083715
CC3 tumor	1443520	20.64	CC3 tumor	1443520	1.131	5.479651
CC3 tumor	1443520	21.88	CC3 tumor	1443520	0.608	2.778793
CC3 tumor	1443520	24.432	CC3 tumor	1443520	0.418	1.710871
CC3 tumor	1443520	17.462	CC3 tumor	1443520	0.665	3.808269
CC3 tumor	1443520	8.93	CC3 tumor	1443520	0.397	4.445689
CC3 tumor	1443520	35.077	CC3 tumor	1443520	1.778	5.068849
CC3 tumor	1443520	54.095	CC3 tumor	1443520	3.001	5.547648
CC3 tumor	1443520	59.043	CC3 tumor	1443520	3.07	5.1996
CC3 tumor	1443520	66.515	CC3 tumor	1443520	0.731	1.099

Figure 8 Panel F Source Data

**Tumor 4**	Area	%Area	Label	Area	%Area	
CC3 tumor 4	1443520	36.537	CC3 tumor 4	1443520	0.657	1.79817719
CC3 tumor 4	1443520	39.689	CC3 tumor 4	1443520	0.403	1.01539469
						**4.35731103**
						2.02500985
						**0.292285**

Figure 8 Panel F Source Data

	Tumor 5	Area	%Area	Label	Area	%Area
CC3 Tumor5	1443520	35.166	CC3 Tumo	1443520	1.547	4.399136
CC3 Tumor5	1443520	32.871	CC3 Tumo	1443520	0.932	2.835326
CC3 Tumor5	1443520	47.521	CC3 Tumo	1443520	2.27	4.776836
CC3 Tumor5	1443520	30.499	CC3 Tumo	1443520	0.32	1.049215
CC3 Tumor5	1443520	36.499	CC3 Tumo	1443520	0.333	0.912354
CC3 Tumor5	1443520	28.065	CC3 Tumo	1443520	0.43	1.532157
CC3 Tumor5	1443520	35.609	CC3 Tumo	1443520	0.836	2.347721
CC3 Tumor5	1443520	33.674	CC3 Tumo	1443520	0.555	1.648156
CC3 Tumor5	1443520	25.264	CC3 Tumo	1443520	1.065	4.215484
CC3 Tumor5	1443520	27.64	CC3 Tumo	1443520	0.237	0.857453
CC3 Tumor5	1443520	25.421	CC3 Tumo	1443520	1.009	3.969159
CC3 Tumor5	1443520	45.29	CC3 Tumo	1443520	1.761	3.888276
CC3 Tumor5	1443520	43.125	CC3 Tumo	1443520	1.182	2.74087
CC3 Tumor5	1443520	42.902	CC3 Tumo	1443520	0.753	1.755163
CC3 Tumor5	1443520	27.253	CC3 Tumo	1443520	0.232	0.851282
CC3 Tumor5	1443520	35.968	CC3 Tumo	1443520	0.507	1.409586
CC3 Tumor5	1443520	36.538	CC3 Tumo	1443520	0.725	1.984236
CC3 Tumor5	1443520	45.698	CC3 Tumo	1443520	0.975	2.133573
CC3 Tumor5	1443520	45.118	CC3 Tumo	1443520	1.183	2.622013
CC3 Tumor5	1443520	51.688	CC3 Tumo	1443520	1.354	2.619564
CC3 Tumor5	1443520	47.379	CC3 Tumo	1443520	0.648	1.367695
CC3 Tumor5	1443520	55.955	CC3 Tumo	1443520	0.452	0.807792
CC3 Tumor5	1443520	34.542	CC3 Tumo	1443520	0.177	0.51242
CC3 Tumor5	1443520	24.698	CC3 Tumo	1443520	0.053	0.214592
CC3 Tumor5	1443520	44.143	CC3 Tumo	1443520	0.358	0.811001
CC3 Tumor5	1443520	32.903	CC3 Tumo	1443520	0.567	1.723247
CC3 Tumor5	1443520	35.768	CC3 Tumo	1443520	0.375	1.048423
CC3 Tumor5	1443520	32.717	CC3 Tumo	1443520	0.233	0.712168
CC3 Tumor5	1443520	41.589	CC3 Tumo	1443520	0.609	1.46433
CC3 Tumor5	1443520	36.53	CC3 Tumo	1443520	0.617	1.689023
CC3 Tumor5	1443520	20.343	CC3 Tumo	1443520	0.451	2.216979
CC3 Tumor5	1443520	39.191	CC3 Tumo	1443520	0.588	1.500344
CC3 Tumor5	1443520	28.097	CC3 Tumo	1443520	0.385	1.370253
						**1.938964**
						1.194622
						**0.207957**

Figure 8 Panel F Source Data

**Tumor 6**	Area	%Area	Label	Area	%Area	
Snap-6976	2567337	67.996	Snap-6977	2567337	2.722	4.003177
Snap-6978	2567337	61.796	Snap-6979	2567337	1.309	2.11826
Snap-6980	2567337	60.282	Snap-6981	2567337	0.619	1.026841
Snap-6982	2567337	62.839	Snap-6983	2567337	0.965	1.535671
Snap-6984	2567337	51.899	Snap-6985	2567337	0.748	1.441261
Snap-6986	2567337	50.422	Snap-6987	2567337	1.154	2.288684
Snap-6988	2567337	53.818	Snap-6989	2567337	1.066	1.98075
Snap-6990	2567337	38.117	Snap-6991	2567337	0.402	1.054648
Snap-6992	2567337	59.059	Snap-6993	2567337	0.864	1.462944
Snap-6994	2567337	64.988	Snap-6995	2567337	1.916	2.948237
Snap-6996	2567337	52.878	Snap-6997	2567337	0.911	1.722834
Snap-6998	2567337	47.511	Snap-6999	2567337	0.883	1.858517
Snap-7000	2567337	48.81	Snap-7001	2567337	2.339	4.792051
Snap-7002	2567337	60.907	Snap-7003	2567337	1.673	2.746811
Snap-7004	2567337	54.74	Snap-7005	2567337	1.876	3.42711
Snap-7006	2567337	76.012	Snap-7007	2567337	7.133	9.384045
Snap-7008	2567337	76.574	Snap-7009	2567337	3.133	4.091467
Snap-7010	2567337	52.639	Snap-7011	2567337	0.956	1.816144
Snap-7012	2567337	57.222	Snap-7013	2567337	1.299	2.270106
Snap-7014	2567337	43.667	Snap-7015	2567337	1.643	3.762567
Snap-7016	2567337	34.886	Snap-7017	2567337	6.003	17.20748
Snap-7018	2567337	56.455	Snap-7019	2567337	2.549	4.515101
Snap-7020	2567337	40.972	Snap-7021	2567337	1.389	3.39012
Snap-7022	2567337	45.978	Snap-7023	2567337	0.837	1.820436
Snap-7024	2567337	30.129	Snap-7025	2567337	0.9	2.987155
Snap-7026	2567337	46.266	Snap-7027	2567337	1.356	2.930878
Snap-7028	2567337	57.13	Snap-7029	2567337	0.829	1.451076
Snap-7030	2567337	41.637	Snap-7031	2567337	0.122	0.293009
Snap-7032	2567337	49.274	Snap-7033	2567337	2.26	4.586597
Snap-7034	2567337	59.088	Snap-7035	2567337	2.655	4.493298
Snap-7036	2567337	49.234	Snap-7037	2567337	2.581	5.242312
Snap-7038	2567337	29.155	Snap-7039	2567337	3.623	12.42668
Snap-7040	2567337	59.408	Snap-7041	2567337	7.699	12.95953
Snap-7042	2567337	61.489	Snap-7043	2567337	3.494	5.682317
Snap-7044	2567337	59.937	Snap-7045	2567337	1.529	2.551012
Snap-7046	2567337	45.646	Snap-7047	2567337	1.237	2.709986
Snap-7048	2567337	47.185	Snap-7049	2567337	0.796	1.686977
Snap-7050	2567337	19.642	Snap-7051	2567337	0.456	2.321556
Snap-7052	2567337	40.771	Snap-7053	2567337	0.943	2.312918
Snap-7054	2567337	55.679	Snap-7055	2567337	0.949	1.704413
Snap-7056	2567337	57.007	Snap-7057	2567337	1.919	3.366253
Snap-7058	2567337	63.587	Snap-7059	2567337	1.724	2.711246
Snap-7060	2567337	77.884	Snap-7061	2567337	3.514	4.511838
Snap-7062	2567337	67.601	Snap-7063	2567337	2.767	4.093135
Snap-7064	2567337	67.637	Snap-7065	2567337	3.465	5.122936
Snap-7066	2567337	62.655	Snap-7067	2567337	3.479	5.552629

Figure 8 Panel F Source Data

**Tumor 6**	Area	%Area	Label	Area	%Area	
Snap-7068	2567337	54.932	Snap-7069	2567337	1.009	1.836816
Snap-7070	2567337	50.888	Snap-7071	2567337	1.997	3.924304
Snap-7072	2567337	52.546	Snap-7073	2567337	1.073	2.04202
Snap-7074	2567337	32.413	Snap-7075	2567337	0.822	2.536019
Snap-7076	2567337	33.165	Snap-7077	2567337	1.225	3.693653
Snap-7078	2567337	51.588	Snap-7079	2567337	1.837	3.560906
Snap-7080	2567337	46.106	Snap-7081	2567337	4.198	9.105106
Snap-7082	2567337	58.091	Snap-7083	2567337	2.384	4.103906
Snap-7084	2567337	44.108	Snap-7085	2567337	7.944	18.01034
Snap-7086	2567337	53.281	Snap-7087	2567337	2.647	4.968
Snap-7088	2567337	42.149	Snap-7089	2567337	1.821	4.320387
						**4.078324**
						3.575376
						**0.47357**

Figure 8 Panel F Source Data

**Tumor 7**	Area	%Area	Label	Area	%Area	
Snap-7092	2567337	85.92	Snap-7093	2567337	0.527	0.613361
Snap-7094	2567337	83.94	Snap-7095	2567337	0.359	0.427686
Snap-7096	2567337	82.671	Snap-7097	2567337	1.083	1.310012
Snap-7098	2567337	90.783	Snap-7099	2567337	1.665	1.834044
Snap-7100	2567337	74.674	Snap-7101	2567337	1.469	1.967218
Snap-7102	2567337	72.763	Snap-7103	2567337	2.455	3.373968
Snap-7104	2567337	49.211	Snap-7105	2567337	1.026	2.0849
Snap-7106	2567337	68.906	Snap-7107	2567337	2.29	3.323368
Snap-7108	2567337	83.739	Snap-7109	2567337	2.205	2.633182
Snap-7110	2567337	85.637	Snap-7111	2567337	1.653	1.93024
Snap-7112	2567337	83.114	Snap-7113	2567337	0.773	0.930048
Snap-7114	2567337	80.402	Snap-7115	2567337	0.988	1.228825
Snap-7116	2567337	60.703	Snap-7117	2567337	0.788	1.298124
Snap-7118	2567337	74.293	Snap-7119	2567337	0.876	1.179115
Snap-7120	2567337	83.012	Snap-7121	2567337	1.394	1.679275
Snap-7122	2567337	83.794	Snap-7123	2567337	2.34	2.792563
Snap-7124	2567337	80.684	Snap-7125	2567337	3.527	4.371375
Snap-7126	2567337	59.892	Snap-7127	2567337	1.248	2.083751
Snap-7128	2567337	48.646	Snap-7129	2567337	1.336	2.746372
Snap-7130	2567337	74.845	Snap-7131	2567337	1.83	2.445053
Snap-7132	2567337	79.626	Snap-7133	2567337	3.033	3.809057
Snap-7134	2567337	77.088	Snap-7135	2567337	2.591	3.361094
Snap-7136	2567337	85.485	Snap-7137	2567337	0.987	1.154589
Snap-7138	2567337	68.25	Snap-7139	2567337	0.771	1.12967
Snap-7140	2567337	84.313	Snap-7141	2567337	1.108	1.314151
Snap-7142	2567337	82.6	Snap-7143	2567337	4.777	5.783293
Snap-7144	2567337	75.873	Snap-7145	2567337	2.125	2.800733
Snap-7146	2567337	75.616	Snap-7147	2567337	1.555	2.056443
Snap-7148	2567337	86.399	Snap-7149	2567337	4.775	5.526684
Snap-7150	2567337	59.956	Snap-7151	2567337	4.146	6.915071
Snap-7152	2567337	93.66	Snap-7153	2567337	3.787	4.043348
Snap-7154	2567337	84.576	Snap-7155	2567337	2.749	3.250331
Snap-7156	2567337	89.274	Snap-7157	2567337	1.366	1.530121
Snap-7158	2567337	81.847	Snap-7159	2567337	3.761	4.595159
Snap-7160	2567337	78.106	Snap-7161	2567337	0.732	0.937188
Snap-7162	2567337	67.581	Snap-7163	2567337	0.81	1.198562
Snap-7164	2567337	90.756	Snap-7165	2567337	0.382	0.420909
Snap-7166	2567337	93.44	Snap-7167	2567337	0.608	0.650685
Snap-7168	2567337	94.21	Snap-7169	2567337	0.711	0.754697
Snap-7170	2567337	97.114	Snap-7171	2567337	0.872	0.897914
Snap-7172	2567337	94.624	Snap-7173	2567337	1.292	1.365404
Snap-7174	2567337	52.275	Snap-7175	2567337	1.385	2.64945
Snap-7176	2567337	87.775	Snap-7177	2567337	1.425	1.623469
Snap-7178	2567337	84.592	Snap-7179	2567337	1.367	1.615992
Snap-7180	2567337	94.487	Snap-7181	2567337	2.188	2.315662
Snap-7182	2567337	89.813	Snap-7183	2567337	1.144	1.273758

Figure 8 Panel F Source Data

**Tumor 7**	Area	%Area	Label	Area	%Area	
Snap-7184	2567337	90.686	Snap-7185	2567337	0.389	0.428953
Snap-7186	2567337	95.394	Snap-7187	2567337	0.313	0.328113
Snap-7188	2567337	93.957	Snap-7189	2567337	0.869	0.924891
Snap-7190	2567337	88.841	Snap-7191	2567337	0.57	0.641596
Snap-7192	2567337	90.284	Snap-7193	2567337	0.576	0.637987
Snap-7194	2567337	88.823	Snap-7195	2567337	0.81	0.911926
Snap-7196	2567337	90.919	Snap-7197	2567337	1.039	1.142775
Snap-7198	2567337	90.598	Snap-7199	2567337	1.938	2.13912
Snap-7200	2567337	81.295	Snap-7201	2567337	1.954	2.403592
Snap-7202	2567337	78.551	Snap-7203	2567337	1.161	1.478021
Snap-7204	2567337	79.764	Snap-7205	2567337	1.863	2.33564
Snap-7206	2567337	76.015	Snap-7207	2567337	2.194	2.886272
Snap-7208	2567337	82.891	Snap-7209	2567337	1.648	1.988153
Snap-7210	2567337	87.983	Snap-7211	2567337	2.184	2.482298
Snap-7212	2567337	92.071	Snap-7213	2567337	1.088	1.181697
Snap-7214	2567337	90.26	Snap-7215	2567337	1.213	1.343895
Snap-7216	2567337	96.181	Snap-7217	2567337	1.634	1.69888
Snap-7218	2567337	92.336	Snap-7219	2567337	1.088	1.178305
Snap-7220	2567337	87.738	Snap-7221	2567337	1.725	1.966081
Snap-7222	2567337	83.515	Snap-7223	2567337	2.807	3.361073
Snap-7224	2567337	83.036	Snap-7225	2567337	2.684	3.232333
Snap-7226	2567337	67.998	Snap-7227	2567337	1.484	2.182417
Snap-7228	2567337	73.452	Snap-7229	2567337	1.41	1.919621
Snap-7230	2567337	80.908	Snap-7231	2567337	3.24	4.004548
Snap-7232	2567337	90.19	Snap-7233	2567337	3.359	3.72436
Snap-7234	2567337	86.424	Snap-7235	2567337	1.448	1.675461
Snap-7236	2567337	92.432	Snap-7237	2567337	1.911	2.067466
Snap-7238	2567337	89.708	Snap-7239	2567337	2.64	2.942881
Snap-7240	2567337	90.539	Snap-7241	2567337	1.725	1.905256
Snap-7242	2567337	90.137	Snap-7243	2567337	2.888	3.204012
Snap-7244	2567337	83.459	Snap-7245	2567337	3.221	3.85938
Snap-7246	2567337	79.389	Snap-7247	2567337	2.536	3.194397
Snap-7248	2567337	65.891	Snap-7249	2567337	1.873	2.842573
Snap-7250	2567337	86.994	Snap-7251	2567337	3.272	3.761179
Snap-7252	2567337	84.267	Snap-7253	2567337	1.784	2.11708
Snap-7254	2567337	72.044	Snap-7255	2567337	1.9	2.637277
Snap-7256	2567337	85.866	Snap-7257	2567337	4.09	4.763236
Snap-7258	2567337	88.15	Snap-7259	2567337	1.852	2.100964
Snap-7260	2567337	82.381	Snap-7261	2567337	1.098	1.332832
Snap-7262	2567337	86.797	Snap-7263	2567337	4.693	5.406869
Snap-7264	2567337	80.474	Snap-7265	2567337	2.307	2.866764
Snap-7266	2567337	80.08	Snap-7267	2567337	0.762	0.951548
Snap-7268	2567337	68.218	Snap-7269	2567337	1.595	2.338093
Snap-7270	2567337	80.654	Snap-7271	2567337	2.295	2.845488
Snap-7272	2567337	66.781	Snap-7273	2567337	0.492	0.736736
Snap-7274	2567337	81.468	Snap-7275	2567337	0.438	0.537634

Figure 8 Panel F Source Data

**Tumor 7**	Area	%Area	Label	Area	%Area	
Snap-7276	2567337	70.153	Snap-7277	2567337	0.555	0.791128
Snap-7278	2567337	90.994	Snap-7279	2567337	0.374	0.411016
Snap-7280	2567337	75.406	Snap-7281	2567337	2.731	3.621728
Snap-7282	2567337	82.662	Snap-7283	2567337	1.65	1.99608
Snap-7284	2567337	78.93	Snap-7285	2567337	1.736	2.199417
Snap-7286	2567337	71.884	Snap-7287	2567337	1.22	1.697179
Snap-7288	2567337	73.341	Snap-7289	2567337	1.272	1.734364
Snap-7290	2567337	76.634	Snap-7291	2567337	1.724	2.249654
Snap-7292	2567337	61.207	Snap-7293	2567337	1.498	2.447432
Snap-7294	2567337	49.106	Snap-7295	2567337	0.693	1.411233
Snap-7296	2567337	82.499	Snap-7297	2567337	1.412	1.711536
Snap-7298	2567337	84.697	Snap-7299	2567337	2.575	3.040249
Snap-7300	2567337	69.42	Snap-7301	2567337	2.69	3.874964
Snap-7302	2567337	56.291	Snap-7303	2567337	2.232	3.96511
Snap-7304	2567337	76.311	Snap-7305	2567337	0.974	1.276356
Snap-7306	2567337	82.5	Snap-7307	2567337	0.993	1.203636
Snap-7308	2567337	77.332	Snap-7309	2567337	1.242	1.606062
Snap-7310	2567337	58.212	Snap-7311	2567337	0.452	0.776472
Snap-7312	2567337	72.567	Snap-7313	2567337	0.454	0.625629
Snap-7314	2567337	86.778	Snap-7315	2567337	1.043	1.201918
Snap-7316	2567337	69.438	Snap-7317	2567337	0.318	0.457962
						**2.142785**
						1.288131
						**0.121177**

Figure 8 Panel F Source Data

**Tumor 8**	Area	%Area	Label	Area	%Area	
Snap-7318	2567337	63.948	Snap-7319	2567337	1.468	2.295615
Snap-7320	2567337	48.866	Snap-7321	2567337	0.638	1.305611
Snap-7322	2567337	50.401	Snap-7323	2567337	0.753	1.494018
Snap-7324	2567337	38.685	Snap-7325	2567337	0.495	1.279566
Snap-7326	2567337	66.542	Snap-7327	2567337	0.891	1.339004
Snap-7328	2567337	65.887	Snap-7329	2567337	1.012	1.535963
Snap-7330	2567337	50.609	Snap-7331	2567337	0.873	1.72499
Snap-7332	2567337	48.365	Snap-7333	2567337	1.397	2.888452
Snap-7334	2567337	38.537	Snap-7335	2567337	0.973	2.524846
Snap-7336	2567337	57.024	Snap-7337	2567337	0.438	0.768098
Snap-7338	2567337	59.501	Snap-7339	2567337	0.521	0.875616
Snap-7340	2567337	62.081	Snap-7341	2567337	0.234	0.376927
Snap-7342	2567337	64.297	Snap-7343	2567337	0.348	0.541238
Snap-7344	2567337	50.739	Snap-7345	2567337	0.37	0.729222
Snap-7346	2567337	36.138	Snap-7347	2567337	0.487	1.347612
Snap-7348	2567337	26.708	Snap-7349	2567337	0.164	0.614048
Snap-7350	2567337	39.383	Snap-7351	2567337	0.386	0.980118
Snap-7352	2567337	49.81	Snap-7353	2567337	0.371	0.74483
Snap-7354	2567337	25.364	Snap-7355	2567337	0.416	1.64012
Snap-7356	2567337	36.682	Snap-7357	2567337	0.6	1.63568
Snap-7358	2567337	25.353	Snap-7359	2567337	0.454	1.790715
Snap-7360	2567337	38.262	Snap-7361	2567337	0.832	2.174481
Snap-7362	2567337	34.677	Snap-7363	2567337	0.695	2.00421
Snap-7364	2567337	45.608	Snap-7365	2567337	0.628	1.376951
Snap-7366	2567337	40.243	Snap-7367	2567337	0.801	1.990408
Snap-7368	2567337	27.95	Snap-7369	2567337	0.493	1.763864
Snap-7370	2567337	38.345	Snap-7371	2567337	1.2	3.129482
Snap-7372	2567337	15.821	Snap-7373	2567337	0.079	0.499336
Snap-7374	2567337	79.632	Snap-7375	2567337	1.343	1.686508
Snap-7376	2567337	66.121	Snap-7377	2567337	1.434	2.168751
Snap-7378	2567337	49.128	Snap-7379	2567337	0.169	0.343999
Snap-7380	2567337	60.974	Snap-7381	2567337	0.925	1.51704
Snap-7382	2567337	51.863	Snap-7383	2567337	0.57	1.099049
Snap-7384	2567337	62.439	Snap-7385	2567337	1.08	1.729688
Snap-7386	2567337	52.616	Snap-7387	2567337	0.783	1.48814
Snap-7388	2567337	65.519	Snap-7389	2567337	1.108	1.691113
Snap-7390	2567337	81.1	Snap-7391	2567337	2.46	3.033292
Snap-7392	2567337	74.734	Snap-7393	2567337	1.45	1.940215
Snap-7394	2567337	44.195	Snap-7395	2567337	0.645	1.459441
Snap-7396	2567337	57.303	Snap-7397	2567337	0.306	0.534003
Snap-7398	2567337	72.15	Snap-7399	2567337	1.636	2.267498
						**1.520238**
						0.708138
						**0.110593**

Figure 8 Panel F Source Data

Tumor 1	Tumor 2	Tumor 3	Tumor 4	Tumor 5	Tumor 6	Tumor 7	Tumor 8
70	38	40	85	52	64	60	61
68	31	60	37	56	76	55	63
60	28	45	67	52	62	58	59
70	7	47	70	63	64	65	58
73	49	54	25	48	78	74	61
76	25	55	53	53	70	60	70
89	35	53	66	41	70	44	64
78	23	46	63	51	76	62	52
73	29.5	50	58.25	52	70	59.75	61
18.25	7.375	12.5	14.5625	13	17.5	14.9375	15.25
anti-miR-10b				control			
2	4	5	6	1	3	7	8
			13.17188				15.17188
**p**			4.537854				1.84375
0.22267			1.134464				0.460938

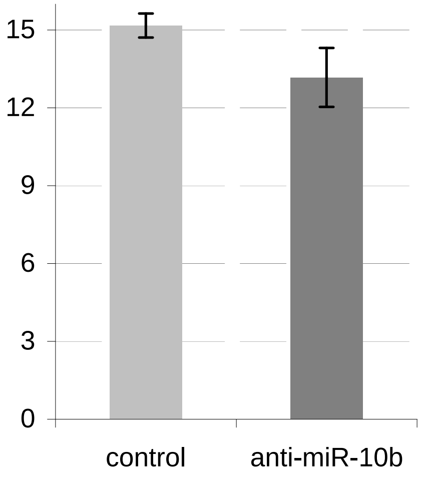

Supplement: Supplementary file 14 — Source Data for Figure 8 [file EMMM-8-268-s012.pdf]
