# Supplementary material for: Therapeutic potential of targeting microRNA‐10b in established intracranial glioblastoma: first steps toward the clinic
Source: EMBO Mol Med. 2016 Feb 10;8(3):268–87. doi: 10.15252/emmm.201505495 (PMC4772951; doi:10.15252/emmm.201505495)
Supplement: Supplementary file 15 — Source Data for Figure 9 [file EMMM-8-268-s013.pdf]

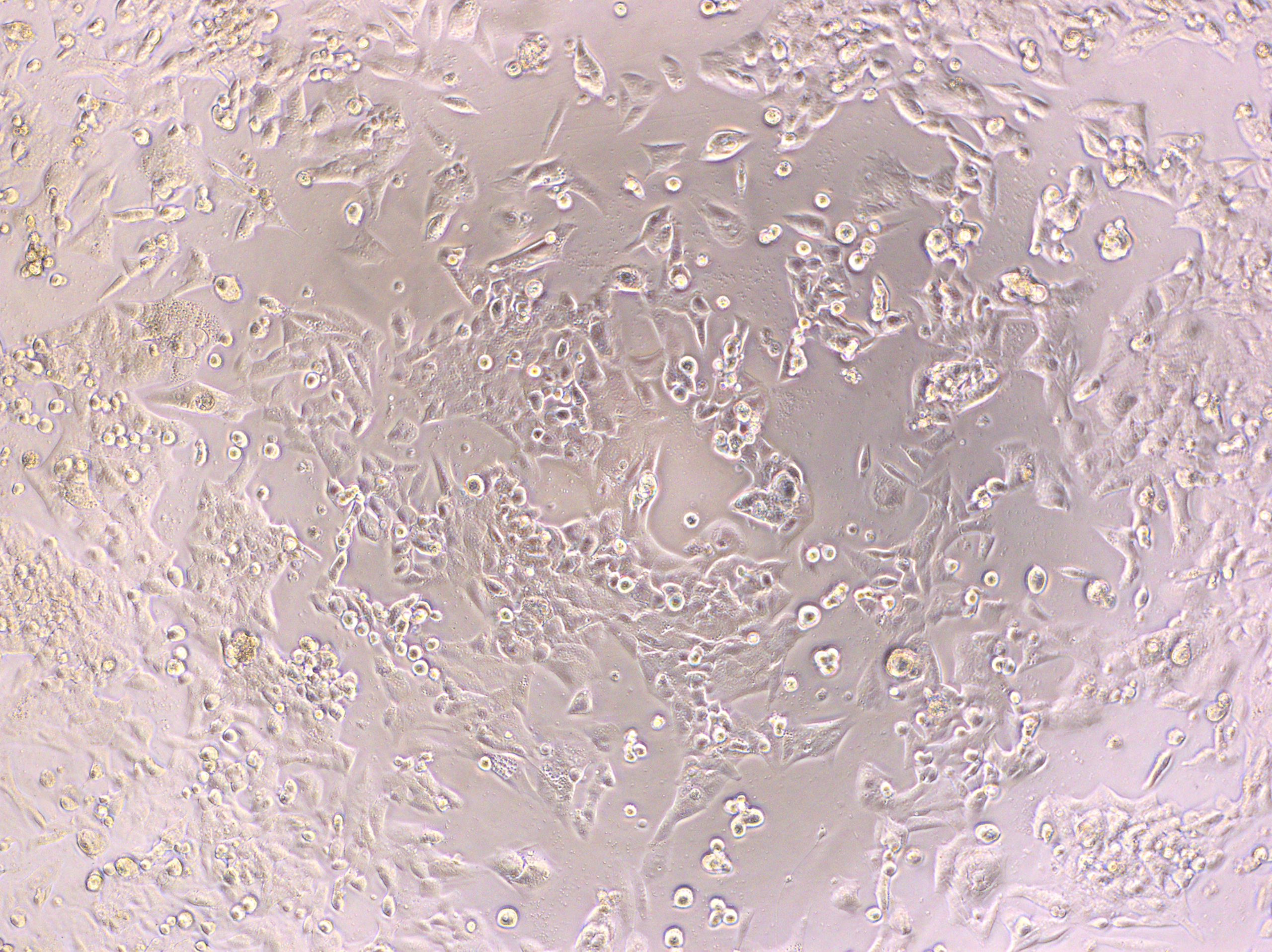

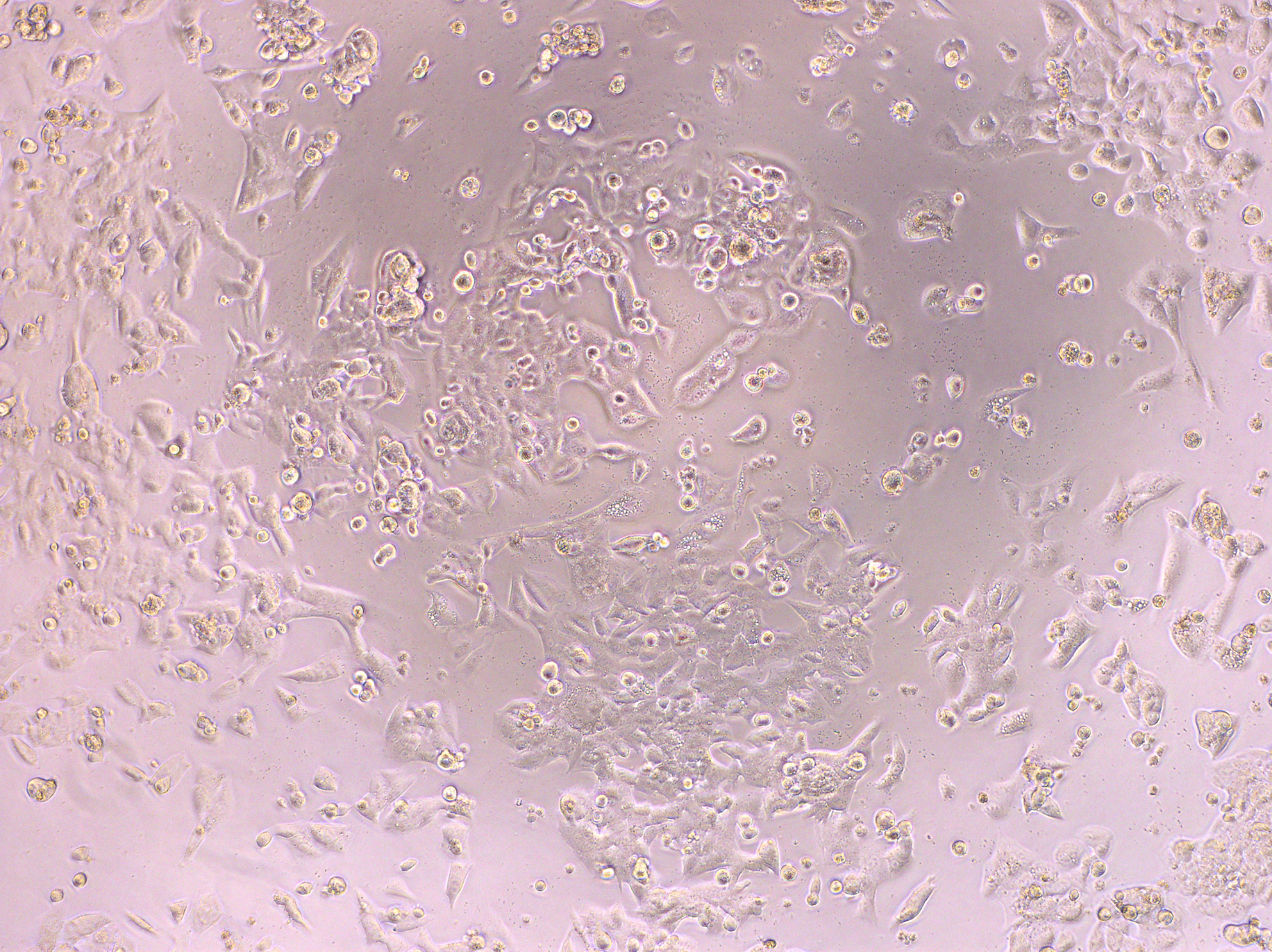

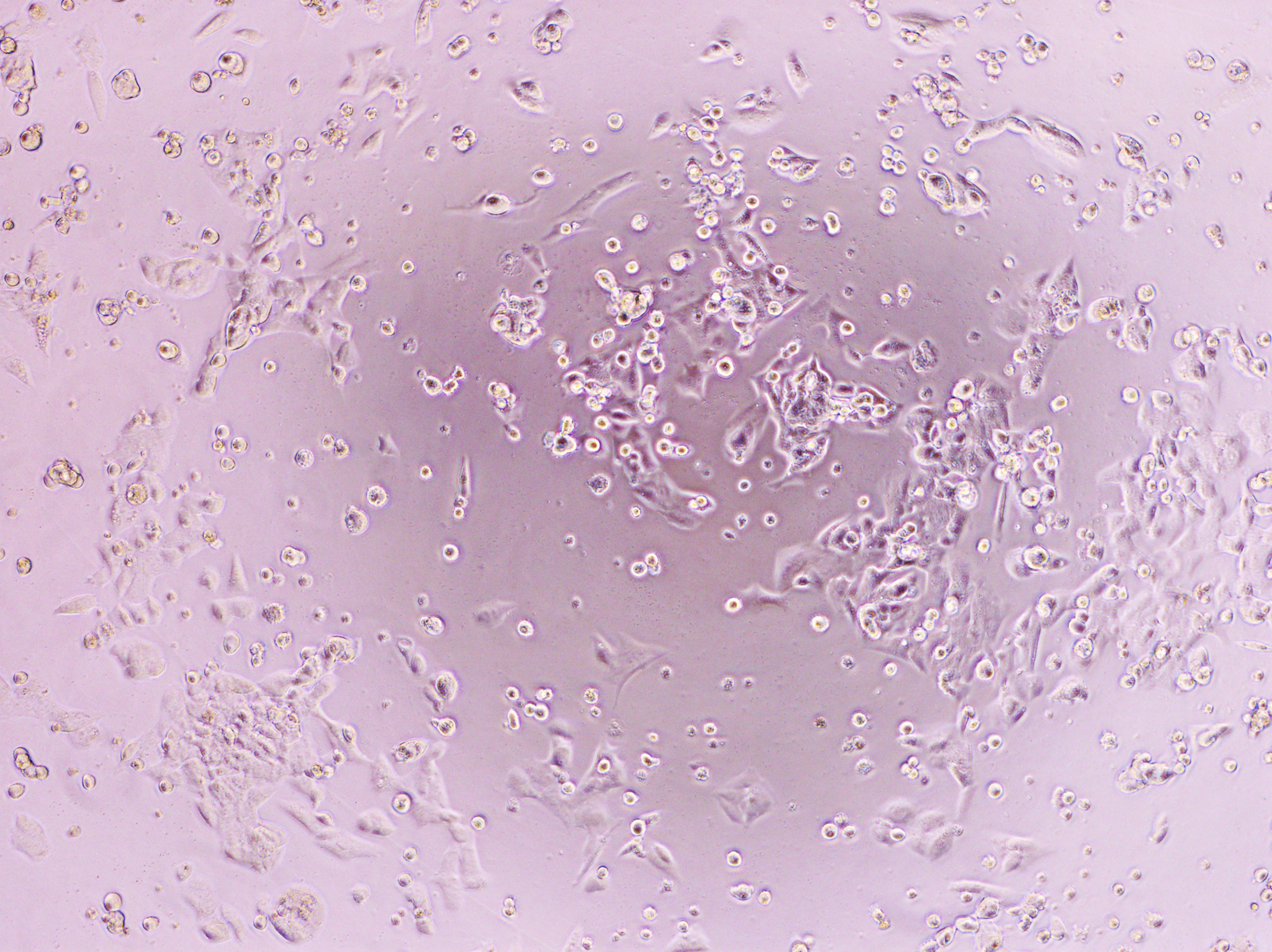

Figure 9 Panel B Source Data

**Day 3**						
665	1783	1071	1789	1194	2355	763
3895	339597	5246	250940	9018	364135	2383
4852	406899	6372	279647	8082	427859	2616
5206	425130	6670	401194	7839	430990	2535
5524	360685	6581	284990	8148	439469	2683
5134	380672	7234	330020	7770	385053	2690
5316	428722	6444	328796	7153	379844	2430
864	2621	1172	2504	1328	2835	721
	390284		312598		404558	
	36070.61		53037.4		31892.35	

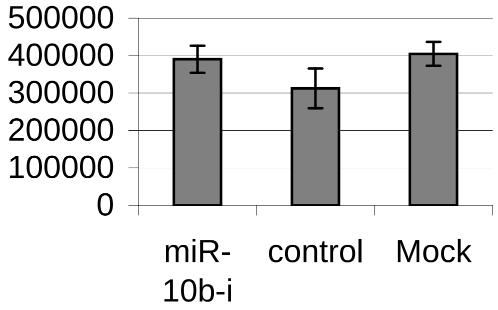

miR-10b-i
control
Mock

**Day 4**						
727	1940	1237	2732	1546	2626	1171
3875	284269	7240	536305	8344	432748	4730
3487	214781	7747	433345	9766	448615	5586
3800	223662	8191	449120	11464	639329	7023
4693	298857	8021	412039	12660	774401	8153
4683	327587	8445	428689	10610	499050	7459
4828	323220	8108	622700	10443	517642	7499
830	2343	1490	3552	1750	4190	1641
	278729		480366		551964	
	48834.62		82358.38		131135.2	

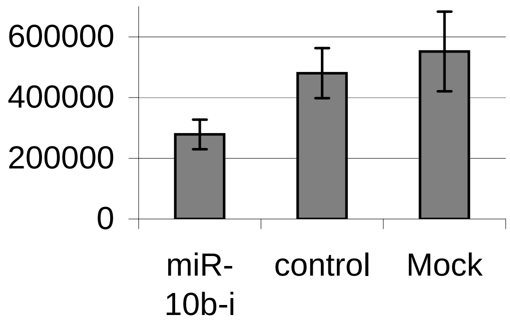

Figure 9 Panel B Source Data

**Day 5**						
999	2537	1834	4194	2123	3675	1043
5300	461941	10284	783238	11444	724478	3698
5665	356990	11335	719320	13370	724089	4456
6596	436500	12079	635452	14086	709431	4624
6494	401938	12364	654814	14880	893491	4849
6729	483187	11112	690048	14197	955647	4710
6119	548484	11035	860112	12499	805740	3700
1055	3064	1804	4673	2180	4448	1116
	448173		723831		802146	
	66432.08		84661.13		102606.7	

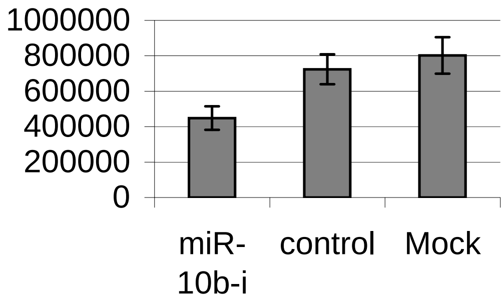

**Day 6**						
826	1900	1784	4206	2105	3639	992
3487	229201	10000	605088	10860	523640	3297
3312	186952	10790	654357	11961	529688	3839
4247	221376	10781	570025	12669	613827	4095
8185	600120	11986	590933	13570	704954	4371
6657	411225	12117	611373	12694	601672	3777
4864	322063	10005	586504	9798	505117	2950
912	2465	1803	4242	1966	3596	968
	328490		603047		579816	
	156226.7		29041.61		75554.55	

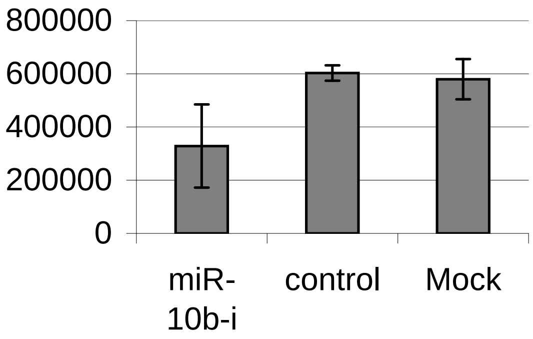

Figure 9 Panel B Source Data

Day 7					
1817	2889	1964	3701	1843	2614
7997	518901	9502	610430	8292	444326
8771	427415	10969	770958	10331	525845
8810	434717	11760	830207	12787	829290
9654	480920	12834	929575	12858	718542
10245	562796	13089	837725	13890	551316
7954	383289	11391	726453	13675	604508
1855	2868	2149	5012	2305	4414
	468006		784225		612305
	65840.68		109381.5		139886.8

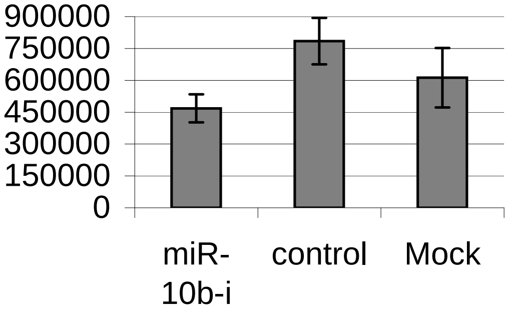

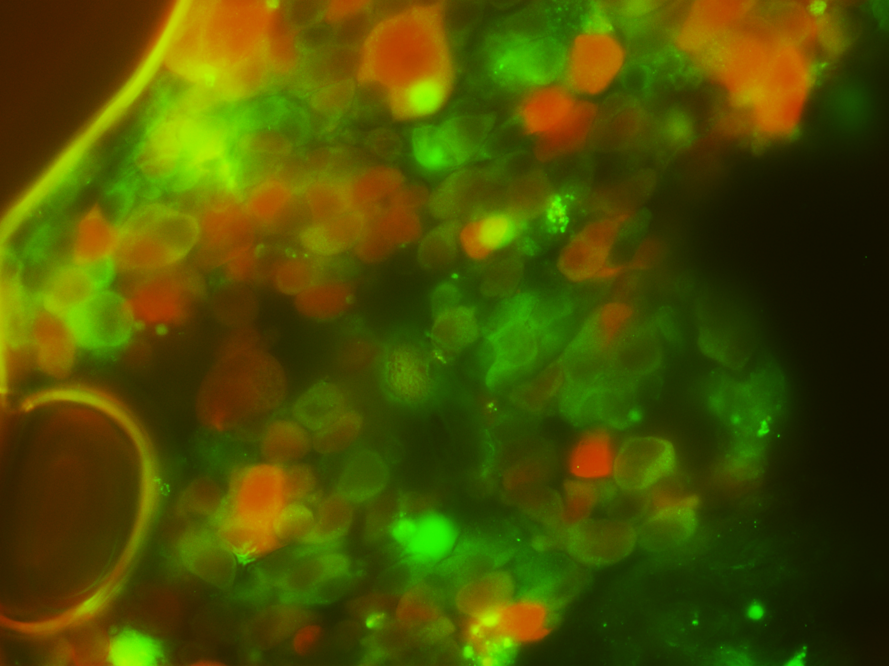

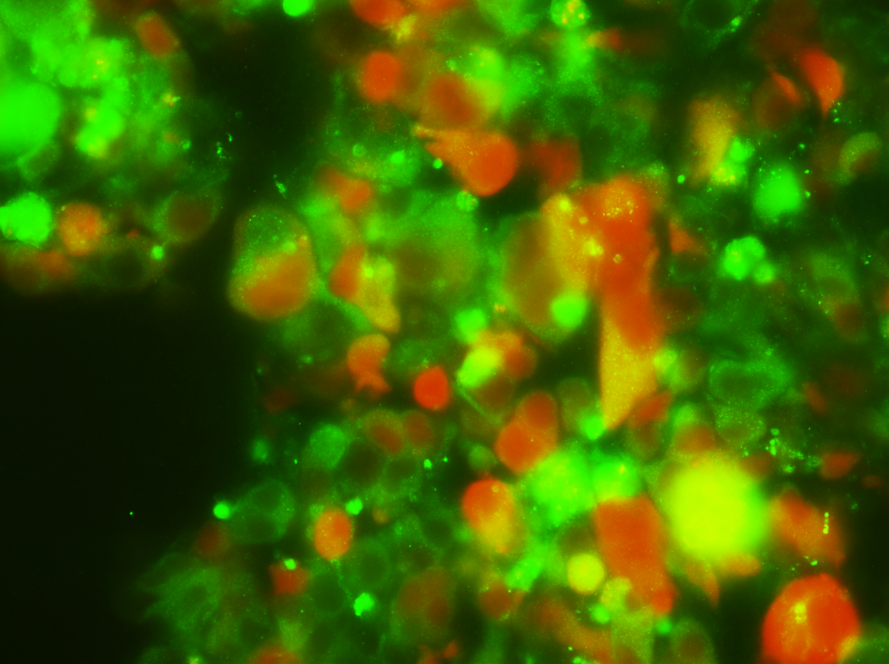

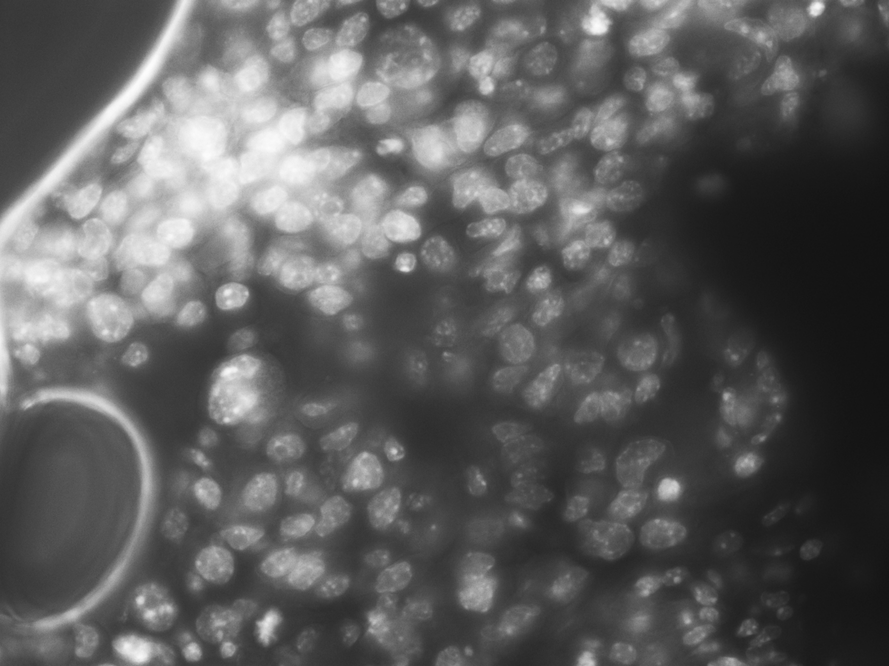

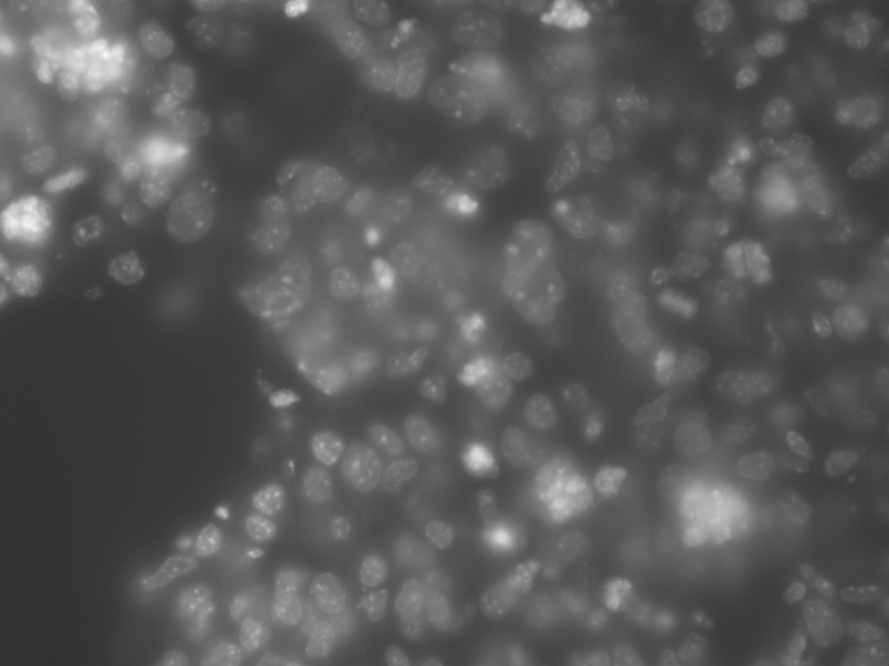

Figure 9 Panel D Source Data

Cage 1	**062013 Imaging day 3 after pumps implantation**				
Image Num	Total Flux [p/s]				
	**Day -7**	**Day -3**	Day 3	**Day 3/ Day -**	**Day -3/ Day -7**
miR-10b-i	675500.00	2111000.00	5788000.00	2.7418285	3.125092524
control	1001000.00	2518000.00	6975000.00	2.7700556	2.515484515
miR-10b-i	1011000.00	3169000.00	5142000.00	1.6225939	3.134520277
miR-10b-i	426200.00	801500.00	2089000.00	2.6063631	1.880572501
Cage 2					
miR-10b-i	2300000.00	15670000.00	19990000.00	1.275686	6.813043478
control	513300.00	826800.00	5924000.00	7.1649734	1.610753945
control	720400.00	2670000.00	21980000.00	8.2322097	3.706274292
control	1896000.00	6748000.00	15760000.00	2.3355068	3.55907173

	Average day3/ day -3			**Average Day -3/ day -7**		
	FC	SD	SE	FC	SD	SE
miR-10b-i	2.0616179	0.72338666	0.36169333	3.7383072	3.738307195	1.86915
control	5.1256864	3.00794932	1.50397466	2.8478961	2.847896121	1.42395

2.74	**p**
1.275686	0.0474569
1.62	*
2.606363	
2.770056	
7.164973	
8.23221	
2.335507	

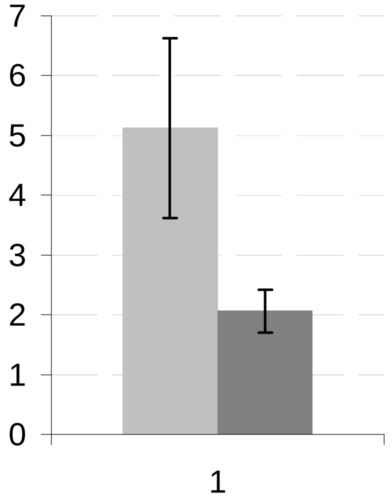

Figure 9 Panel E Source Data

		miR-10b				
miR-10b-i	mouse14	29.77	0.04	0.011218	0.012919	0.002404742
miR-10b-i	mouse14	29.855	0.03	0.014619		
miR-10b-i	mouse22	31.41	0.01	0.004681	0.004702	2.99611E-05
miR-10b-i	mouse22	31.557	0.01	0.004723		
miR-10b-i	mouse32	28.309	0.10	0.035427	0.041006	0.007889575
miR-10b-i	mouse32	28.109	0.12	0.046585		
miR-10b-i	mouse34	30.748	0.02	0.007391	0.006672	0.001016385
miR-10b-i	mouse34	31.24	0.01	0.005954		
miR-10b-i	mouse52	30.383	0.02	0.006	0.006804	0.001214025
miR-10b-i	mouse52	30.226	0.03	0.008		
Control	mouse44	25.972	0.51	0.126	0.1333	0.010507828
Control	mouse44	26.025	0.49	0.141		
Control	mouse53	26.184	0.44	0.095	0.101386	0.009131007
Control	mouse53	26.005	0.50	0.108		
Control	mouse62	25.659	0.63	0.148	0.144393	0.005730899
Control	mouse62	25.794	0.58	0.140		
Control	mouse73	26.085	0.47	0.118	0.130826	0.018469325
Control	mouse73	25.913	0.53	0.144		
Control	mouse81	25.159	0.90	0.183	0.181687	0.00151385
Control	mouse81	25.369	0.77	0.181		

			miR-10b			
miR-10b-i	mouse14	0.013	0.002404742	0.01	0.015178	0.006787984
miR-10b-i	mouse22	0.005	2.99611E-05			
miR-10b-i	mouse32	0.041	0.007889575	**p**		
miR-10b-i	mouse34	0.007	0.001016385	0.000015		
miR-10b-i	mouse52	0.007	0.001214025	***		
Control	mouse44	0.133	0.010507828	0.14	0.028996	0.012967311
Control	mouse53	0.101	0.009131007			
Control	mouse62	0.144	0.005730899			
Control	mouse73	0.131	0.018469325			
Control	mouse81	0.182	0.00151385			

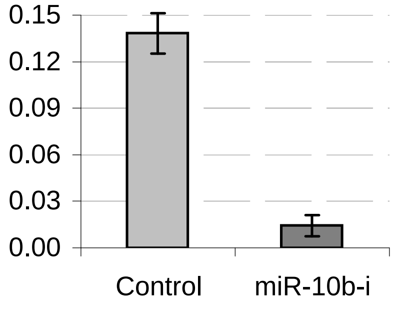

Figure 9 Panel E Source Data

miR-125b

23.292	3.27	2.82	0.638839
23.759	2.36		
23.671	2.51	2.380422	0.186483
23.831	2.25		
23.49	2.85	2.668063	0.254613
23.685	2.49		
23.668	2.52	2.369867	0.208807
23.848	2.22		
22.989	4.03	3.758824	0.38437
23.198	3.49		
22.982	4.05	3.771045	0.39481
23.196	3.49		
22.787	4.64	4.628363	0.011341
22.792	4.62		
22.907	4.27	4.187974	0.110832
22.961	4.11		
22.999	4.00	3.846872	0.220476
23.116	3.69		
22.707	4.90	4.593917	0.433912
22.9	4.29		

miR-125

2.815349	0.638839	2.80	0.569657	0.254758
2.380422	0.186483			
2.668063	0.254613			
2.369867	0.208807			
3.758824	0.38437			
3.771045	0.39481	4.21	0.402291	0.17991
4.628363	0.011341			
4.187974	0.110832			
3.846872	0.220476			
4.593917	0.433912			

Figure 9 Panel F Source Data

	p21					GAPDH	
GL261 48h	20.087	241.019	356.736	409.829	40.483	15.278	0.676
GL261 48h	19.993	257.245	455.312			15.536	0.565
GL261 48h	19.966	262.105	413.491			15.370	0.634
GL261 48h	20.097	239.354	413.778			15.502	0.578
GL261 48h	19.103	476.720	682.512	728.761	48.080	15.230	0.698
GL261 48h	18.945	531.896	786.178			15.276	0.677
GL261 48h	18.847	569.282	696.371			15.003	0.817
GL261 48h	18.877	557.566	749.982			15.140	0.743
GL261 48h	20.014	253.528	359.217	384.306	22.727	15.215	0.706
GL261 48h	19.977	260.114	371.885			15.228	0.699
GL261 48h	19.970	261.379	408.648			15.357	0.640
GL261 48h	19.976	260.294	397.473			15.323	0.655

p

0.000027

***

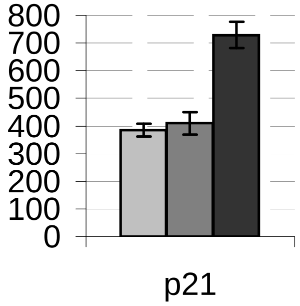

		p21	
Ctrl	48h	409.829	40.483
a-10b	48h	728.761	48.080
Mock	48h	384.306	22.727
